# miRNA-1246 induces pro-inflammatory responses in mesenchymal stem/stromal cells by regulating PKA and PP2A

**DOI:** 10.18632/oncotarget.14915

**Published:** 2017-01-31

**Authors:** Alexander Bott, Nese Erdem, Shalom Lerrer, Agnes Hotz-Wagenblatt, Christian Breunig, Khalid Abnaof, Angelika Wörner, Heike Wilhelm, Ewald Münstermann, Adit Ben-Baruch, Stefan Wiemann

**Affiliations:** ^1^ Division of Molecular Genome Analysis, German Cancer Research Center (DKFZ), Heidelberg, Germany; ^2^ Department of Cell Research and Immunology, Tel Aviv University, Tel Aviv, Israel; ^3^ Bioinformatics Group, Genomics & Proteomics Core Facility (GPCF), German Cancer Research Center (DKFZ), Heidelberg, Germany

**Keywords:** breast cancer, tumor microenvironment, mesenchymal stem/stromal cell, microRNA, NF-kappaB signaling

## Abstract

The tumor microenvironment (TME) has an impact on breast cancer progression by creating a pro-inflammatory milieu within the tumor. However, little is known about the roles of miRNAs in cells of the TME during this process. We identified six putative oncomiRs in a breast cancer dataset, all strongly correlating with poor overall patient survival. Out of the six candidates, miR-1246 was upregulated in aggressive breast cancer subtypes and expressed at highest levels in mesenchymal stem/stroma cells (MSCs). Functionally, miR-1246 led to a p65-dependent increase in transcription and release of pro-inflammatory mediators IL-6, CCL2 and CCL5 in MSCs, and increased NF-κB activity. The pro-inflammatory phenotype of miR-1246 in MSCs was independent of TNFα stimulations and mediated by direct targeting of the tumor-suppressors PRKAR1A and PPP2CB. *In vitro* recapitulation of the TME revealed increased Stat3 phosphorylation in breast epithelial (MCF10A) and cancer cells (SK-BR-3, MCF7, T47D) upon incubation with conditioned medium (CM) of MSCs overexpressing miR-1246. Additionally, this stimulation enhanced proliferation of MCF10A cells, increased migration of MDA-MB-231 cells and induced attraction of THP-1 monocytic cells. Our data shows that miR-1246 acts as both key-enhancer of pro-inflammatory responses in MSCs and putative oncomiR in breast cancer, suggesting its influence on cancer-related inflammation and breast cancer progression.

## INTRODUCTION

Breast cancer is the most frequently diagnosed cancer in women and the second leading cause of malignancy-related death of women in the US [1]. During cancer progression, the tumor microenvironment (TME) undergoes dramatic changes and has been accepted as a major driver of malignancy [2, 3]. Heterogeneity and plasticity strongly influence patient survival, mainly due to changes in the secretome profiles of different cell entities in the TME [4]. Here, cancer-associated fibroblasts (CAFs), tumor-associated macrophages (TAMs) and mesenchymal stem/stromal cells (MSCs) are known to secrete many tumor-promoting and pro-inflammatory factors [5–7]. Under physiological conditions, MSCs are involved in tissue regeneration, control of immune response, angiogenesis and hematopoiesis, but in the context of cancer the homing of MSCs to the tumor is one predominant feature [8, 9]. Once integrated into the tumor stroma, MSCs differentiate into CAFs and form up-to 20% of their population [10–14]. Cancer associated MSCs take over multifaceted tumor-promoting roles. They contribute to stromal remodeling, angiogenesis and lymphangiogenesis, as well as to tumor growth and aggressiveness [15–18]. Several studies have shown that MSCs also have a tremendous impact on tumor-associated inflammation [19–21].

Clinical data reveals that chronic inflammation correlates with decreased breast cancer patient survival [22]. NF-κB signaling is one of the main pro-inflammatory signaling pathways and strongly links inflammation to breast cancer [23–27]. Induction of NF-κB signaling is triggered by TNFα, which is mainly released by TAMs and cancer cells [28–30]. In fact, TNFα is enriched in the tumor stroma [31] and activation of NF-κB signaling leads to transcription and secretion of inflammatory cytokines and chemokines as mediators of tumor progression [32, 33]. In this context, MSCs respond to TNFα but do not produce it, as result of epigenetic silencing of the TNFα promoter [34]. Stimulation of MSCs with TNFα *in vitro* mimics a TME-activated MSC secretion profile of pro-inflammatory mediators [19, 35, 36]. However, MSCs release various growth factors, cytokines and chemokines even in the absence of pro-inflammatory stimuli. IL-6 and the inflammatory chemokines CCL2 and CCL5 are among the most prominent [37]. IL-6 induces EMT and links NF-κB to Jak-Stat signaling by triggering Stat3 phosphorylation. This is connected to breast cancer growth and aggressiveness, as well as to poor patient prognosis [38–41]. CCL2 leads to recruitment of various myeloid cells *via* the CCL2/CCR2 axis. This results in high presence of TAMs and myeloid-derived suppressor cells in tumors [42, 43] and thereby massively promotes tumor progression [33, 44]. At last, MSC-released CCL5 has been linked to invasion of cancer cells and lung metastasis formation [17, 45]. Overall, MSCs affect different hallmarks of cancer [46] and have major roles in promoting cancer-related inflammation.

NF-κB signaling is strongly influenced by post translational modifications including phosphorylation and dephosphorylation by kinases and phosphatases, respectively [47]. cAMP-dependent protein kinase A (PKA) is a Ser/Thr kinase and forms a tetrameric holoenzyme involving different regulatory and catalytic subunits [48]. In its inactive state the regulatory subunits bind to and inhibit the catalytic subunits [49]. cAMP-dependent protein kinase type I-alpha regulatory subunit (PRKAR1A) is one of the most significant regulatory subunits. PRKAR1A knock-down leads to constitutive PKA activation [50], and knock-out to early embryonic lethality [51]. While kinases are frequently activators of molecular processes, they are often antagonized by protein phosphatases (PPPs) [52]. Serine/Threonine-protein phosphatase 2A (PP2A) forms a subfamily of PPPs and is besides PP1 one of the major Ser/Thr phosphatases in eukaryotic cells [53]. The heterotrimeric holoenzyme is comprised of one regulatory, one catalytic and one scaffolding subunit each [54]. The PP2A catalytic subunit is represented either by the α (PPP2CA) or the β (PPP2CB) isoform [55]. PP2A has been described as a negative master-regulator of inflammatory signaling *via* inhibition of several MAPKs [56, 57]. In these studies, regulatory subunits have been linked to signaling activity, whereas the potential role of catalytic subunits of PP2A as effectors of inflammatory signaling activity has not been described thus far.

miRNAs are small non-coding RNA molecules (~22 nucleotides), influencing gene expression at the posttranscriptional level. They target specific mRNAs by complementarity of their seed sequence to the mRNA 3’untranslated region (3’UTR) which leads to translational inhibition or mRNA degradation [58]. A complex system of miRNA-mediated post-transcriptional regulations can be achieved, as every miRNA may target several mRNAs and single genes can be targeted by many miRNAs [59]. miRNAs have been vastly described as oncogenic (oncomiRs) or tumor suppressive in several cancer types including breast cancer [58, 60–62]. In MSCs, miRNAs have mainly been shown to regulate cell differentiation [63, 64], while little is known about their impact on secretion of pro-inflammatory cytokines [65]. Only few studies have addressed the function of miRNAs in MSCs in the context of inflammation [66, 67]. One finding is that miR-126 leads to MSC recruitment [68], and also promotes cell survival and secretion of pro-angiogenic factors in MSCs [69].

The aim of this study was to unravel novel miRNA-mediated mechanisms in the pro-inflammatory regulation of the TME by uncovering molecular functions of miRNAs in MSCs, and discerning their impact on protein secretion and cancer-related inflammation. To this end, miRNA expression levels of breast cancer relevant miRNAs were quantified in MSCs. miR-1246 was identified as critical regulator of NF-κB signaling, which increases pro-inflammatory responses in MSCs and thereby impacts on different cell types, including breast cancer cells.

## RESULTS

### miR-1246 expression in breast cancer and MSCs

**Figure 1:**
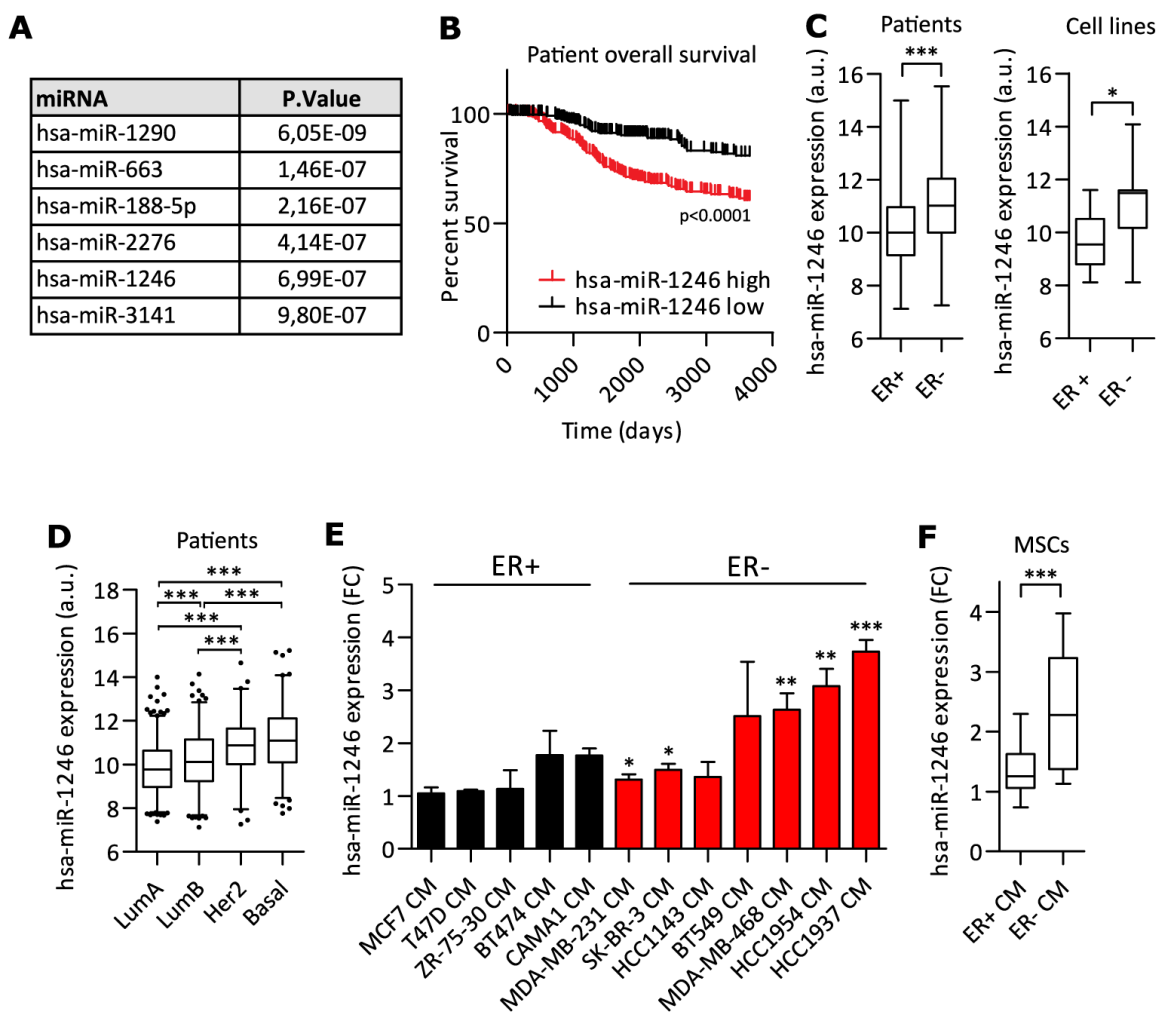
miR-1246 expression and regulation in MSCs and breast cancer **A.** miRNAs were ranked according to their significance of correlating with negative breast cancer patient survival (see Supplementary Table 1). A stringent p-value of < 1.0e-6 was applied to identify top six miRNAs with impact on negative breast cancer patient survival. **B.** Kaplan-Meier analysis was performed with miR-1246 using the METABRIC breast cancer dataset with n=257 for each quartile. **C.** miR-1246 expression analysis in ER+ vs. ER- patients (ER-: n=267, ER+: n=993) or cell lines (ER-: n= 12, ER+: n=8) (cell line specific expression in Supplementary Figure 1C). Data was retrieved from METABRIC dataset [70]. **D.** miR-1246 expression analysis according to molecular breast cancer subtypes Luminal A (LumA, n=481), Luminal B (LumB, n=315), Her2 positive (Her2, n=127) and basal like (Basal, n=211). Data was retrieved from METABRIC dataset [70]. **E.** and **F.** Regulation of hsa-miR-1246 expression in MSCs by CM of ER- and ER+ breast cancer cells. MSCs were starved o.n. and stimulated with cell line specific growth medium or conditioned medium (CM) for 14h with n=3 and hsa-miR-1246 expression was analyzed by qRT-PCR. Data was normalized and compared to expression of miR-1246 after stimulation with cell line-specific growth medium and is presented as bar chart for the specific cell lines (**E**) or as box plot for the groups ER+ versus ER- CM (**F**). Data is presented as mean ± SD. * represents p < 0.05; ** represents p < 0.01; *** represents p < 0.001.

The METABRIC miRNA-expression data [70] was analyzed to identify miRNAs significantly correlating with overall patient survival in breast cancer (Supplementary Table 1). miRNAs were filtered by significance to identify top miRNAs negatively correlating with breast cancer patient survival. hsa-miR-1290, hsa-miR-663, hsa-miR-188-5p, hsa-miR-2276, hsa-miR-1246 and hsa-miR-3141 met the most stringent conditions of p < 1.0e-6 (Figure 1A). To analyze their expression in cells forming part of the TME, human BM-derived MSCs were sequenced and their miRNA expression levels were quantified (Supplementary Table 2). Out of the six miRNA candidates, the expression of hsa-miR-1246 was highest in MSCs, followed by hsa-miR-188-5p and hsa-miR-2276-3p. hsa-miR-663a, hsa-miR-3141 and hsa-miR-1290 were not expressed in MSCs (Supplementary Figure 1A).

We hypothesized a subtype-specific expression of miR-1246 in breast cancer as miR-1246 significantly correlated with poor overall breast cancer patient survival (Figure 1B). Since ER expression is itself a critical predictor of breast cancer patient survival (Supplementary Figure 1B), we analyzed if miR-1246 was differentially expressed in ER- compared to ER+ breast cancer subtypes. Indeed, miR-1246 showed significant higher expressions in the ER- subtype compared to the ER+ subtype in patient (Figure 1C) and cell line (Figure 1C, Supplementary Figure 1C) data from the METABRIC dataset [70]. Further, miR-1246 was higher expressed in the more aggressive molecular breast cancer subtypes (Basal, Her2+, Luminal B) compared to the less aggressive Luminal A subtype (Figure 1D).

Next, we wanted to investigate if more aggressive ER- cancer cells could influence the expression of oncomiRs such as miR-1246 in other cell types of the TME. To this end, MSCs were stimulated with CM of ER+ breast cancer cell lines MCF7, T47D, BT474, ZR-75-30 and CAMA1 or ER- breast cancer cell lines MDA-MB-231, MDA-MB-468, SK-BR-3, BT549, HCC1143, HCC1954 and HCC1937 (Figure 1E). CM derived from ER- breast cancer cells almost consistently resulted in significant upregulation of miR-1246 in MSCs, whereas CM of ER+ cells had no regulatory effect on miR-1246 transcription. Overall, CM of ER- breast cancer cells increased expression of miR-1246 at significant higher levels than CM of ER+ breast cancer cells (Figure 1F).

We wanted to exclude that miR-1246 gets transferred from cancer cells to MSCs *via* exosomes or microvesicles and separated both vesicle types from the protein fraction of MDA-MB-468 cell-derived CM by ultracentrifugation. Stimulations of MSCs with the resuspended pellet did not affect miR-1246 expressions in MSCs, whereas stimulations with residual supernatants, containing the protein fractions, consistently increased its transcription (Supplementary Figure 1D). Next, we deprived the CM of MDA-MB-468 cells from proteins larger than 4 kDa before stimulation of MSCs. By this, we wanted to prove that the deprived protein fraction is responsible for induction of miR-1246 transcription and to further exclude the transfer or detection of free RNA. Indeed, the protein-deprived CM did not increase miR-1246 expression in MSCs compared to control conditions (Supplementary Figure 1E). Based on these findings, we hypothesized that miR-1246 regulation in MSCs by CM of MDA-MB-468 cells is mediated by transcriptional induction and not *via* transfer of free RNA from cancer cells to MSCs.

### miR-1246 influences NF-κB signaling in MSCs

**Figure 2:**
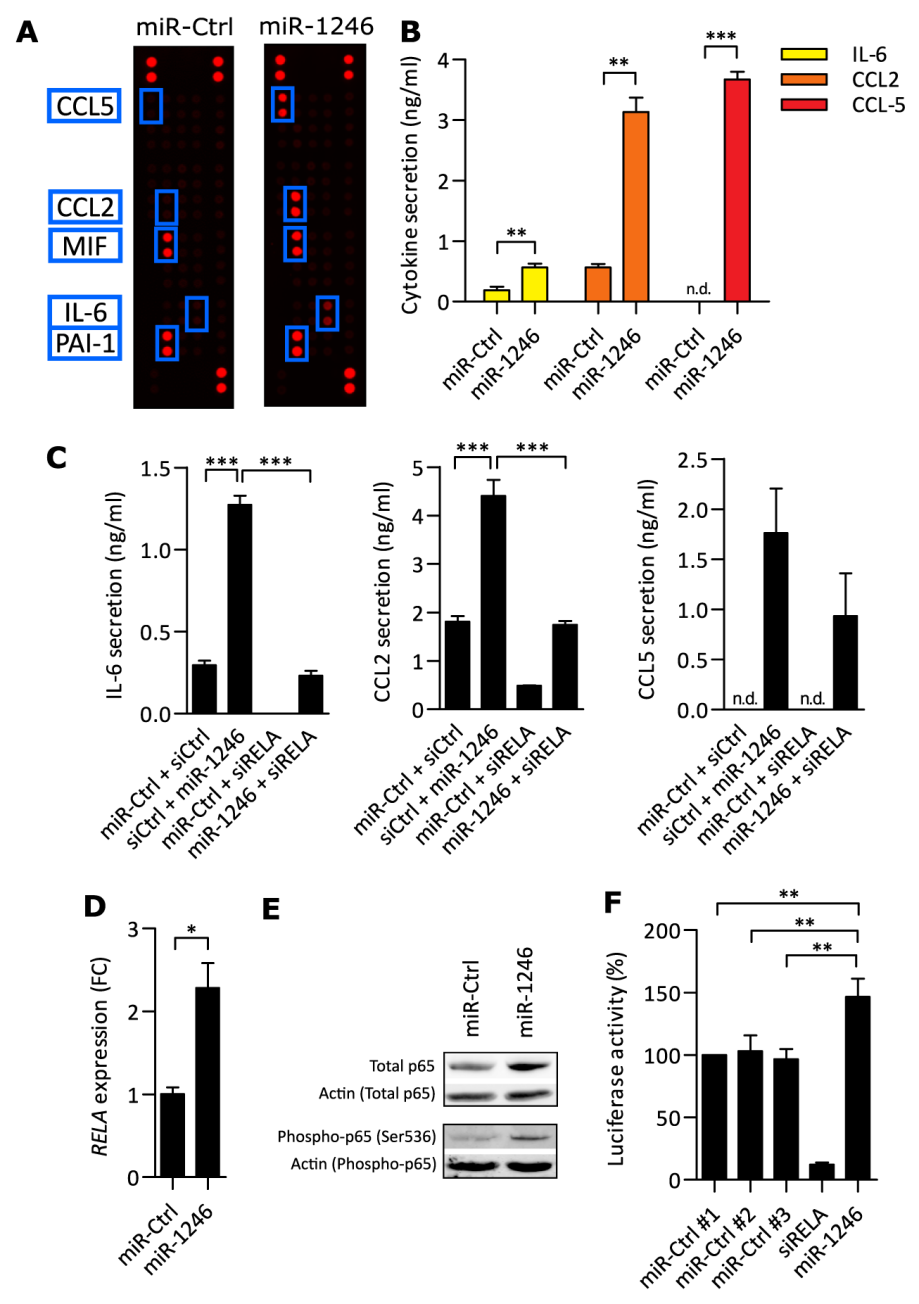
miR-1246 increases pro-inflammatory responses in MSCs **A.** CM of miR-Ctrl transfected MSCs, compared to CM of miR-1246 overexpressing MSCs was analyzed on a human cytokine array. Presented is one out of two scanned membranes for each condition. Blue boxes indicate the detected protein, labeled on the left of the scan. **B.** FLISA quantification of MSC CM after miR-Ctrl or miR-1246 transfection. CCL5 was not detectable (n.d.) after miR-Ctrl transfection. Data is presented as mean ± SD, n=3. **C.** Quantification of IL-6, CCL2 and CCL5 in MSC CM with FLISA. Data is presented as mean ± SD, n=3. **D.** qRT-PCR based analysis of *RELA*, 48h after miR-1246 or miR-Ctrl overexpression in MSCs. Data was normalized to miR-Ctrl and is presented as mean ± SD, n=3. **E.** Western blot analysis of MSCs transfected for 72h. Analyzed proteins were total p65 and phospho-p65 (Ser536), using β-Actin as loading control, n=6 (Quantification in Supplementary Figure 4). **F.** Luciferase activity of HEK293-FT cells transfected with miR-1246 in combination with a NF-κB luciferase expressing plasmid. Knock-down of *RELA* was used as positive control. Data is presented as mean ± SD, n=4. * represents p < 0.05; ** represents p < 0.01; *** represents p < 0.001.

Based on our finding of miR-1246 regulation by CM of ER- breast cancer cell lines, we wanted to reveal the biological functions of miR-1246 in MSCs. Since MSCs are known to secrete pro-inflammatory cytokines and chemokines into the TME, miR-1246 or miR-Ctrl was transfected into MSCs (Supplementary Figure 2) and the CM was screened for secreted proteins using a cytokine array. PAI-1, CCL2, MIF, CCL5 and IL-6 were detected in CM of at least one condition (Figure 2A, Supplementary Table 3) and all except PAI-1 were released at significant higher levels after miR-1246 overexpression compared to the control (Supplementary Figure 3A). As miR-Ctrl transfected MSCs showed strong baseline secretion levels of PAI-1 and MIF, only the miR-1246-induced release of IL-6, CCL2 and CCL5 was validated at protein (Figure 2B) and mRNA levels (Supplementary Figure 3B). Notably, CCL5 was induced dramatically upon miR-1246 overexpression and was not detected in CM of MSCs under control conditions.

NF-κB signaling has been shown to regulate the expression of many pro-inflammatory cytokines and chemokines and we wanted to investigate whether miR-1246 regulates IL-6, CCL2 and CCL5 expression in a NF-κB-dependent manner. To this end, miR-1246 was overexpressed in combination with knock-down of *RELA*, the gene coding for p65 NF-κB subunit, and inflammatory mediators were subsequently quantified in the CM. Indeed, miR-1246-induced increases in IL-6 and CCL2 expression were completely blocked when *RELA* was knocked-down in parallel (Figure 2C). This proves that miR-1246 mediates IL-6 and CCL2 production in a p65-dependent manner and that it acts independent of an additional pro-inflammatory stimulus in MSCs. However, release of CCL5 was not decreased significantly upon overexpression of miR-1246 in combination with *RELA* knock-down, compared to miR-1246 overexpression in MSCs. This indicates that miR-1246 regulates CCL5 transcription by targeting other signaling pathways than NF-κB in MSCs. Next, we investigated whether p65 levels were influenced by miR-1246 since we showed that pro-inflammatory activities of miR-1246 require p65. Consequently, we quantified *RELA* mRNA, p65 as well as phosphorylation of p65 (phospho-p65) at Serine536 (Ser536) at protein levels after miR-1246 overexpression. Significant increases in all three were detected after miR-1246 overexpression in MSCs (Figure 2D and 2E, Supplementary Figure 4A). Differential protein isolations of either the nuclear or the cytoplasmic fraction were performed to investigate on the cellular localization of p65 after miR-1246 overexpression in MSCs. Indeed, miR-1246 significantly increased levels of total and phospho-p65 in both cytoplasmic and nuclear protein fractions (Supplementary Figure 4B and 4C).

Based on these results, we hypothesized that miR-1246 affects NF-κB activities and used a luciferase-based NF-κB reporter assay in HEK293-FT cells to quantify NF-κB signaling activity after miR-1246 overexpression. Indeed, miR-1246 significantly elevated NF-κB activities by 50% compared to three different miRNA controls. Knock-down of *RELA* was used as positive control (Figure 2F).

### miR-1246 directly targets PRKAR1A and PPP2CB in MSCs

**Figure 3:**
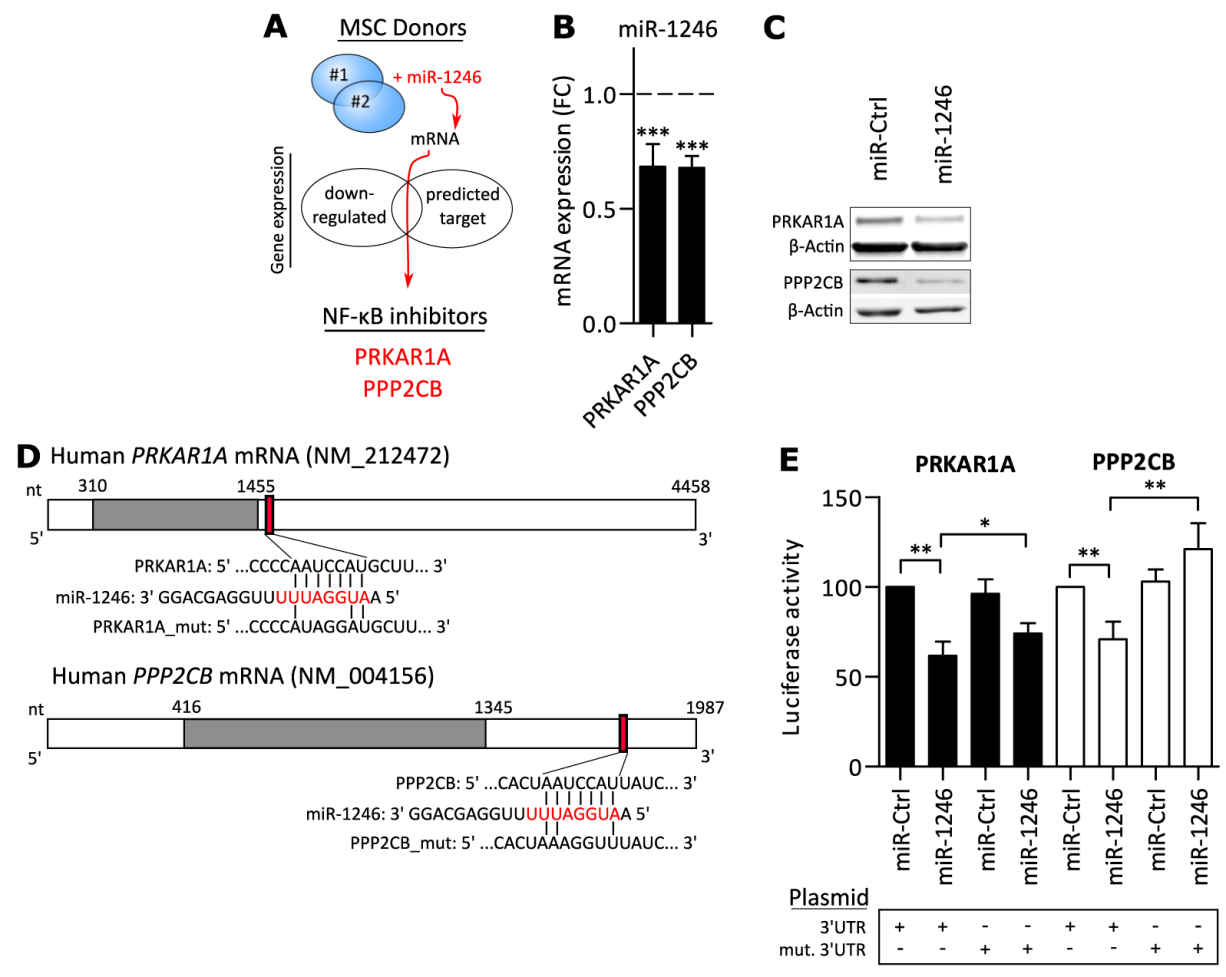
miR-1246 directly targets PRKAR1A and PPP2CB **A.** Schematic overview of miR-1246 target gene identification. MSCs of two different donors were transfected with miR-1246 or miR-Ctrl and differential mRNA analysis was performed on the genome-wide level. Candidates were defined by significant down-regulation after miR-1246 overexpression and by additionally being a predicted target of miR-1246 [71] (Candidates listed in Supplementary Table 4). At last, previously reported negative regulators of NF-κB signaling were selected for further analyses. **B.** Array-based mRNA expression 48h after miR-1246 overexpression in MSCs, compared to miR-Ctrl transfection (dashed line). Data is presented as mean ± SD, n=6, representing MSCs of two donors. **C.** Western blot analysis after miR-1246 overexpression in MSCs, compared to miR-Ctrl. β-Actin was used as loading control. Shown are representative Western blots for n=9, representing results of three different MSC donors (Quantifications in Supplementary Figure 5B). **D.** Schematic overview of *PRKAR1A* and *PPP2CB* (Grey box = open reading frame; red box = predicted miR-1246 binding site in 3’UTR; red nucleotides = seed sequence; connecting bars = potential binding complementarity). **E.** Luciferase activity after plasmid overexpression in combination with either miR-1246 or miR-Ctrl. Data was normalized to miR-Ctrl + empty vector transfections and presented as mean ± SD, n=4. * represents p < 0.05; ** represents p < 0.01; *** represents p < 0.001.

We hypothesized that miR-1246 directly targets genes that are involved in negative regulation of basal NF-κB activity. Therefore, miR-1246 was overexpressed in MSCs of two different donors and genome-wide mRNA expression was compared to a control transfection to identify direct targets of miR-1246. Candidate genes were identified by the combination of two parameters: Being significantly down-regulated after miR-1246 overexpression in both individuals (> 20% reduction) and additionally by being a predicted target of miR-1246 [71] (Supplementary Table 4). Subsequently, a final selection for previously reported negative regulators of NF-κB signaling identified *PRKAR1A* and *PPP2CB* as potential targets of miR-1246 (Figure 3A). Array-based gene expression analysis showed that both genes were significantly downregulated by miR-1246 in MSCs (Figure 3B). Downregulations of *PRKAR1A* and *PPP2CB* were validated on mRNA (Supplementary Figure 5A) and protein levels in MSCs of three different donors (Figure 3C, Supplementary Figure 5B).

To prove that *PRKAR1A* and *PPP2CB* are direct targets of miR-1246, their 3’UTRs were cloned into luciferase reporter plasmids and both plasmid and miR-1246 were co-transfected into MCF7 breast cancer cells. miR-1246 significantly decreased luciferase signals for *PRKAR1A* or *PPP2CB* 3’UTR carrying plasmid constructs, compared to miR-Ctrl. These decreases were significantly increased by mutating the predicted binding site of miR-1246 in either 3’UTR, demonstrating that miR-1246 directly targets *PRKAR1A* and *PPP2CB* at the predicted binding sites (Figure 3D and 3E).

**Figure 4:**
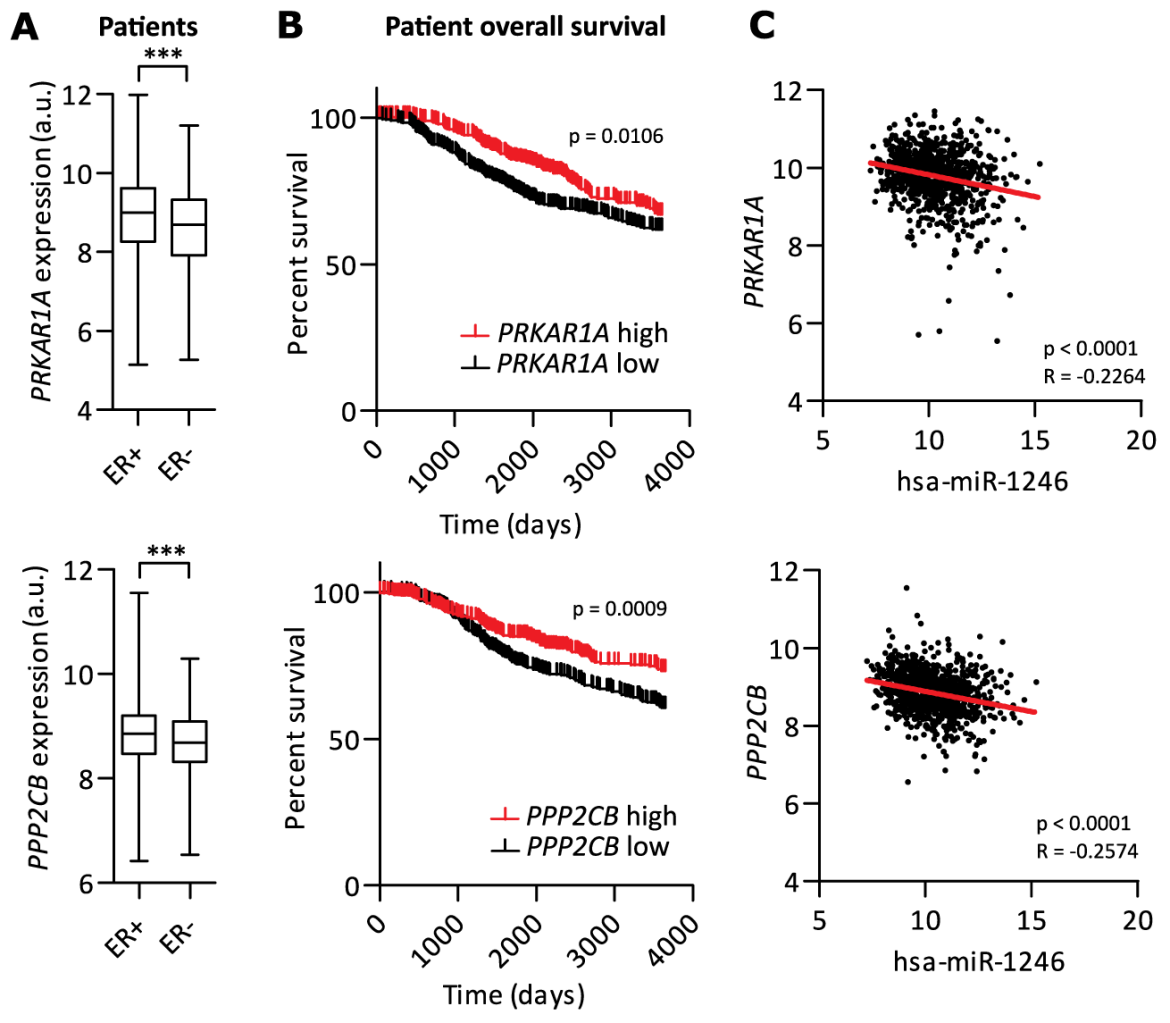
PRKAR1A and PPP2CB are potential tumor-suppressor genes in breast cancer **A.** mRNA expression analysis of the METABRIC dataset [72] of *PRKAR1A* or *PPP2CB*, comparing ER- (n=440) with ER+ (n=1508) patients. **B.** Quartile based survival analysis of the METABRIC dataset [72] of *PRKAR1A* or *PPP2CB*. Compared were high vs. low gene expressions with n=397 per quartile. **C.** Correlation analysis of the METABRIC dataset [72] of miR-1246 expression with either *PRKAR1A* or *PPP2CB* expression (n=873 was limited by patients expressing miR-1246 in both analyses). Correlation coefficient (R) and significance were calculated using Spearman correlation. Analyzed Illumina probe IDs for each gene were ILMN_1738632 for *PRKAR1A* and ILMN for *PPP2CB*. *** represents p < 0.001.

Since miR-1246 was expressed higher in ER- breast cancer subtypes and induced pro-inflammatory responses in MSCs by targeting *PRKAR1A* and *PPP2CB*, we hypothesized potential roles of *PRKAR1A* and *PPP2CB* as tumor-suppressors in breast cancer. To this end, their expression was compared within the subgroups of ER- and ER+ breast cancer patients of the METABRIC dataset [72]. Indeed, both genes were shown to be significantly lower expressed in ER- patients (Figure 4A) and low expression significantly correlated with poor overall breast cancer patient survival (Figure 4B). In addition, *PRKAR1A* and *PPP2CB* negatively correlated with miR-1246 expression (Figure 4C), indicating that miR-1246 could regulate both targets in tumors of breast cancer patients.

### PRKAR1A acts pro-inflammatory in MSCs

**Figure 5:**
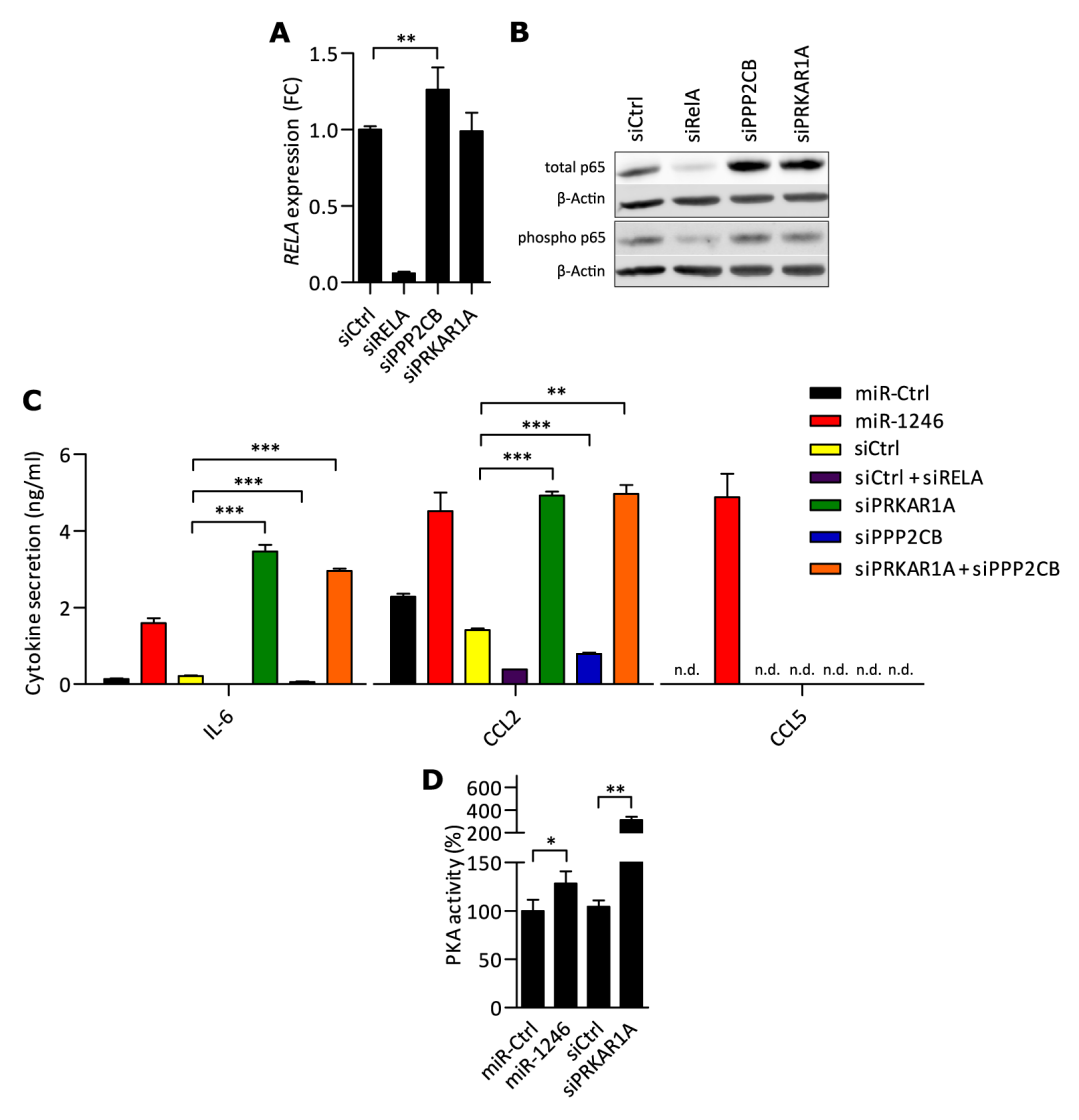
PKA acts pro-inflammatory in MSCs and miR-1246 increases PKA activity **A.** *RELA* expression analysis by qRT-PCR after *PPP2CB*, *PRKAR1A* or *RELA* knock-down in MSCs. Data was normalized to siCtrl and is presented as mean ± SD with n=6, representing data of two MSC donors. **B.** MSC total p65 and phospho-p65 (Ser536) protein expression analysis by Western blot with β-Actin as loading control. Presented blots represent n=6, performed in MSCs of two different donors (quantifications in Supplementary Figure 7B). **C.** FLISA analysis of soluble factors IL-6, CCL2 and CCL5 in the CM of MSCs after gene specific knock-down compared to siCtrl. miR-1246 compared to miR-Ctrl was used as positive control. Final concentrations of siRNAs were 30nM, also when different siRNAs were pooled. Data is presented as mean ± SD, n=3. **D.** PKA kinase activity after miR-1246 overexpression and si*PRKAR1A*, compared to the respective control. Data is presented as mean ± SD, n=3. * represents p < 0.05; ** represents p < 0.01; *** represents p < 0.001.

Next, we investigated whether knock-down of the miR-1246 target genes would copy its pro-inflammatory phenotype in MSCs. Accordingly, *PRKAR1A* or *PPP2CB* were knocked-down in MSCs to investigate their impact on *RELA* transcription and p65 protein expression (see Supplementary Figure 6 for knock-down efficiencies). Knock-down of *PPP2CB* significantly increased *RELA* expression, whereas si*PRKAR1A* had no effect on *RELA* transcript levels (Figure 5A). At protein level, knock-down of either *PRKAR1A* or *PPP2CB* led to a significant up-regulation of total p65 in MSCs whereas phosphorylation of p65 at Ser536 remained unaffected (Figure 5B, Supplementary Figure 7A). Based on these findings, we investigated if knock-down of *PRKAR1A* or *PPP2CB* would also lead to increased releases of IL-6, CCL2, and CCL5. Indeed, knock-down of *PRKAR1A* significantly increased transcriptions and releases of IL-6 and CCL2 (Figure 5C, Supplementary Figure 7B), whereas knock-down of *PPP2CB* significantly decreased releases of IL-6 and CCL2 (Figure 5C). Neither of the knock-downs increased release of CCL5 in MSCs to a detectable level. As miR-1246 targets PRKAR1A and PPP2CB at the same time and eventually leads to pro-inflammatory responses in MSCs, we included a simultaneous double knock-down of *PRKAR1A* and *PPP2CB*. In MSCs, the combinatorial knock-down of both miR-1246 target genes increased releases of IL-6 and CCL2 to the level of *PRKAR1A* knock-down alone (Figure 5C).

Interestingly, knock-down of *PRKAR1A* did not elevate phosphorylation of p65 at S536 in MSCs, even though we showed that miR-1246 mediates its pro-inflammatory phenotype *via* NF-κB. Therefore, we investigated whether PRKAR1A leads to transcription of inflammatory mediators *via* p65. Accordingly, we knocked down *PRKAR1A* in combination with *RELA* in MSCs and quantified IL-6, CCL2 and CCL5 in the CM. Indeed, combinatorial knock-down significantly decreased the secretion of IL6 and CCL2, which was induced by knock-down of *PRKAR1A* in combination with siCtrl (Supplementary Figure 7C). At last, combinatorial knock-down of *PRKAR1A* with *RELA* did also not affect releases of CCL5 (Supplementary Figure 7C).

We hypothesized that downregulation of PRKAR1A *via* knock-down or direct targeting by miR-1246 would lead to increases in PKA activities. Hence, miR-1246 or si*PRKAR1A* was transfected into MSCs and activities of PKA were quantified subsequently. Indeed, miR-1246 significantly increased PKA activities by ~30% and knock-down of *PRKAR1A* by ~200%, compared to the respective controls (Figure 5D).

### PPP2CB knock-down requires TNFα to act pro-inflammatory in MSCs

**Figure 6:**
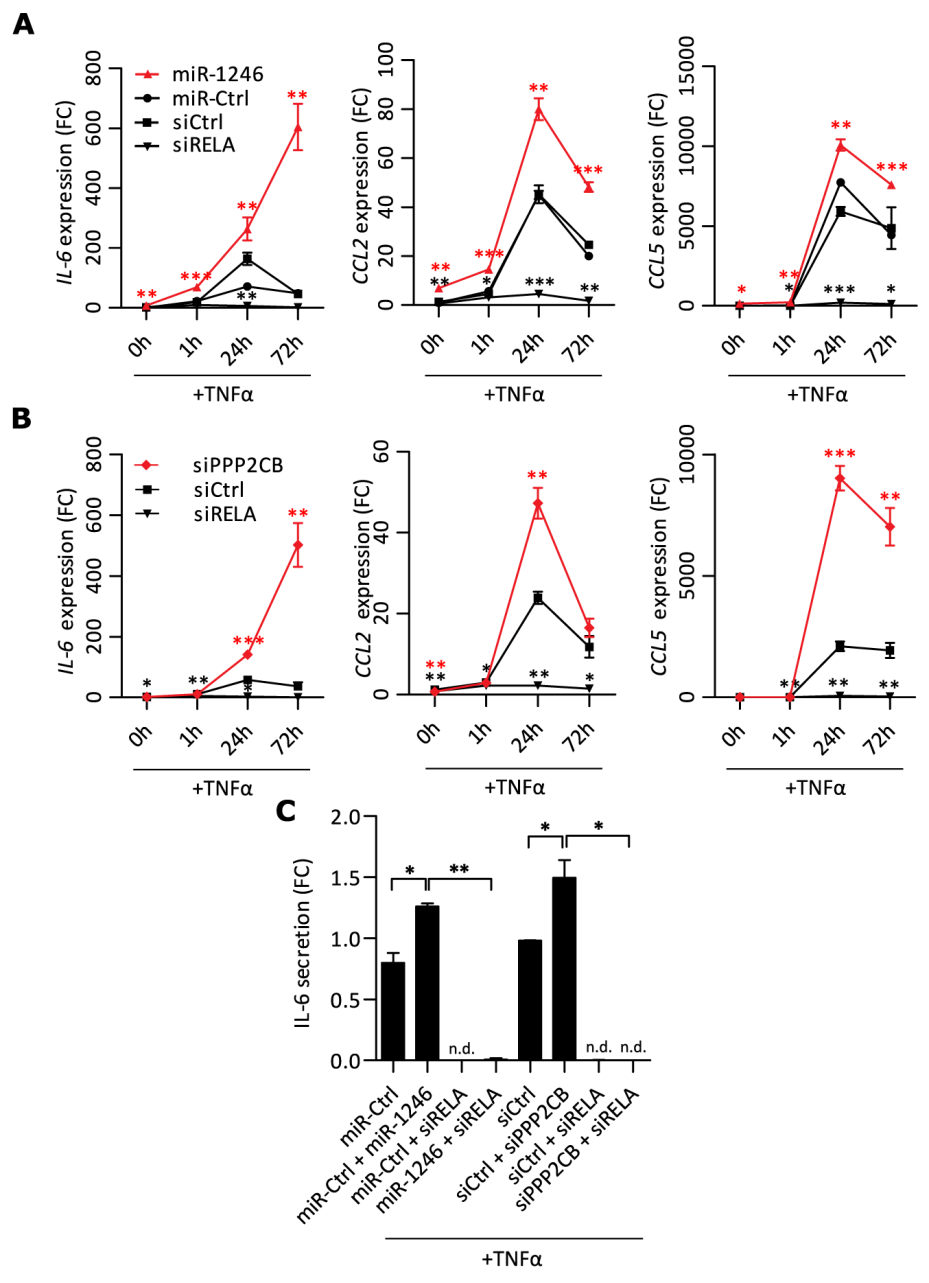
miR-1246 and PPP2CB regulate transcription of pro-inflammatory mediators in combination with TNFα stimulation **A.** mRNA expression analysis after miRNA or siRNA transfections in MSCs. Cells were stimulated with TNFα (20ng/ml) for different time periods and mRNA expression was analyzed by qRT-PCR. Data is presented as mean ± SD, n=3. Red asterisks indicate significance comparing miR-1246 to miR-Ctrl, black asterisks comparing si*RELA* to siCtrl. **B.** mRNA expressions quantified by qRT-PCR after gene specific knock-downs in MSCs with additional TNFα (20ng/ml) stimulation for different time periods. Data is presented as mean ± SD, n=3. Red asterisks indicate statistical analysis of si*PPP2CB* compared with siCtrl and black asterisks for si*RELA* compared to siCtrl. **C.** Quantification of MSC released IL-6 by FLISA in CM after gene specific knock-downs or miR-1246 overexpression with additional TNFα (20ng/ml) stimulation of 24h. Data is presented as mean ± SD, n=2, n.d. = not detectable. * represents p < 0.05; ** represents p < 0.01; *** represents p < 0.001.

We showed that knock-down of *PPP2CB* significantly increased *RELA* and total p65 levels, but did not increase transcription of IL-6, CCL2 or CCL5. Based on this finding, we hypothesized that an additional inflammatory stimulus like TNFα is needed to trigger p65 translocation into the nucleus and to thereby enhance NF-κB transcriptional responses. Nevertheless, as we revealed that PPP2CB is a direct target of miR-1246, we first investigated if miR-1246 would also increase the transcription of IL-6, CCL2 and CCL5 under inflammatory conditions. To this end, we stimulated MSCs with TNFα after miR-1246 overexpression and analyzed the kinetics of *IL-6, CCL2* and *CCL5* mRNA expressions in response to NF-κB activation. 48h after transfection, MSCs were stimulated with TNFα for different time periods and mRNA expressions were quantified (Figure 6A). Knock-down of *RELA* in combination with TNFα stimulation led to a complete block of TNFα-induced *IL-6*, *CCL2* and *CCL5* transcriptions compared to siRNA control. Transcriptional peaks were observed for all three transcripts at 24h, followed by decreases in expression at 72h after miR-Ctrl or siCtrl transfections in combination with TNFα, suggesting transcriptional negative feedback loops. Strikingly, miR-1246 overexpression released the apparent negative feedback on *IL-6* transcription since the levels of *IL-6* continued to strongly increase also at 72h of TNFα stimulation (Figure 6A). Overall, miR-1246 transfected MSCs expressed *IL-6*, *CCL2* and *CCL5* significantly higher at all time points compared to the control transfections. Based on these findings, we investigated if the negative feedback on *IL-6* transcription is mediated *via* PPP2CB. To this end, we knocked-down *PPP2CB* in MSCs and stimulated the cells with TNFα for different time periods. Indeed, knock-down of *PPP2CB* in combination with TNFα stimulation phenocopied the miR-1246-enhanced transcriptional response of IL-6 in combination with TNFα. Knock-down of *PPP2CB* released the negative feedback on IL-6 transcription and continuously increased *IL-6* over time, with a delayed onset at the 24h time point (Figure 6B). Further, knock-down of *PPP2CB* in combination with TNFα stimulation also significantly increased *CCL2* and *CCL5* mRNA expressions at 24h and *CCL5* mRNA levels at 72h.

Both *PPP2CB* knock-down and miR-1246 overexpression continuously increased *IL-6* mRNA levels over time. Therefore, we quantified released IL-6 in the CM after either *PPP2CB* knock-down or miR-1246 overexpression in combination with 24h of TNFα stimulation in MSCs. Both treatments significantly increased the TNFα induced release of IL-6 (Figure 6C). A combinatorial knockdown of *RELA* with each of those conditions completely blocked this effect, demonstrating, that the impact of miR-1246 or *PPP2CB* knock-down on TNFα-induced releases of IL-6 was dependent on p65 (Figure 6C). We conclude that PP2A is not only involved in regulation of p65 abundance, it could also act as important phosphatase shutting NF-κB signaling off, once it got activated in MSCs.

### Functional impact of miR-1246 perturbed MSCs on cells of the TME

**Figure 7:**
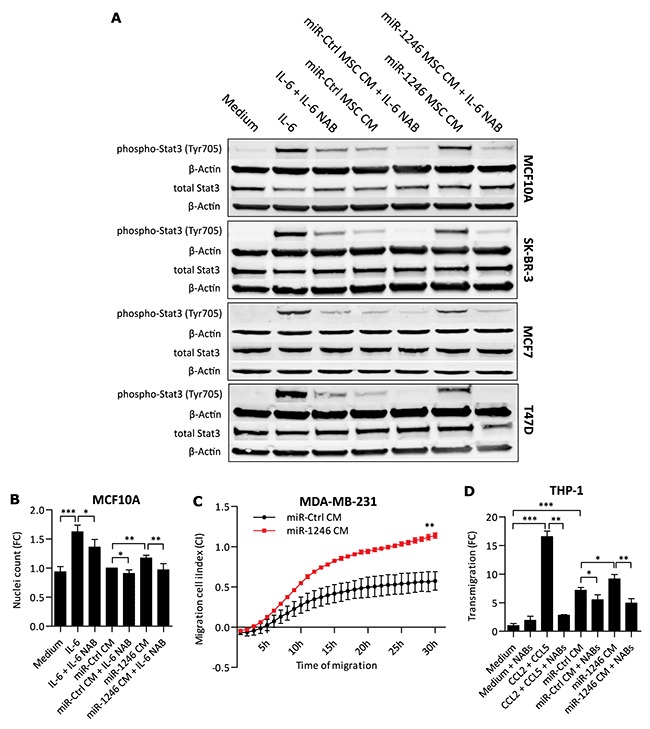
Functional effects of MSC CM on breast cancer cells **A.** Western blot analysis of epithelial and breast cancer cell lines. Cells were stimulated for 20min with CM after miRNA overexpression in MSCs. β-Actin was used as loading control. IL-6 was used as positive control for Stat3 phosphorylation at Tyrosine 705 (Tyr705) and IL-6 NAB to neutralize IL-6. Blots are representatives of n=3 for each cell line. **B.** Nuclei count of MCF10A cells stimulated with miR-1246 transfected MSC CM (miR-1246 CM). IL-6 was used as positive control for induction of MCF10A proliferation and IL-6 NAB antibody to neutralize IL-6 in the medium or CM under all conditions. The data was normalized to cell counts after stimulation with miR-Ctrl overexpressing MSC CM (miR-Ctrl CM) and is presented as mean ± SD, n=4. **C.** Migration of MDA-MB-231 cells after pre-conditioning for 48h with CM of MSCs overexpressing miR-Ctrl (miR-Ctrl CM) or miR-1246 (miR-1246 CM). Migration towards complete growth medium was measured using a RTCA over a period of 30h. Data for each time point is presented as mean ± SD, n=3. Significance was calculated using Wilcoxon-Mann-Whitney-Test. **D.** CM medium of MSCs after miRNA transfection was used for THP-1 cell attraction. A combination of CCL2 NAB + CCL5 NAB (NABs) was used to neutralize the recruiting effect of THP-1 cell attraction. The data is presented as mean ± SD, n=3. * represents p < 0.05; ** represents p < 0.01; *** represents p < 0.001.

Based on our findings that miR-1246 induced releases of key inflammatory mediators, we followed up on their functional effects in other cells of the TME. We investigated if increased IL-6 levels in CM of MSCs after miR-1246 overexpression would lead to increased Stat3 phosphorylations in Jak-Stat-negative breast cancer and epithelial cell lines [73]. To this end, we stimulated the ER- breast cancer cell line SK-BR-3, ER+ breast cancer cell lines MCF7 and T47D and the epithelial breast cell line MCF10A with CM of miR-1246 overexpressing MSCs. Indeed, CM of miR-1246 overexpressing MSCs induced Jak-Stat signaling at significant higher levels compared to CM of miR-Ctrl transfected MSCs (Figure 7A). The induction of Stat3 phosphorylation at Tyrosine705 (Tyr705) could be blocked with IL-6 NAB. Next, we investigated if rh-IL-6 could induce proliferation of MCF10A cells, a model cell line of Jak-Stat signaling activation [73]. MCF10A cells were stimulated with CM retrieved 24h after miR-1246 transfection in MSCs (Supplementary Figure 8) and we measured significant increases in MCF10A proliferation, which could be blocked with IL-6 NAB (Figure 7B). Further, pre-conditioning of ER- MDA-MB-231 cells with CM of miR-1246 overexpressing MSCs significantly increased cell migration compared to control treatments (Figure 7C). This effect could be reduced significantly with CCL2 or CCL5 NABs (Supplementary Figure 9). At last, we investigated if CM of miR-1246 overexpressing MSCs could recruit also other cell types contributing to an overall inflammatory environment. To this end, we tested whether monocytes were attracted by CM of miR-1246 overexpressing MSCs, compared to CM of miR-Ctrl-transfected MSCs. Indeed, monocyte recruitment was increased by CM of miR-1246 overexpressing MSCs (Figure 7D). Both rh-CCL2 and rh-CCL5 recruited THP-1 cells when used individually (data not shown), and a combination of CCL2 and CCL5 NABs was used to block monocyte recruitment under all conditions (Figure 7D).

## DISCUSSION

In this study, we identified miR-1246 as potential novel oncomiR in breast cancer by analyzing a comprehensive miRNA expression dataset with long term clinical follow-up derived from breast cancer patients [70, 72]. miR-1246 has recently gained interest as diagnostic and prognostic marker in several tumor entities including breast cancer [74–79]. At the functional level miR-1246 has been shown to promote proliferation, invasion and migration of cervical or hepatocellular carcinoma cells [80, 81], as well as metastasis formation of non-small cell lung cancer *in vivo* [82]. However, it has been shown that circulating miR-1246 gets predominantly released by cancer cells and that it is rather retained by mesenchymal cells [83]. This retaining of miR-1246 within cells of the tumor stroma underlines its biological importance within these cells.

**Figure 8:**
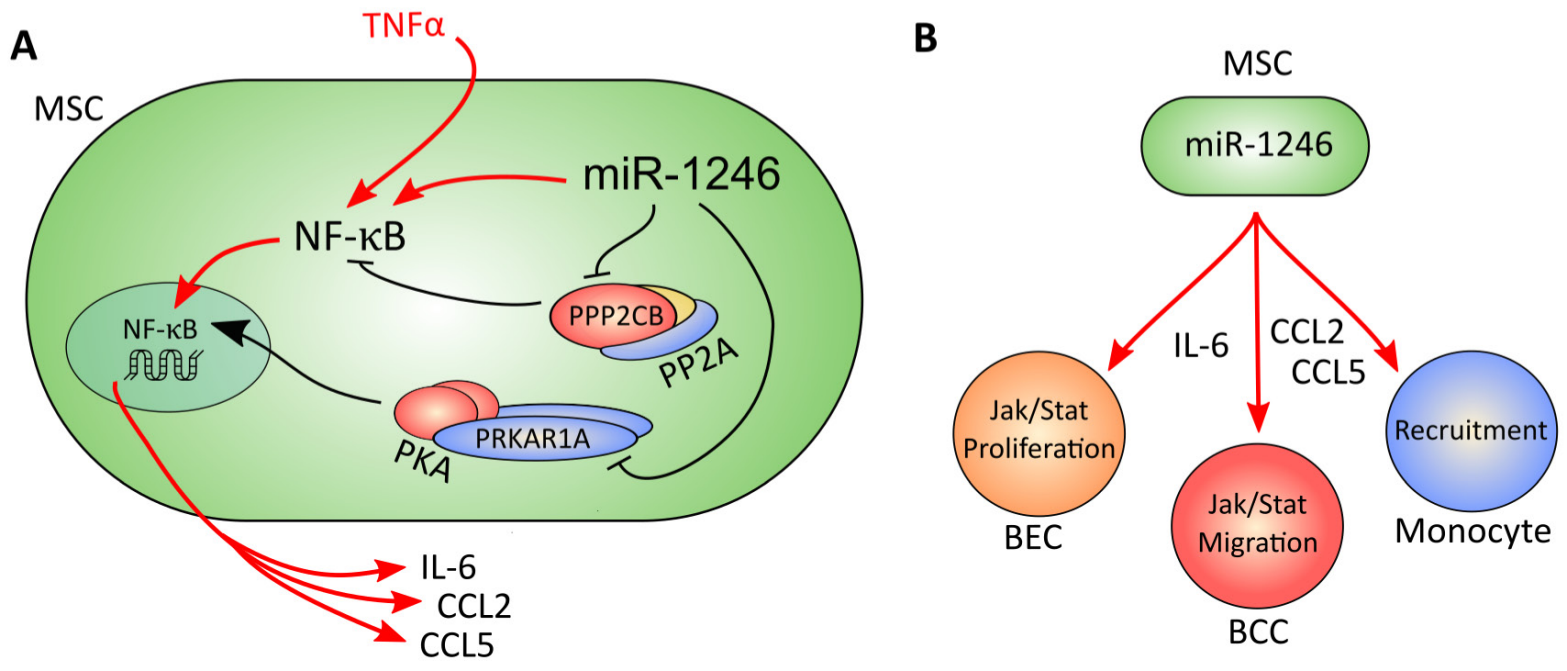
Schematic overview of the molecular function of miR-1246 in MSCs **A.** miR-1246 directly targets the catalytic subunit PPP2CB of PP2A and the regulatory subunit PRKAR1A of PKA. By this, it manipulates the function of both protein complexes and leads to p65 (NF-κB)-mediated activities in MSCs. It acts independent of TNFα and leads to transcription of the pro-inflammatory cytokines and chemokines IL-6, CCL2 and CCL5. **B.** Pro-inflammatory mediators secreted by MSCs impact on the activities of cells types within the TME. miR-1246 expressing MSCs secrete high levels of IL-6, CCL2 and CCL5. IL-6 induces Jak-Stat signaling in breast epithelial cells (BEC) and breast cancer cells (BCC), and enhances proliferation of BECs. CCL2 and CCL5 induces migration of BCCs and recruits monocytes.

Prior functional studies of miR-1246 focused on its expression and function in cancer cells only and neglected its potential roles in other cell types of the TME. Here, we demonstrate for the first time by miRNA sequencing that miR-1246 is expressed in MSCs. We show that transcription of miR-1246 in MSCs is regulated by CM of ER- breast cancer cells and reveal that miR-1246 enhances NF-κB signaling independently of TNFα. miR-1246 elevates cytoplasmic and nuclear levels of total and phospho-p65 in MSCs. It further increases transcription of the key inflammatory mediators IL-6, CCL2 and CCL5 in a p65-dependent manner. Most striking, miR-1246 overexpression in combination with TNFα stimulation leads to continuous increases in *IL-6* mRNA levels in MSCs over time. In summary, we show for the first time that miR-1246 elevates pro-inflammatory responses in BM-derived MSCs (schematic overview in Figure 8A).

miR-1246 mediated transcription and release of inflammatory cytokines and chemokines results in direct functional effects in different cell types of the TME (schematic overview in Figure 8B). We demonstrate that CM of miR-1246 transfected MSCs induces Jak-Stat signaling in breast cancer and epithelial cells, as well as proliferation of epithelial cells. The same CM effectively increases migration of MDA-MB-231 cells and thereby affects cancer cell motility as key event in tumor progression. Further, it is well known that recruitment of monocytes, neutrophils and TAMs is crucial to promote an inflammatory environment [43, 44] and that MSCs activated with TNFα can promote this effect [84]. In this context, we show that CM of MSCs recruits monocytes and that CM of miR-1246 transfected MSCs increases this effect. By this, we shed light on miR-1246 as key mediator of an inflammatory response in tumor-associated MSCs with impact on different hallmarks of cancer in neighboring cells.

PRKAR1A has been described as tumor-suppressor in several cancer entities [48], and ablation of PRKAR1A has recently been shown to induce mammary neoplasia [85]. We demonstrate that miR-1246 leads to increased PKA activity by directly targeting PRKAR1A. PKA has been linked to activation of NF-κB by directly phosphorylating the nuclear factor NF-Kappa-B p65 subunit (p65) [86, 87] or enhancing p65 transcriptional activity [88], eventually leading to increased expression of IL-6 [89]. In MSCs, PKA activity has thus far only been shown to promote cell differentiation [90, 91]. We demonstrate that knock-down of the PKA regulatory subunit *PRKAR1A* increases p65 levels and upregulates transcription and release of IL-6 as well as CCL2 in MSCs, without an additional inflammatory stimulus. By this, we are the first to define PKA as enhancer of inflammation in MSCs even in a non-inflammatory environment (Supplementary Figure 10). However, knock-down of *PRKAR1A* did not affect phosphorylation of p65 at S536 and we hypothesize that miR-1246 leads to further activating phosphorylations of p65. These might address other Serine residues than S536 [92] and could in context of targeting *PRKAR1A* involve S276 of p65 [87, 93].

However, miR-1246 promotes overall pro-inflammatory responses in MSCs, also in combination with TNFα. We show that miR-1246 additionally targets PP2As catalytic subunit PPP2CB to achieve this effect. PP2A is deregulated in several tumors (including breast cancer) and widely described as a tumor-suppressor [55, 94]. It has been shown that blocking of PP2A activities increases NF-κB translocation into the nucleus and upregulates NF-κB-mediated transcriptional activities [95–97]. It has been shown that PP2A directly interacts with and dephosphorylates p65 at basal levels and prevents NF-κB translocation to the nucleus in melanoma cells [98]. However, even though downregulation of its catalytic subunit PPP2CB increases p65 levels, knock-down of *PPP2CB* without further TNFα stimulation does not elevate pro-inflammatory mediators in MSCs. In this context, it has been shown, that inhibition of PP2A needs an additional TNFα stimulus to upregulate releases of IL-8 and IL-6 [99]. Our data supports this finding by demonstrating that knock-down of *PPP2CB* in MSCs in combination with TNFα stimulation significantly increases the transcriptional response of all three inflammatory mediators IL-6, CCL2 and CCL5. We assume that PP2A shuts off NF-κB signaling after being activated with TNFα. In this context, we show that both miR-1246 and knock-down of *PPP2CB* release a negative feedback mechanism of IL-6 transcription after TNFα stimulation. We conclude that PP2A's catalytic subunit PPP2CB takes over a negative regulatory role of pro-inflammatory responses in MSCs under inflammatory conditions. By directly targeting PPP2CB, miR-1246 achieves an overall pro-inflammatory phenotype also in an inflammatory environment (Supplementary Figure 10).

We reveal that miR-1246 directly targets at least two genes to increase pro-inflammatory responses in MSCs. It is important to gain more knowledge about the interactions of both posttranscriptional modifiers PKA and PP2A to understand their hierarchical connection and function in context of miR-1246-mediated pro-inflammatory responses in MSCs. Both PKA and PP2A have been widely accepted as antagonistic regulators of many molecular processes [100–102]. In the context of inflammation, PKA has been shown to directly phosphorylate PPP2CB, leading to degradation and inactivation of PP2A and thereby resulting in increased NF-κB activities [89]. Contrarily, PKA has also been described to activate PP2A by directly phosphorylating PPP2R5D, one of the regulatory PP2A subunits [103]. In MSCs, miR-1246 acts pro-inflammatory in a TNFα-independent manner by targeting PRKAR1A and PPP2CB at the same time. Nevertheless, the functional connection of both protein complexes in MSCs has not been described until now. We approached this topic by combinatorial knock-down of *PRKAR1A* and *PPP2CB* in MSCs and detected increases in releases of IL-6 and CCL2 at similar extents to knock-down of *PRKAR1A* alone. This indicates that PKA activities dominate the functions of PP2A during regulation of pro-inflammatory signaling pathways in MSCs under non-inflammatory conditions. We suggest complex interactions between PKA and PP2A that lead to gene specific regulations of pro-inflammatory responses in MSCs, which are strongly depending on inflammatory signals of the environment. However, neither knock-down of *PRKAR1A*, nor of *PPP2CB-induced* *CCL5* transcription in absence of TNFα stimulation. Therefore, miR-1246 likely targets a yet unidentified gene, leading to upregulation of CCL5 under non-inflammatory conditions and thereby contributing to a complex system of miR-1246 mediated NF-κB modifications.

## CONCLUSION

The aim of this study was to unravel novel miRNA-mediated mechanisms in cells of the TME in the context of breast cancer. We demonstrate that miR-1246 is highly expressed in MSCs and show that it promotes pro-inflammatory responses *via* PKA and PP2A by directly targeting their subunits PRKAR1A and PPP2CB. The miR-1246-induced secretion of pro-inflammatory cytokines and chemokines IL-6, CCL2 and CCL5 in MSCs is mediated *via* NF-κB, but independent of TNFα. We suggest a high level of complexity of miR-1246-mediated NF-κB signaling in MSCs, resulting in direct functional impact on different cell types of the TME. By this, miR-1246 forms a key player in MSC-triggered inflammation in the context of breast cancer and should be evaluated for *in-vivo* studies to test its potential as therapeutic target.

## MATERIALs AND METHODS

### Cell culture

Primary human bone marrow (BM)-derived MSCs from healthy individuals were purchased from Lonza, grown in MSCGM™ (Lonza GmbH, Cologne, Germany) and used until passage 6 (donor information in Supplemental Materials). Starvation medium was MSCGM™ with all supplements except serum. Cell lines MCF7, T47D, SK-BR-3, MDA-MB-231, MDA-MB-468, MCF10A, THP-1 BT474, HCC1143, HCC1937, HCC1954, CAMA1, ZR-75-30, BT549 were obtained from ATCC (LGC Standards GmbH, Wesel, Germany), HEK293-FT from Invitrogen AG (Invitrogen AG, Carlsbad, USA) (Growth media in Supplemental Materials). All cells lines were authenticated by Multiplexion (Heidelberg, Germany) and negatively tested for mycoplasma contamination.

Recombinant human (rh)-TNFα (PeproTech, RH, USA) and rh-IL-6 (R&D Systems, Wiesbaden-Nordenstadt, Germany) were used at final concentrations of 20ng/ml, rh-CCL2 and rh-CCL5 (R&D Systems, Wiesbaden-Nordenstadt, Germany) at 50ng/ml. IL-6 and CCL5 neutralizing antibodies (NAB) (R&D Systems, Wiesbaden-Nordenstadt, Germany) were used at final concentrations of 2µg/ml, CCL2 NAB at 4.5µg/ml. Protein neutralization was performed for 3h on ice.

Transfections were performed with Lipofectamine® 2000 (Invitrogen AG, Carlsbad, USA) according to manufacturer's instructions. miRNAs and siRNAs were used at final concentrations of 30nM (Details in Supplementary Table 5).

### Conditioned medium (CM) and quantification of secreted proteins

MSCs were transfected for 48h, starved overnight (o.n.), and grown in starvation medium for 72h if not indicated differently. Breast cancer or epithelial cells were grown for 72h in complete growth medium. Subsequently, CM was retrieved, cell debris was removed by centrifugation and stored immediately at -80°C and thawed only once for experimental usage. For ultracentrifugation (UC), the CM was first centrifuged for 30min at 2,000g to remove dead cells and cell debris. The residual supernatant was centrifuged at 100,000g for 180min using a SW 41 Ti Rotor in a Beckman L8-70M Ultracentrifuge (Beckman Coulter, California, USA). Separation of proteins larger than 4kDa in CM of cancer cells was performed with AMICON® Ultra-4 filtration units (Merck Millipore, Darmstadt, Germany) at 4000g for 30 minutes.

MSC CM was analyzed for secreted proteins using Human Cytokine Array Kit, Panel A (R&D Systems, Wiesbaden-Nordenstadt, Germany) according to manufacturer's instructions except that IRDye®800CM Streptavidin (LI-COR, Lincoln, NE, USA) was used in a 1:4000 dilution in PBS for visualization. Membranes were scanned with Odyssey Reader and analyzed with Odyssey 2.1 (LI-COR, Lincoln, NE, USA). Fluorescence linked-immunosorbent assay (FLISA) was used for protein quantification in CM (Protocol in Supplemental Materials).

### RNA isolation and analysis

For mRNA or miRNA expression analysis, MSCs were transfected for 48h unless indicated otherwise. Total-RNA was isolated using miRNeasy Mini kit (Qiagen, Hilden, Germany). mRNA was quantified using TaqMan® based qRT-PCR with UPL probes (Roche Diagnostics GmbH, Mannheim, Germany) and miRNA was quantified with TaqMan® MicroRNA Assays (Thermo Fisher Scientific, Massachusetts, USA) (Protocol in Supplemental Materials, primer sequences in Supplementary Table 6).

Genome wide gene expression analysis was performed on HumanHT-12 v4 BeadChips (Illumina, San Diego, CA, USA) by the microarray unit of the DKFZ Genomics and Proteomics Core Facility (GPCF). Raw data of miR-1246 overexpression in MSCs of two donors was uploaded to ArrayExpress (https://www.ebi.ac.uk/arrayexpress/experiments/E-MTAB-5033/) with the accession E-MTAB-5033. For miRNA sequencing, MSCs were grown to 90% confluency, starved o.n. and harvested at time points 0h and 24h in duplicates. Total RNA was isolated using miRNeasy Mini kit (Qiagen, Hilden, Germany). RNA quantity and quality checks, library preparation and miRNA sequencing were performed by the sequencing unit of the DKFZ GPCF (Details in Supplemental Materials).

### Immunoblotting

Protein analysis was performed with Western blot after 72h of miRNA or siRNA transfection, unless indicated differently (Details in Supplemental Materials; Primary antibodies in Supplementary Table 7). Secondary IRDye®680 or IRDye®800-conjugated antibodies (LI-COR, Lincoln, NE, USA) were used for band visualization. Membranes were scanned and analyzed with Odyssey scanner and Odyssey 2.1, respectively (LI-COR, Lincoln, NE, USA). For quantification, local background subtraction and β-Actin normalization was performed.

### Functional and luciferase reporter assays, 3’UTR cloning

Kinase activity was quantified using PKA Kinase-Activity Assay Kits (Abcam, Cambridge, Great Britain) according to manufacturer's instructions. See Supplemental Materials for protocols on NF-κB and 3’UTR reporter assays and cloning (primer sequences in Supplementary Table 8), as well as functional assays on MCF10A proliferation, MDA-MB-231 migration and THP-1 chemo-attraction assays.

### Dataset and statistical analysis, and visualization

Survival analysis was performed for mRNAs and miRNAs (excluding putative miRNAs) using the METABRIC dataset [70, 72]. Kaplan-Meier analyses was applied to generate survival curves and log-rank test was applied to evaluate the significance of group differences in survival rates (details in Supplemental Materials). mRNA or miRNA expression analysis was performed with a combination of all patients of the METABRIC discovery and validation datasets [72], with available mRNA or miRNA data of the specific gene.

If not mentioned differently, experimental statistical analysis was performed with two-tailed Student's t-test and equality of variance was tested with the F-Test. Graphs were created using GraphPad Prism 5 and graphical illustrations using Inkscape v. 0.91.

## SUPPLEMENTARY MATERIALS FIGURES AND TABLES




