# Supplementary material for: miRNA-1246 induces pro-inflammatory responses in mesenchymal stem/stromal cells by regulating PKA and PP2A
Source: Oncotarget. 2017 Jan 31;8(27):43897–914. doi: 10.18632/oncotarget.14915 (PMC5546423; doi:10.18632/oncotarget.14915)
Supplement: Supplementary file 1 [file oncotarget-08-43897-s001.pdf]

# miRNA-1246 induces pro-inflammatory responses in mesenchymal stem/stromal cells by regulating PKA and PP2A

## SUPPLEMENTAL DATA

### Cell culture (Supplemental)

#### Detailed MSC donor information

MSC donor lots (purchased from Lonza GmbH, Cologne, Germany) were:

- #0000423370 (23y, female); #0000296577 (45y, male); #0000305526 (22y, female)

All MSCs were retrieved from healthy individuals and were purchased from LONZA Group AG and certified to be CD105+, CD166+, CD44+, CD90+ and CD73+ (>=90%) and CD15-, CD34-, CD45-, HLA-DR- and CD19- (<= 10%). Further, MSCs were certified to differentiate into adipocytes, chondrocytes and osteoblasts by LONZA Group AG. We negatively tested MSCs of all donors for mycoplasma and they were certified to be free of HIV, HBV, HCV, mycoplasma contamination by LONZA Group AG. As we did not re-evaluate trilineage differentiation potential at used passages, we clearly refer to mesenchymal stem/stromal cells by using the abbreviation MSC.

### Growth media for cell lines and growth conditions

Cells were cultured in media and reagents from Gibco BRL (Gran Island, NY, USA) if not mentioned different. All cells were grown in Cell Culture Flasks (75cm$^2$ or 175cm$^2$, red filter cap) obtained from Greiner BioOne (Greiner Bio-One International GmbH, Kremsmünster, Austria).

**BT474** complete growth medium: DMEM medium supplemented with 10% FBS, 1% Penicillin/Streptomycin sulfate (Pen/Strep) and 1% Glutamine.

**BT549** complete growth medium: RPMI-1640 medium supplemented with 10% FCS, 1% Pen/Strep, 1% Glutamine, 0,01mg/ml bovine insulin (Sigma-Aldrich, Saint-Louis, USA).

**CAMA1** complete growth medium: DMEM medium supplemented with 10% FBS, 1% nonessential amino acids (NEAA), 1% Pen/Strep and 1% Glutamine.

**HCC1143, HCC1937, HCC1954** complete growth media: RPMI-1640 medium supplemented with 10% FCS, 1% Pen/Strep, 1% Glutamine.

**HEK293-FT** complete growth medium: DMEM medium supplemented with 10% FBS, 1% NEAA, 1% Geneticin, 1% Pen/Strep.

**MCF-7** full growth medium: MEM medium supplemented with 10% FBS, 1% non-essential amino acids, 0,01mg/ml bovine insulin (Sigma-Aldrich, Saint-Louis, USA), 1% Pen/Strep. Starvation medium: like complete growth medium, just with 1% FBS.

**MCF-10A** complete growth medium: DMEM F12 medium supplemented with 5% Horse Serum, 20ng/ml EGF (BD Biosciences, CA, USA), 0,5% µg/ml Hydrocortisone (Sigma-Aldrich, Saint-Louis, USA), 100ng/ml Choleratoxin (Sigma-Aldrich, Saint-Louis, USA), 1% Pen/Strep. Starvation medium: like complete growth medium, just with 1% FBS.

**MDA-MB-231** complete growth medium: Leibovitz's L-15 medium supplemented with 10% FBS, 1% L-glutamine, 1% nonessential amino acids, 1% Pen/Strep. Starvation medium: like complete growth medium, just with 1% FBS.

**MDA-MB-468** and **ZR-75-30** complete growth medium: RPMI-1640 medium supplemented with 10% FBS, 1% Pen/Strep.

**SK-BR-3** complete growth medium: McCoy's 5A medium supplemented with 10% FCS, 1% Pen/Strep and 1% Glutamine. Starvation medium: like complete growth medium, just with 1% FBS.

**T47D** complete growth medium: RPMI-1640 medium supplemented with 10% FBS, 1% Pen/Strep and NEAA. Starvation medium: like complete growth medium, just with 1% FBS.

**THP-1** complete growth medium: RPMI-1640 medium supplemented with 10% FCS, 1% Pen/Strep, 1% Glutamine. Starvation medium: like complete growth medium, just with 1% FBS.

### Fluorescence linked-immunosorbent assay (FLISA)

96-well plates were coated with monoclonal antibodies purchased from R&D (R&D Systems, Wiesbaden-Nordenstadt, Germany), followed by sample and detection antibody incubation. Black µCLEAR® 96-well plates (Greiner Bio-One International GmbH, Kremsmünster, Austria) were coated o.n. with IL-6, CCL2 or CCL5 monoclonal antibodies (R&D Systems, Wiesbaden-Nordenstadt, Germany) at final concentrations of 4µg/ml in PBS at 4°. Three washing steps with wash buffer (0,05% Tween20 in PBS) were performed. Plates were blocked with blocking buffer (1% Bovine Serum Albumin Fraction V (BSA) in PBS) (Sigma-Aldrich, Saint-Louis, USA) in PBS for 2h at RT. Three washing steps with wash buffer were performed. Samples were diluted 1:2 with blocking buffer and cell specific starvation medium was used as blank. A protein standard using recombinant human proteins for IL-6, CCL2 or CCL5

was prepared in blocking buffer and diluted samples were applied for 2h at RT on the plate. Three washing steps with wash buffer were performed. Biotinylated detection antibodies were purchased from R&D Systems, Inc. (R&D Systems, Wiesbaden-Nordenstadt, Germany) and incubated for 2h at RT at final concentrations of 2µg/ml. Three washing steps with wash buffer were performed. Streptavidin, AlexaFluor® 680 conjugate (Thermo Fisher Scientific Inc) was dilutes 1:5000 in wash buffer and applied for 1h in the dark at RT. Two washing steps with wash buffer and one final washing step with deionized water were performed. Plates were dried via upside-down centrifugation, scanned and analyzed with Odyssey Reader or Odyssey 2.1, respectively (LI-COR, Lincoln, NE, USA). For analysis blank subtraction was performed.

## RNA isolation and analyses (Supplemental)

### Quantitative real time-PCR (qRT-PCR)

mRNA and miRNA were isolated using miRNeasy Mini kit (Qiagen, Hilden, Germany) according to manufacturer's instructions. cDNA synthesis was performed with Revert Aid H Minus 1st Strand cDNA Synthesis Kit (Thermo Fisher Scientific, Massachusetts, USA) and qRT-PCR amplifications of specific genes were performed in an ABI Prism 7900HT Sequence Detection System (Applied Biosystems). Universal Probe Library (UPL) (Roche Diagnostics GmbH, Mannheim, Germany) probes were used to increase primer specificity (gene and UPL primer sequences in Supplementary Table 6). Analysis was performed by using $2^{-\Delta\Delta CT}$. Data was normalization to the average CT of housekeeping genes *GAPDH* and *HPRT*.

miRNA quantification was performed with TaqMan® MicroRNA Assays for hsa-mir-1246, RNU44 and RNU48 (Thermo Fisher Scientific, Massachusetts, USA). Analysis was performed by using $2^{-\Delta\Delta CT}$ and miR-1246 expression was normalized to the average CT of housekeepers RNU44 and RNU48.

### mRNA expression array

DKFZ microarray core facility performed sample preparation, RNA quality control and hybridization. Normalization was performed with Illumina-lumi pipeline and differential expression analysis with Chipster v1.4.6 (http://chipster.sourceforge.net). Accession of miR-1246 overexpressed in MSCs of two different donors can be performed at ArrayExpress:

Donor #1 and #2: https://www.ebi.ac.uk/arrayexpress/experiments/E-MTAB-5033/

### miRNA Sequencing

RNA Quality check, quantification, library preparation and sequencing were performed by the sequencing unit of the DKFZ Genomics and Proteomics Core Facility. Quality check was performed with Agilent Bioanalyzer 2100 with the RNA 6000 Nano Kit (Agilent Technologies, Santa Clara, USA) and quantification with Qubit® fluorometer (Invitrogen, Carlsbad, USA). Library preparation was performed with NEBNext® Multiplex Small RNA Library Prep Set for Illumina® according to manufacturer's instruction. Libraries were normalized, pooled and clustered on the cBot (Illumina, San Diego, CA, USA) with a final concentration of 10 pM (Illumina, San Diego, CA, USA) using TruSeq SR Cluster Kit v3. Sequencing on HiSeq 2000 (Illumina) was performed using TruSeq SBS Kit v3 (Illumina, San Diego, CA, USA) according to the manufacturer's instructions. 6 samples were pooled per lane.

Data processing and analysis: Following quality filtering and adaptor trimming, reads with less than 15 bases were removed. Filtered reads were aligned with Bowtie[1] to the human mature miRNA sequences of miRBase release v.21 (www.mirbase.org), allowing for one mismatch. Pipelines implemented in the HUSAR system[2] were used. Read per million (RPM) were calculated by dividing reads of mature miRNAs of one sample by the sum of all reads of the same sample.

## Immunoblot (Supplemental)

Cells were lysed on ice with MPER lysis buffer (Thermo Fisher Scientific, Massachusetts, USA) containing protease inhibitor Complete Mini and anti-phosphatase PhosSTOP ordered from Roche Diagnostics. Protein amount was quantified with Pierce™ BCA Protein Assay Kit (Thermo Fisher Scientific, Massachusetts, USA) according to manufacturer's instruction. Protein isolates were denatured using 4x Roti Load (Roth-Chemie GmbH) for 5min at 95°C and 20µg of total protein was separated on 12,5% SDS PAGE for each sample. A PVDF membrane Immobilon-P was used for blotting (Merck, Darmstadt, Germany). Membranes were blocked for 1h at RT and primary antibodies (see Supplementary Table 7) were incubated o.n. at 4°C in 5% BSA in TBS-T. After 3 washing steps with TBST, the secondary IRDye®680 or IRDye®800 conjugated antibodies (LI-COR, Lincoln, NE, USA) were diluted 1:10000 in TBS-T and incubated for 45 min at RT. Membranes were scanned and analyzed with Odyssey scanner and Odyssey 2.1, respectively (LI-COR, Lincoln, NE, USA). For Western blot quantification, local background subtraction and β-Actin normalization was performed.

## Luciferase reporter assay and 3'UTR cloning

HEK293-FT cells were transfected with miR-Ctrl or miR-1246 in combination with a NF-κB luciferase plasmid (Stratagene, La Jolla, CA, USA) (100ng/well) and pMIR-REPORT ß-Gal vector (20ng/well) (Ambion, Austin, TX, USA) in 96-well plates. After 48h, cells were starved o.n. and stimulated with TNFα for 5h. Luciferase activity was measured using a luminometer infinite M200

(Tecan Group AG, Männedorf, Switzerland). ß-Gal activity was quantified with beta-glo® Luminescent Assay Kit (Promega, Madison, USA) and used for normalization.

3'UTRs of the target genes were cloned into a psiCHECK2 vector (Promega, Madison, USA) according to manufacturer's instructions. As the binding site of miR-1246 in PRKAR1A 3'UTR was in close proximity to the open reading frame (ORF) (distance of ~35nt) the cloned 3'UTR fragment of PRKAR1A contained part of the ORF (length of cloned ORF fragment: 65nt) due to technical reasons in the primer design. miR-1246 was predicted to have one binding site within this fragment, which impacted on luciferase signal. As this binding site was in the ORF and biologically not relevant, it was mutated using QuikChange Lightning Site-Directed Mutagenesis Kit (Agilent Technologies) according to manufacturer's instructions for control conditions (See in Supplementary Table 8B). The 3'UTR carrying plasmids were co-transfected with miR-1246 or miR-Ctrl into MCF-7 cells for 48h and renilla and firefly luciferase signals were quantified with a luminometer infinite M200 (Tecan Group AG, Männedorf, Switzerland). MCF-7 cells served as model cell line for this assay as MSCs did not survive plasmid transfections. Predicted binding sites in the 3'UTRs were mutated using QuikChange Lightning Site-Directed Mutagenesis Kit (Agilent Technologies) to prove direct targeting by miR-1246 (primer sequences in Supplementary Table 8).

## Functional assays

### Proliferation of MCF10A cells

Different growth periods after 48h of miR-1246 overexpression in MSCs led to different extends of IL-6 secretion (Supplementary Figure 9). As CM retrieved after 72h of culture in starvation medium increased MCF10A proliferation to the maximum of the dynamic measurable range, CM for this assay was retrieved already after 24h.

3500 MCF10A cells/well were seeded in 96 wells plates (Microplate 96 well, Black with LID Greiner Bio-One International GmbH, Kremsmünster, Austria) and starved for 6h the following day. In samples were prepared on ice. IL-6 neutralizing antibody was incubated with MSC conditioned medium for 2hrs on ice. Starved MCF10A cells were stimulated for 72 hours with 50% MSC CM and 50% MCF10A starvation medium and specific cytokine or NAB supplements. Cells were washed with PBS, fixed for 4min with 90% ice cold Methanol, washed with PBS, stained with 1:1000 diluted DAPI (Sigma-Aldrich, Saint-Louis, USA) for 10min in the dark at RT. PBS was added and they were scanned at the microscope molecular devices IXM XLS (Molecular Devices, Sunnyvale CA, USA) and nuclei were counted with Molecular Devices Software.

### Migration and cell attraction

Used was an xCELLigence RTCA DP (RTCA, Roche Diagnostics GmbH, Mannheim, Germany). MDA-MB-231 cells were seeded 3x10^5 in CELLSTAR® 6-Well plates (Greiner Bio-One International GmbH, Kremsmünster, Austria). The next day, cells were stimulated with 1ml of 50% MSC CM and 50% MDA-MB-231 starvation medium for 48h. Stimulations with recombinant human cytokines or neutralizing antibodies were performed in 50% MSC starvation medium and 50% MDA-MB-231 starvation medium. After the incubation time cells reseeded at 70000 cells / well to upper well of CIM-16 Plate in a xCELLigence RTCA DP (Roche Diagnostics GmbH, Mannheim, Germany) in cell specific starvation medium (100µl). The lower chamber was filled with 175µl of MDA-MB-231 complete growth medium as chemoattractant. Cell Index was calculated every 15 minutes for the given period.

### Monocyte recruitment

THP-1 cell attraction was performed using a trans-well system with 5.0µm pore size from Corning® Costar® (Sigma-Aldrich, Saint-Louis, USA). NABs, recombinant human proteins, CM or combinations of all were incubated on ice for 3h and applied into the lower chamber of the trans-well system as chemoattractant (600µl). 2*10^5 cells were seeded into the upper well in starvation medium and cells migrated for 24h. Then, the fluid of the lower chamber was collected in FACS tubes and centrifuged for 10min at 1600rpm. The supernatant was discarded and the pellet resuspended in 150µl PBS. Migrated cells were counted for 1min using a FACS Calibur (BD Biosciences, CA, USA).

## METABRIC dataset analysis (Supplemental)

It was discriminated between high (4$^{th}$ quartile) and low (1$^{st}$ quartile) expression in the METABRIC dataset. For Kaplan Meier analysis patients were set as: groups with "1" for "disease specific death" and "0" for "alive", other patients were excluded in the analysis. Survival analysis was limited to 10years of survival.

mRNA data was retrieved from: https://www.synapse.org/#!Synapse:syn1688369/wiki/27311

miRNA data was retrieved from: https://ega-archive.org/datasets/EGAD00010000438

## REFERENCES

1. Langmead B, Trapnell C, Pop M, Salzberg SL. Ultrafast and memory-efficient alignment of short DNA sequences to the human genome. Genome Biol. 2009; 10 R25.
2. Ernst P, Glatting KH, Suhai S. A task framework for the web interface W2H. Bioinformatics. 2003; 19: 278–82.

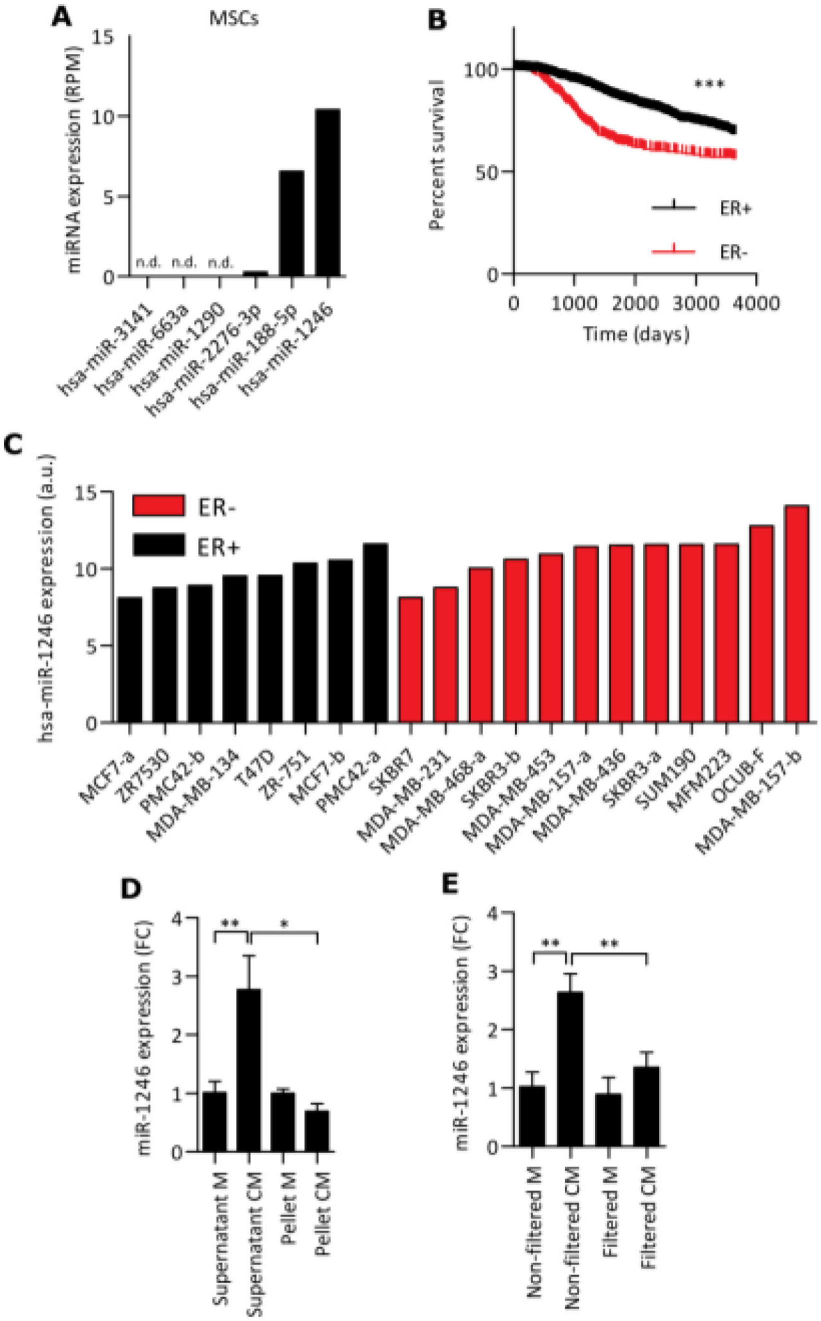


**Supplementary Figure 1: A.** miRNA-sequencing of human BM-derived MSCs. MSCs were grown to 90% confluency and harvested at time points 0h and 24h, in duplicates each. Reads per million (RPM) of miRNAs were calculated and the data is presented as median of n=4. **B.** Overall breast cancer survival analysis of ER+ (n=1174) vs. ER- (n=380) patients from the METABRIC dataset. **C.** hsa-miR-1246 expression analysis in breast cancer cell lines, retrieved from and annotated according to the METABRIC dataset. Statistical analysis of the groups ER- vs. ER+ cell lines was performed and presented in Figure 1C. **D.** MSCs were stimulated for 14h with growth medium (M) or conditioned medium (CM) of MDA-MB-468 cells and miR-1246 expression was measured by qRT-PCR. CM of MDA-MB-468 cells was centrifuged at different forces (g). If ultracentrifuged (100,000g), MSCs were either stimulated with the “supernatant” (containing proteins) or the resuspended “pellet” (containing exosomes and microvesicles). **E.** MSCs were stimulated for 14h with growth medium (M) or conditioned medium (CM) of MDA-MB-468 cells and miR-1246 expression was measured by qRT-PCR. If the CM was “filtered” with Amicon Ultra-4 filtration units at 4000g, MSCs were stimulated with the flow through. Control conditions for filtration were “non-filtered” but centrifuged samples, containing the whole protein fraction. * represents $p < 0.05$; ** represents $p < 0.01$; *** represents $p < 0.001$.

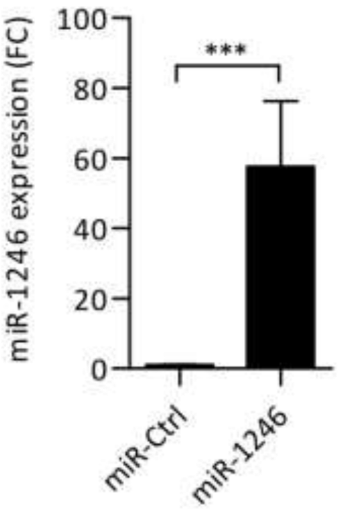


**Supplementary Figure 2: Overexpression of miR-1246 in MSCs.** Quantification of mature miR-1246 transcript 48h after transfection was performed with qRT-PCR in MSCs and normalized to a miR-Ctrl transfection. *** represents $p < 0.001$.

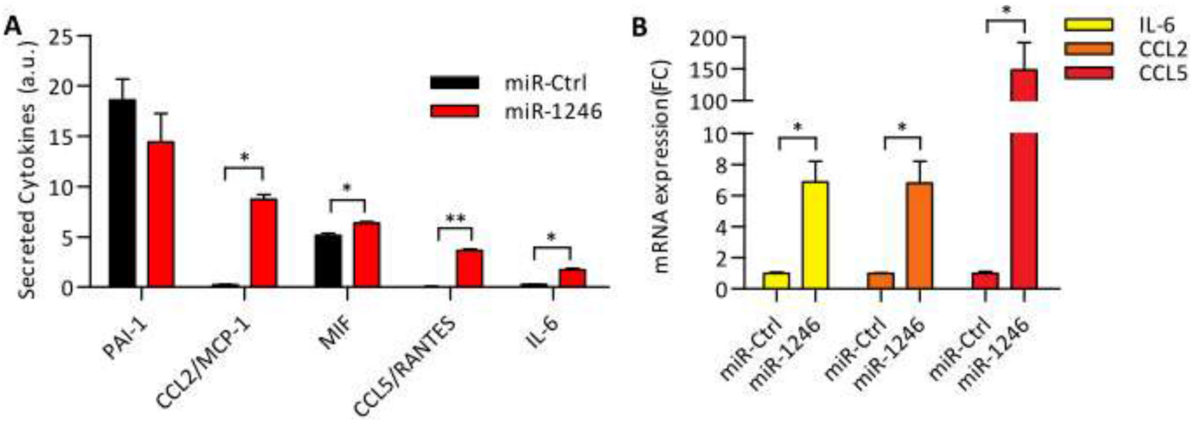


**Supplementary Figure 3: A.** Human cytokine array analysis of MSC CM after miR-Ctrl or miR-1246 over-expression with n=2 and presented as mean ± SD for each protein. **B.** qRT-PCR analysis of inflammatory mediator mRNAs 48h after miRNA overexpressions. Data is presented as mean ± SD, n=3. * represents $p < 0.05$; ** represents $p < 0.01$.

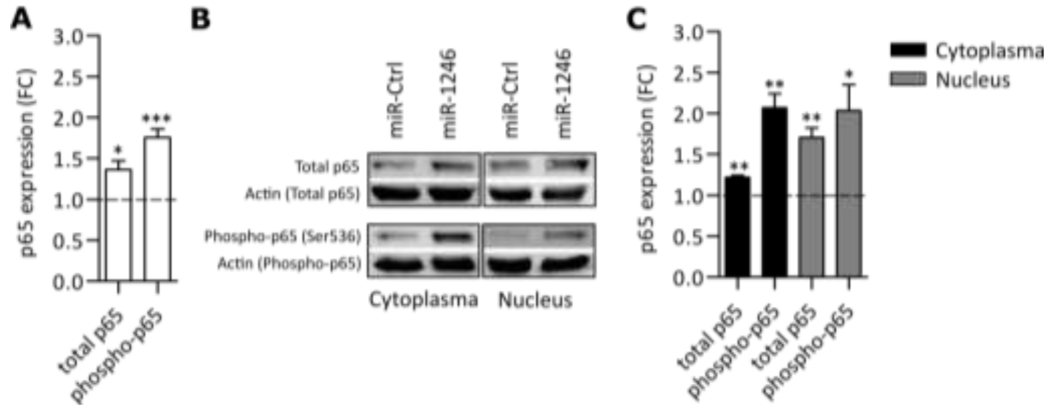


**Supplementary Figure 4: A.** miR-1246 overexpression in MSCs and quantification of p65 in Western blot data with n=6, representing MSCs of two donors. miR-1246 was transfected for 72h, without further TNFα stimulation. Data is shown as fold change compared to the respective miR-Ctrl transfection (dashed line). **B.** Representative Western blots of nuclear or cytoplasmic protein extracts of MSCs after miRNA perturbations. Analyzed were total or phospho-p65 in MSCs after miR-1246 or miR-Ctrl overexpressions. The shown Western blots are representative blots of n=4. **C.** Quantifications of B) with n=4. Shown are miR-1246 induced changes in total or phospho-p65 expressions. Data was normalized to β-Actin and compared to miR-Ctrl overexpressions (dashed line) in MSCs for each condition. Data is presented as FC. * represents $p < 0.05$; ** represents $p < 0.01$; *** represents $p < 0.001$.

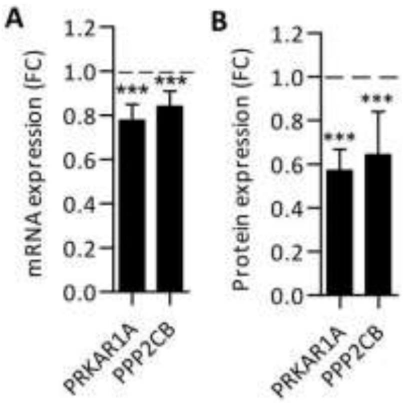


**Supplementary Figure 5: A.** mRNA analysis by qRT-PCR after miR-1246 overexpression in MSCs. Data was normalized and compared to miR-Ctrl overexpression in MSCs (dashed line). The data represents overexpression in MSCs of 3 different donors in triplicates each, n=9. **B.** Protein expression quantification 72h after miR-1246 or miR-Ctrl overexpression in MSCs of 3 different donors, with n=3 for each individual. Quantifications of Western blot data with n=9 was performed comparted to miR-Ctrl overexpression (dashed line) after background subtraction and normalization to β-Actin. *** represents $p < 0.001$.

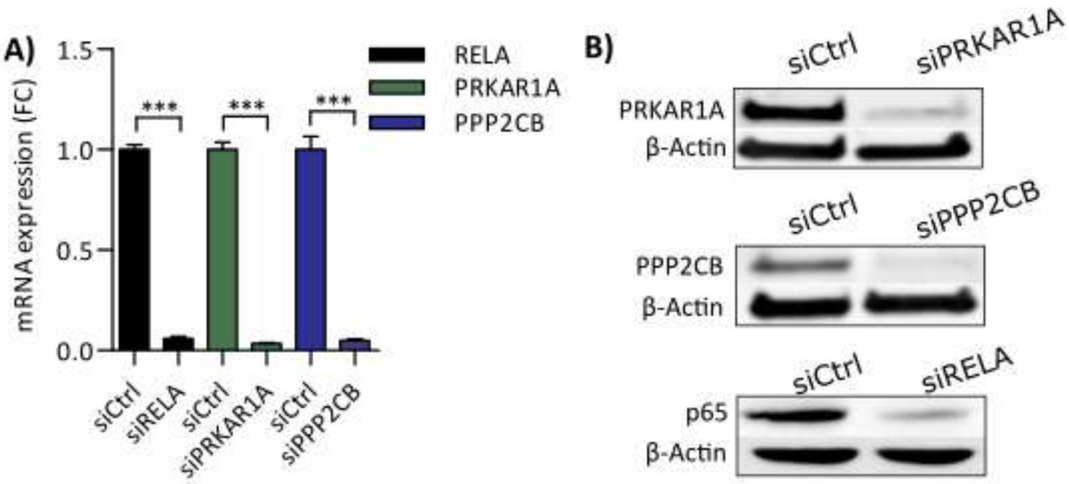


**Supplementary Figure 6: A.** *RELA, PRKAR1A* and *PPP2CB* mRNA expression analysis after siRNA induced knock-downs in MSCs. MSCs were transfected for 48h and each indicated condition was compared to siCtrl with n=3. *** represents $p < 0.001$. **B.** Western blot analysis after siRNA-based gene specific knock-downs. β-Actin was used as loading control, each blot is a representative of n=3.

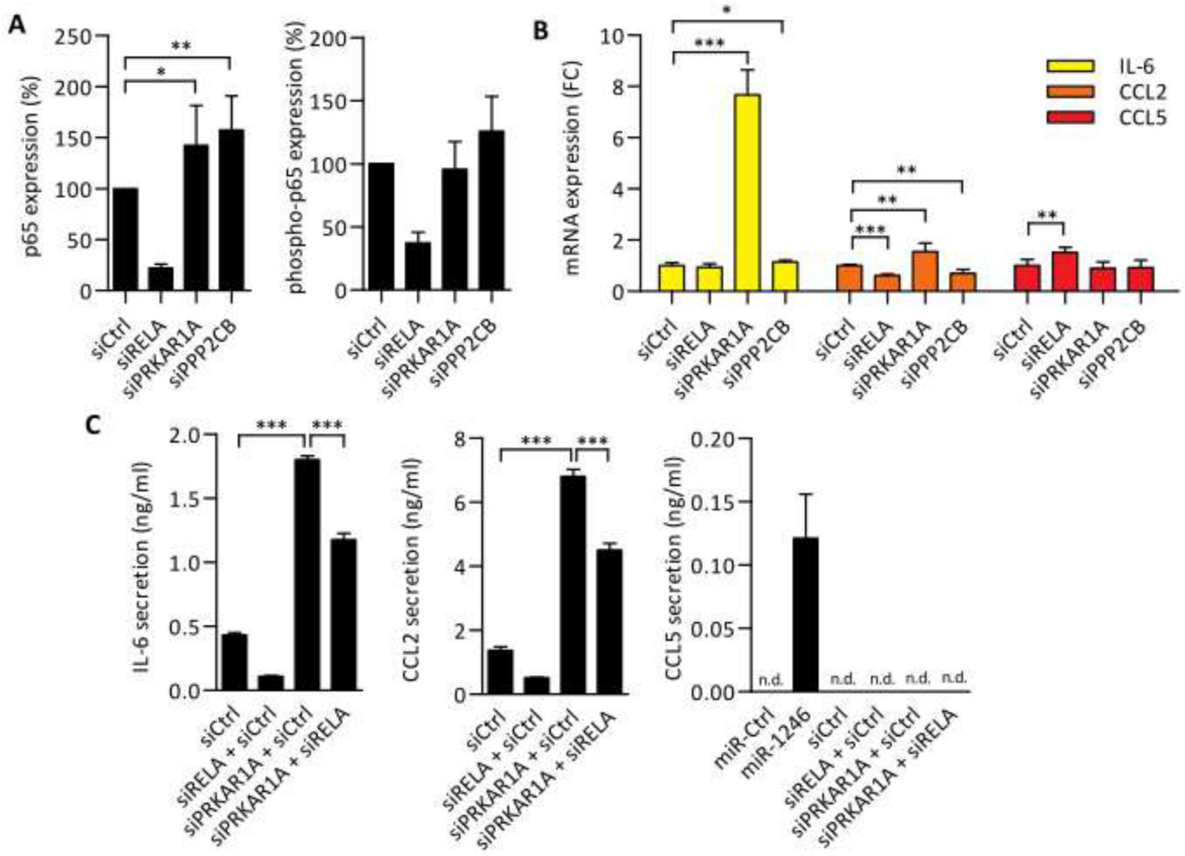


**Supplementary Figure 7: A.** Total and phospho-p65 (Ser536) protein expression analysis after knock-down of indicated genes in MSCs. Quantification of n=6 Western blots representing data of two different MSC donors. Data was normalized to siCtrl and is presented as mean ± SD. **B.** mRNA analysis by qRT-PCR after gene specific knock-downs for 48h in MSCs with n=6. Data was normalized and compared to siCtrl. **C.** Protein secretion of MSCs after combinatorial mRNA knock-downs in MSCs. Secreted proteins were quantified with FLISA and miR-1246 was used as positive control for CCL5 secretion. Data is presented as mean ± SD with n=3. n.d.: not detectable. * represents $p < 0.05$; ** represents $p < 0.01$; *** represents $p < 0.001$.

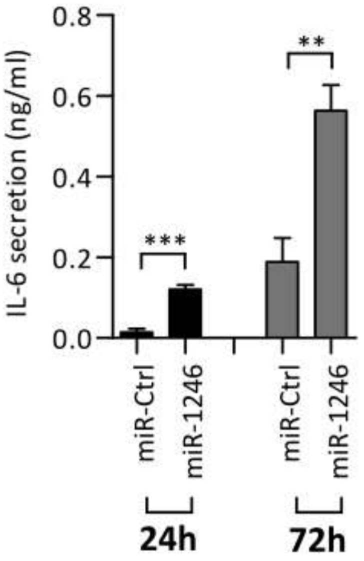


**Supplementary Figure 8: IL-6 secretion after 24h or 72h of miRNA overexpressions in MSCs.** Secreted IL-6 was quantified with FLISA and the data is presented as mean ± SD, n=3. ** represents $p < 0.01$; *** represents $p < 0.001$.

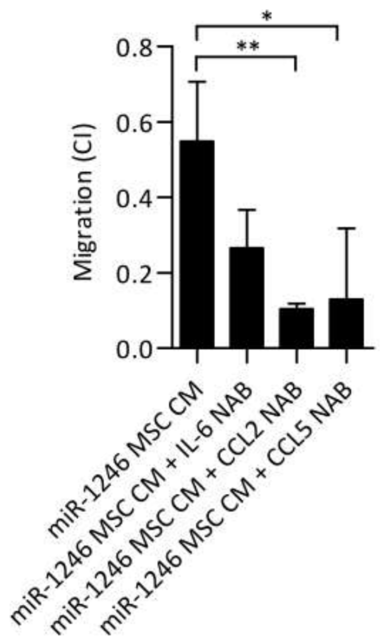


**Supplementary Figure 9: Migration of MDA-MB-231 cells after 48h of pre-conditioning with CM of miR-1246 overexpressing MSCs without or in combination with neutralizing antibodies (NAB) against the indicated cytokine/ chemokine.** Data was retrieved as cell index (CI) by RTCA after 30h of migration and is presented as mean ± SD with n=3. * represents $p < 0.05$; ** represents $p < 0.01$.

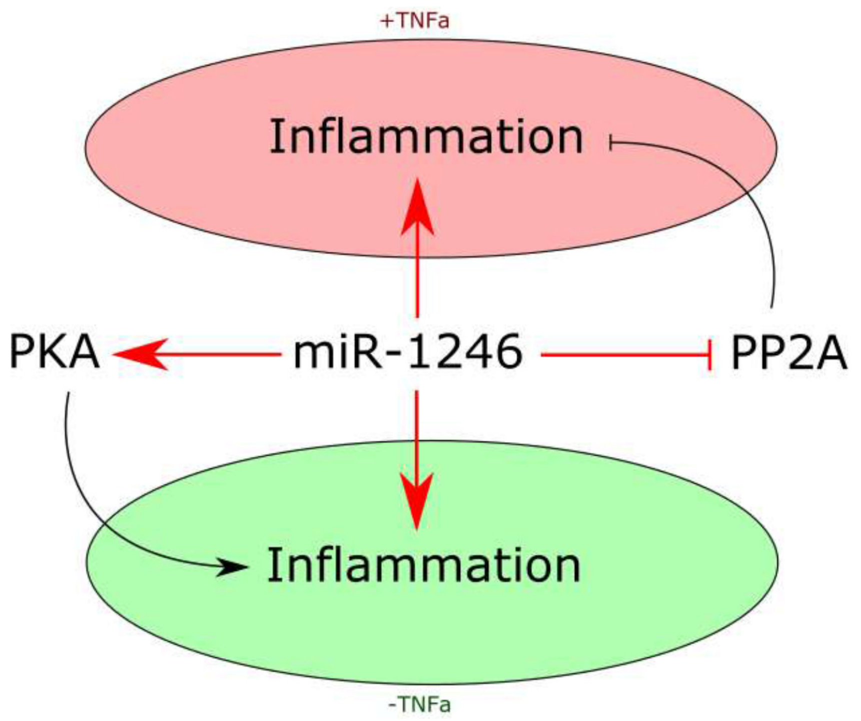


**Supplementary Figure 10: Schematic overview of miR-1246, promoting pro-inflammatory responses in MSCs in a TNFα-independent manner.** miR-1246 targets *PRKAR1A* and thereby increases PKA activity to induce pro-inflammatory responses in a non-inflammatory environment (-TNFα). Further, miR-1246 increases the TNFα-mediated induction of pro-inflammatory responses (+TNFα) by directly targeting PP2As catalytic subunit *PPP2CB* in MSCs.

**Supplementary Table 1: miRNA correlation analysis in breast cancer.** Survival analyses of miRNAs of the METABRIC dataset. Candidate selection was performed by setting the cut-off to p < 1.0e-6. hsa-miR-29b-1* was excluded for further progressing as high expression of hsa-miR-29b-1* correlated with better overall breast cancer patient survival.

See Supplementary File 1

**Supplementary Table 2: miRNA sequencing and expression in MSCs.** miRNA-sequencing of human BM-derived MSCs. MSCs were grown to 90% confluency and harvested at time points 0h and 24h, each in duplicates. Reads per million (RPM) of miRNAs were calculated and the data is presented as median of n=4. Reads were mapped to miRBase v.21 (mirbase.org).

See Supplementary File 1

**Supplementary Table 3: Protein secretion after miRNA overexpression in MSCs.** Proteome Profiler cytokine array analysis of MSC CM after miR-Ctrl or miR-1246 overexpression. The data is presented in arbitrary units (a.u.) after background subtraction. A protein was determined to be expressed when expression levels were > 0.5 (a.u.).

See Supplementary File 1

**Supplementary Table 4: Predicted and downregulated target genes of miR-1246.** List of potential target genes of miR-1246 after gene array-based differential expression analysis. Listed genes were significantly downregulated (p<0.01 and reduction of >20%) after miR-1246 overexpression in MSCs of two different donors compared to miR-Ctrl. In addition, all genes were predicted targets of miR-1246 (TargetScan.org). Data is presented as mean with n=3 for each condition in each individual.

See Supplementary File 1

**Supplementary Table 5: miRNAs and siRNAs**

See Supplementary File 1

**Supplementary Table 6: qRT-PCR primers, sequences and UPL probe**

| Gene | Primer direction (fw=forward; rv=reverse) | Identifier | Sequence (5' -> 3') | UPL Probe |
|---|---|---|---|---|
| CCL5 | CCL5_fw | NM_002985.2 | tgcccacatcaaggagtattt | #59 |
| | CCL5_rv | | ctttcgggtgacaaagacg | #59 |
| CCL2 | CCL2_fw | NM_002982.3 | agtctctgccgcccttct | #40 |
| | CCL2_rv | | gtgactggggcattgattg | #40 |
| IL-6 | IL6_fw | NM_000600.3 | gatgagtacaaaagtcctgatcca | #40 |
| | IL6_rv | | ctgcagccactggttctgt | #40 |
| GAPDH | GAPDH_fw | NM_002046.3 | agccacatcgctcagacac | #60 |
| | GAPDH_rv | | gcccaatacgaccaaatcc | #60 |
| HPRT | HPRT1_fw | NM_000194.2 | tgaccttgatttattttgcatacc | #73 |
| | HPRT1_rv | | cgagcaagacgttcagtcct | #73 |
| PRKAR1A | PRKAR1A_fw | NM_002734 | tgatcaaggagagacggatgt | #12 |
| | PRKAR1A_rv | | tcaaagcaagttctccaaagc | #12 |
| PPP2CB | PPP2CB_fw | NM_001009552.1 | ccacttacagctttagtagatggaca | #16 |
| | PPP2CB_rv | | tccagtgtgtctatggatggag | #16 |
| RELA | RELA_fw | NM_001145138.1 | cgggatggcttctatgagg | #48 |
| | RELA_rv | | ctccaggtcccgcttctt | #48 |

**Supplementary Table 7: Antibodies and dilutions**

| Protein | Product ID (company) | Dilution (in 5% BSA in TBS-T) |
|---|---|---|
| beta-Actin | Anti-Actin (clone C4) (MP Biomedicals) | 1:10000 |
| phospho-p65 | CST3033 (Cell Signaling) | 1:1000 |
| phospho-Stat3 | CST9131 (Cell Signaling) | 1:1000 |
| PPP2CB | ab168371 (Abcam) | 1:1000 |
| PRKAR1A | CST5675 (New England Biolabs) | 1:1000 |
| total p65 | SC-372 (Santa Cruz) | 1:250 |
| total Stat3 | CST4904 (Cell Signaling) | 1:2000 |

**Supplementary Table 8: Cloning and mutagenesis primer sequences.** **A.** List of primer sequences used for cloning of 3’UTRs into psiCHECK2 vector. Directions are given as forward (FW) and reverse (RV). **B.** List of primers used for mutagenesis of miR-1246 binding sites in *PRKAR1A* and *PPP2CB* 3’UTRs and *PRKAR1A* open reading frame (ORF). The cloned fragment of the *PRKAR1A* 3’UTR contained a small part of the *PRKAR1A* ORF due to close proximity of the predicted miR-1246 binding site in the *PRKAR1A* 3’UTR to the ORF. The cloned ORF fragment carried a potential binding site for miR-1246 without biological relevance and was mutated for control conditions (detailed information in supplemental materials).

See Supplementary File 1
