# Supplementary material for: miRNA-1246 induces pro-inflammatory responses in mesenchymal stem/stromal cells by regulating PKA and PP2A
Source: Oncotarget. 2017 Jan 31;8(27):43897–914. doi: 10.18632/oncotarget.14915 (PMC5546423; doi:10.18632/oncotarget.14915)
Supplement: Supplementary file 2 [file oncotarget-08-43897-s002.docx]

**Supplementary Table 1: miRNA correlation analysis in breast cancer.** Survival analyses of miRNAs of the METABRIC dataset. Candidate selection was performed by setting the cut-off to p < 1.0e-6. hsa-miR-29b-1* was excluded for further progressing as high expression of hsa-miR-29b-1* correlated with better overall breast cancer patient survival.

**miRNA**

**P value**

hsa-miR-1290

6.05E-09

hsa-miR-663

1.46E-07

hsa-miR-188-5p

2.16E-07

hsa-miR-2276

4.14E-07

hsa-miR-29b-1*

4.41E-07

hsa-miR-1246

6.99E-07

hsa-miR-3141

9.80E-07

hsa-miR-195

1.52E-06

hsa-miR-409-5p

1.76E-06

hsa-miR-101

2.29E-06

hsa-miR-30a*

2.46E-06

hsa-miR-135a*

2.89E-06

hsa-miR-30a*

3.30E-06

hsa-miR-30c-2*

3.83E-06

hsa-miR-204

4.72E-06

hsa-miR-411

6.37E-06

hsa-miR-4274

7.03E-06

hsa-miR-132

7.07E-06

hsa-miR-487b

7.18E-06

hsa-miR-30e*

7.30E-06

hsa-miR-99a

7.73E-06

hsa-miR-551b

8.85E-06

hsa-miR-513a-5p

1.08E-05

hsa-miR-3138

1.10E-05

hsa-miR-654-3p

1.14E-05

hsa-miR-492

1.74E-05

hsa-miR-99a

2.21E-05

hsa-miR-29b

2.21E-05

hsa-miR-542-5p

2.24E-05

hsa-miR-299-5p

3.35E-05

hsa-miR-4324

3.40E-05

hsa-miR-144*

3.50E-05

hsa-miR-93

3.58E-05

hsa-miR-30c

3.92E-05

hsa-miR-877

3.97E-05

hsa-miR-199b-5p

4.10E-05

hsa-miR-622

4.93E-05

hsa-miR-154

5.83E-05

hsa-miR-483-3p

6.56E-05

hsa-miR-3190-3p

7.33E-05

hsa-miR-151-3p

7.37E-05

hsa-miR-3194

9.66E-05

hsa-miR-379

0.000100449

hsa-miR-4257

0.000105195

hsa-miR-150*

0.00011764

hsa-miR-139-5p

0.000120156

hsa-miR-1287

0.000127144

hsa-miR-125b-2*

0.000136235

hsa-miR-410

0.000136438

hsa-miR-210

0.00015895

hsa-miR-218

0.000159327

hsa-miR-301b

0.000166078

hsa-miR-630

0.000179287

hsa-miR-1185

0.000182088

hsa-miR-335

0.000193762

hsa-miR-24

0.000204039

hsa-miR-106b

0.00020685

hsa-miR-494

0.00021689

hsa-miR-100

0.000221694

hsa-miR-29a

0.000239174

hsa-miR-145*

0.000242854

hsa-miR-765

0.000250033

hsa-miR-539

0.000253087

hsa-let-7b

0.000263837

hsa-miR-485-5p

0.000275717

hsa-miR-602

0.000350666

hsa-miR-373*

0.000375048

hsa-miR-376c

0.000378778

hsa-miR-4271

0.000387785

hsa-miR-3162

0.000400425

hsa-miR-377

0.000422333

hsa-miR-181c

0.000424799

hsa-miR-26b

0.000426386

hsa-miR-4261

0.000445575

hsa-miR-199a-3p

0.000453751

hsa-let-7c

0.000505829

hsa-miR-4314

0.000543456

hsa-miR-4270

0.000598105

hsa-miR-514b-5p

0.00060011

hsa-miR-497

0.000615719

hsa-miR-214*

0.000629961

hsa-miR-557

0.000637206

hsa-miR-3125

0.000664103

hsa-miR-190b

0.000679217

hsa-miR-181c*

0.000704308

hsa-miR-10b

0.000740702

hsa-miR-345

0.000763554

hsa-miR-3147

0.000766542

hsa-miR-574-3p

0.000769011

hsa-miR-200c

0.000809843

hsa-miR-132*

0.000814983

hsa-miR-3196

0.000831094

hsa-miR-196b

0.000943291

hsa-miR-100

0.000943512

hsa-miR-4298

0.000957555

hsa-miR-575

0.001032124

hsa-miR-130b

0.001032438

hsa-miR-4306

0.001036433

hsa-miR-1

0.001053225

hsa-miR-3202

0.00107849

hsa-miR-18b*

0.001142932

hsa-miR-4291

0.001158602

hsa-miR-451

0.001182806

hsa-miR-1271

0.00122601

hsa-miR-10b*

0.001229971

hsa-miR-299-3p

0.001255814

hsa-miR-584

0.001269455

hsa-miR-3132

0.001282923

hsa-miR-338-3p

0.001305616

hsa-miR-140-5p

0.001331828

hsa-miR-337-5p

0.001359498

hsa-miR-152

0.001394261

hsa-miR-125a-3p

0.001409483

hsa-miR-376b

0.001462305

hsa-miR-4320

0.001472718

hsa-miR-1539

0.001725136

hsa-miR-141

0.001763722

hsa-miR-1471

0.00179009

hsa-miR-154*

0.001847996

hsa-miR-1914*

0.001885759

hsa-miR-3154

0.001947961

hsa-miR-381

0.002068968

hsa-miR-1307

0.002226427

hsa-miR-199a-5p

0.002305049

hsa-miR-628-5p

0.002433707

hsa-miR-214

0.002514256

hsa-miR-143

0.002664286

hsa-miR-1202

0.002745498

hsa-miR-1973

0.003014455

hsa-miR-3198

0.003158338

hsa-miR-431*

0.003209631

hsa-miR-34c-5p

0.00335159

hsa-miR-20a*

0.003421612

hsa-miR-369-5p

0.003427148

hsa-miR-3127

0.00342735

hsa-miR-485-3p

0.003444705

hsa-miR-99b

0.003470176

hsa-miR-21*

0.003584296

hsa-miR-329

0.003763281

hsa-miR-625

0.003770165

hsa-miR-665

0.003778278

hsa-miR-4251

0.00390736

hsa-miR-598

0.003934964

hsa-miR-486-5p

0.003942189

hsa-miR-18b

0.004008093

hsa-miR-489

0.004174877

hsa-miR-205*

0.004244561

hsa-miR-3156

0.00427541

hsa-miR-221*

0.004333512

hsa-miR-624*

0.004874908

hsa-miR-542-3p

0.004968691

hsa-miR-499-5p

0.005021321

hsa-miR-376a

0.005050788

hsa-miR-1249

0.005432886

hsa-miR-3131

0.005455238

hsa-miR-371-5p

0.005513203

hsa-miR-223

0.00565288

hsa-miR-15a

0.005869385

hsa-miR-31*

0.006058515

hsa-miR-34b*

0.006202931

hsa-miR-101*

0.006330074

hsa-miR-323-3p

0.006811942

hsa-miR-1182

0.006930328

hsa-miR-1321

0.006938134

hsa-let-7a

0.007007798

hsa-miR-4267

0.007422151

hsa-miR-4327

0.007538357

hsa-miR-342-5p

0.007734537

hsa-miR-409-3p

0.007770073

hsa-miR-500a

0.00780916

hsa-miR-376a*

0.007859831

hsa-miR-127-3p

0.007885962

hsa-miR-10a

0.00802711

hsa-miR-526b

0.008148363

hsa-miR-659

0.00836984

hsa-miR-513b

0.008381708

hsa-miR-1260b

0.00856698

hsa-miR-3174

0.008912844

hsa-miR-516a-5p

0.009104693

hsa-miR-574-5p

0.009501705

hsa-miR-934

0.010029325

hsa-miR-181a

0.010274623

hsa-miR-631

0.010337483

hsa-miR-939

0.010381547

hsa-miR-378b

0.010617373

hsa-miR-202

0.011079375

hsa-miR-450a

0.011838886

hsa-miR-32*

0.012093094

hsa-miR-1288

0.012859396

hsa-miR-662

0.013179941

hsa-miR-222

0.0132986

hsa-miR-337-3p

0.01331191

hsa-let-7g

0.013519298

hsa-miR-136*

0.013576668

hsa-miR-454

0.013712852

hsa-miR-487a

0.014666588

hsa-miR-4286

0.015134092

hsa-miR-296-5p

0.015179826

hsa-miR-493*

0.01540065

hsa-miR-29c

0.016517098

hsa-miR-758

0.018605836

hsa-miR-184

0.018755401

hsa-miR-3065-5p

0.019042776

hsa-miR-98

0.019170629

hsa-let-7f

0.01946612

hsa-miR-744

0.019917511

hsa-miR-133b

0.020202952

hsa-miR-129-5p

0.020611275

hsa-miR-148a

0.020821695

hsa-miR-424

0.020823631

hsa-miR-125b

0.021032491

hsa-miR-146b-5p

0.021117779

hsa-miR-652

0.021241608

hsa-miR-99a*

0.021327835

hsa-miR-374b

0.021345296

hsa-miR-7-1*

0.022963056

hsa-miR-95

0.023695475

hsa-miR-421

0.024057627

hsa-miR-3151

0.025646593

hsa-miR-623

0.025902978

hsa-miR-181a

0.025914548

hsa-miR-181b

0.026207759

hsa-miR-374a

0.026671612

hsa-miR-339-5p

0.026721435

hsa-miR-183*

0.027560276

hsa-miR-149*

0.02830939

hsa-miR-377*

0.029523219

hsa-miR-1301

0.030636248

hsa-miR-28-5p

0.030799218

hsa-miR-510

0.030953565

hsa-miR-219-5p

0.031263187

hsa-miR-328

0.033491245

hsa-miR-636

0.034354346

hsa-miR-153

0.034417312

hsa-miR-3137

0.034714125

hsa-miR-9*

0.034761759

hsa-miR-3200-3p

0.034930191

hsa-miR-1244

0.035133268

hsa-miR-1825

0.035845053

hsa-miR-518e

0.036125764

hsa-miR-493

0.037306951

hsa-miR-483-5p

0.038114226

hsa-miR-3190-5p

0.038638341

hsa-miR-520b

0.039949611

hsa-miR-432

0.040284338

hsa-miR-29c*

0.040961387

hsa-miR-921

0.041224723

hsa-miR-19b

0.042012449

hsa-miR-548d-5p

0.042132928

hsa-miR-545

0.042285791

hsa-miR-422a

0.042657935

hsa-let-7d*

0.042681211

hsa-miR-455-5p

0.042713142

hsa-miR-423-5p

0.04380281

hsa-miR-192

0.044398884

hsa-miR-16

0.044479346

hsa-miR-660

0.045062197

hsa-miR-769-5p

0.045834097

hsa-miR-105

0.048229666

hsa-miR-324-3p

0.049343403

hsa-miR-4328

0.051122118

hsa-miR-431

0.051982526

hsa-miR-365*

0.051998202

hsa-miR-215

0.054162105

hsa-miR-4299

0.056048167

hsa-miR-516b

0.056484402

hsa-miR-1273c

0.058192644

hsa-miR-455-3p

0.05932115

hsa-miR-301a

0.059545856

hsa-miR-1299

0.060331103

hsa-miR-148b

0.061083839

hsa-miR-1267

0.061906413

hsa-miR-645

0.062327847

hsa-miR-30d

0.063323156

hsa-miR-4253

0.063838304

hsa-miR-323-3p

0.064012297

hsa-miR-610

0.065166785

hsa-miR-25*

0.066617594

hsa-miR-1238

0.067466952

hsa-miR-136

0.068862615

hsa-miR-3161

0.070904381

hsa-miR-198

0.071267342

hsa-miR-424*

0.072724962

hsa-miR-601

0.073674323

hsa-miR-31

0.076821047

hsa-miR-4260

0.078886935

hsa-miR-382

0.079364977

hsa-miR-711

0.079410923

hsa-miR-758

0.080396113

hsa-miR-130a

0.082515281

hsa-miR-15b

0.0833712

hsa-miR-1275

0.086711596

hsa-miR-548x

0.087578746

hsa-miR-10a*

0.089947462

hsa-miR-616

0.091709197

hsa-miR-205

0.092670762

hsa-miR-18a

0.097136724

hsa-miR-25

0.100400742

hsa-miR-30b

0.101020933

hsa-miR-96

0.101492678

hsa-miR-30c-1*

0.102384939

hsa-miR-200b*

0.10300371

hsa-miR-566

0.103334962

hsa-miR-512-3p

0.103926242

hsa-miR-641

0.104101587

hsa-miR-193a-5p

0.104642866

hsa-miR-129-3p

0.106655661

hsa-miR-22*

0.106933058

hsa-miR-582-5p

0.107326017

hsa-miR-634

0.107926925

hsa-miR-23a

0.10905156

hsa-miR-583

0.111176817

hsa-miR-502-3p

0.112249761

hsa-miR-196a*

0.113018813

hsa-miR-515-3p

0.113388698

hsa-miR-550a*

0.115985618

hsa-miR-624

0.116166357

hsa-miR-525-5p

0.117932129

hsa-miR-133a

0.121590893

hsa-miR-378

0.125428241

hsa-miR-488*

0.125718492

hsa-miR-24-1*

0.125844815

hsa-miR-518e*

0.12933568

hsa-miR-362-3p

0.130244178

hsa-miR-34a

0.132150063

hsa-miR-370

0.132769087

hsa-miR-181d

0.134852066

hsa-miR-501-5p

0.136721427

hsa-miR-590-5p

0.136934237

hsa-miR-155*

0.137692919

hsa-miR-148a*

0.137904475

hsa-miR-320a

0.140995494

hsa-miR-744*

0.142339932

hsa-miR-936

0.142488333

hsa-miR-122*

0.142776615

hsa-miR-3173

0.143340079

hsa-miR-4312

0.143449753

hsa-miR-515-5p

0.14416374

hsa-miR-433

0.144808246

hsa-miR-187*

0.14509997

hsa-miR-340*

0.146099271

hsa-miR-541

0.146163634

hsa-miR-23a*

0.148569287

hsa-miR-197

0.149362654

hsa-miR-648

0.150201249

hsa-miR-650

0.150606751

hsa-miR-2115*

0.151489074

hsa-miR-495

0.155304092

hsa-miR-488

0.15535558

hsa-miR-100*

0.156421386

hsa-miR-29a*

0.156624032

hsa-miR-21

0.15677373

hsa-miR-17*

0.157221804

hsa-miR-30b*

0.159145809

hsa-miR-137

0.160976975

hsa-let-7i

0.161156581

hsa-miR-134

0.16169015

hsa-miR-320e

0.16405012

hsa-miR-378c

0.164180788

hsa-miR-126*

0.164432133

hsa-miR-150

0.166100465

hsa-miR-1183

0.168520751

hsa-miR-144

0.170750858

hsa-miR-595

0.1709287

hsa-miR-520a-5p

0.172317015

hsa-miR-7

0.174217613

hsa-miR-767-5p

0.174334537

hsa-miR-34a*

0.176267533

hsa-miR-362-5p

0.178071393

hsa-miR-4300

0.178398726

hsa-miR-143*

0.181753346

hsa-miR-15b*

0.181796706

hsa-miR-513c

0.182701737

hsa-miR-550a

0.187060803

hsa-miR-124

0.190418207

hsa-miR-335*

0.192151139

hsa-miR-221

0.194334142

hsa-miR-2114*

0.204390357

hsa-miR-411*

0.204884844

hsa-miR-3177

0.210808613

hsa-miR-212

0.217500948

hsa-miR-522

0.225936715

hsa-miR-29b-2*

0.228582751

hsa-miR-576-3p

0.229099426

hsa-miR-375

0.22948008

hsa-miR-1274a

0.230629259

hsa-miR-145

0.230839703

hsa-miR-1236

0.231213151

hsa-miR-429

0.238394909

hsa-miR-3121

0.240182014

hsa-miR-143*

0.242881548

hsa-miR-708*

0.244582177

hsa-miR-361-5p

0.247574626

hsa-miR-664*

0.248229158

hsa-miR-28-3p

0.248275675

hsa-miR-191*

0.25057493

hsa-miR-543

0.251206098

hsa-miR-183

0.253326385

hsa-miR-34c-3p

0.25568936

hsa-miR-181a*

0.255809186

hsa-miR-3188

0.256511646

hsa-miR-1255b

0.25721443

hsa-miR-1207-3p

0.257468253

hsa-miR-449b

0.26078264

hsa-miR-3186-3p

0.266301655

hsa-miR-19b-1*

0.272278337

hsa-miR-486-3p

0.277665577

hsa-miR-516a-5p

0.279011051

hsa-miR-425*

0.280696472

hsa-miR-449b*

0.28103299

hsa-miR-2115

0.282412008

hsa-miR-93*

0.282782799

hsa-miR-4269

0.283943515

hsa-miR-126

0.284150996

hsa-miR-518b

0.286626281

hsa-miR-628-3p

0.288259864

hsa-miR-933

0.288329524

hsa-let-7i*

0.288753372

hsa-miR-582-3p

0.290846129

hsa-miR-16-2*

0.291712831

hsa-miR-200c*

0.294333951

hsa-miR-770-5p

0.295350051

hsa-miR-769-3p

0.29588401

hsa-miR-331-3p

0.296012259

hsa-miR-147b

0.298019194

hsa-miR-200b

0.298450956

hsa-miR-363

0.300838367

hsa-miR-889

0.300966468

hsa-miR-548q

0.301958403

hsa-miR-30a

0.307737934

hsa-miR-3163

0.307950008

hsa-miR-33b*

0.316796329

hsa-miR-521

0.321074923

hsa-miR-9

0.324359503

hsa-miR-135a

0.326853129

hsa-miR-452

0.327630584

hsa-miR-708

0.334570407

hsa-miR-449a

0.335979203

hsa-miR-324-5p

0.340765959

hsa-miR-563

0.341269742

hsa-miR-518c*

0.342612931

hsa-miR-203

0.352915246

hsa-miR-503

0.353175924

hsa-miR-766

0.359646582

hsa-miR-629

0.360880866

hsa-miR-206

0.362474794

hsa-miR-224

0.364516645

hsa-let-7f-2*

0.366210573

hsa-miR-519c-3p

0.367820224

hsa-miR-92a

0.367943927

hsa-miR-3117

0.370339461

hsa-miR-520g

0.371457273

hsa-miR-186*

0.374081917

hsa-let-7e

0.375296918

hsa-miR-760

0.375784967

hsa-miR-92b

0.377969693

hsa-miR-141*

0.379831184

hsa-miR-1285

0.384964133

hsa-miR-592

0.386164656

hsa-miR-4294

0.386254783

hsa-miR-374b*

0.38729375

hsa-miR-1281

0.390058218

hsa-miR-517b

0.392038992

hsa-miR-498

0.392616015

hsa-miR-3130-3p

0.393166196

hsa-miR-627

0.393191574

hsa-miR-27a

0.398216183

hsa-miR-378*

0.399985215

hsa-miR-615-3p

0.402819483

hsa-miR-200a

0.404397774

hsa-miR-211

0.414602939

hsa-miR-1247

0.416225956

hsa-miR-192*

0.426068366

hsa-miR-140-3p

0.429541345

hsa-miR-720

0.433876299

hsa-miR-185

0.434501975

hsa-miR-139-3p

0.434702598

hsa-miR-26b*

0.436900606

hsa-miR-19a

0.450211604

hsa-miR-106b*

0.456860957

hsa-let-7f-1*

0.461207378

hsa-miR-17

0.466225964

hsa-miR-193a-3p

0.48933176

hsa-miR-505

0.49197972

hsa-miR-4303

0.49519988

hsa-miR-20b

0.501280523

hsa-miR-663b

0.503721921

hsa-miR-572

0.511263666

hsa-miR-520h

0.512149811

hsa-miR-452

0.514497954

hsa-miR-877*

0.515451959

hsa-miR-32

0.516418189

hsa-miR-142-3p

0.516489358

hsa-miR-182*

0.516686006

hsa-miR-500a*

0.516943149

hsa-miR-92a-1*

0.520660279

hsa-miR-146a

0.52326837

hsa-miR-519e*

0.528470761

hsa-miR-330-3p

0.530022

hsa-miR-3149

0.534151757

hsa-miR-505*

0.534690996

hsa-miR-194

0.542290296

hsa-miR-223*

0.548636358

hsa-miR-22

0.549606388

hsa-miR-3065-3p

0.550306134

hsa-miR-532-3p

0.552706085

hsa-miR-186

0.553186363

hsa-miR-514

0.556558287

hsa-miR-181a-2*

0.562339601

hsa-miR-517c

0.563173413

hsa-miR-520c-3p

0.567823686

hsa-miR-383

0.570299435

hsa-miR-193b

0.57727719

hsa-miR-151-5p

0.58269495

hsa-miR-571

0.583124558

hsa-miR-564

0.583241971

hsa-miR-629*

0.583664107

hsa-miR-33b

0.597571991

hsa-miR-23b

0.598079777

hsa-miR-625*

0.602369161

hsa-miR-570

0.607832752

hsa-miR-125b-1*

0.611830465

hsa-let-7d

0.622728644

hsa-miR-339-3p

0.625969068

hsa-miR-4311

0.628801392

hsa-miR-1274b

0.632143592

hsa-miR-517a

0.632268461

hsa-miR-135b

0.635360359

hsa-miR-106a*

0.636716065

hsa-miR-654-5p

0.637139763

hsa-miR-548c-3p

0.644048056

hsa-miR-1250

0.646590666

hsa-miR-320e

0.64994976

hsa-miR-519d

0.651627036

hsa-miR-129*

0.657553635

hsa-miR-454*

0.659080036

hsa-let-7b*

0.659839962

hsa-miR-501-3p

0.660247054

hsa-miR-27b

0.664035559

hsa-miR-195*

0.664901235

hsa-miR-892b

0.66894801

hsa-miR-3124

0.675301037

hsa-miR-361-3p

0.679774426

hsa-miR-1227

0.679782637

hsa-miR-30e

0.705863991

hsa-miR-149

0.706296982

hsa-miR-216a

0.707939555

hsa-miR-4317

0.709028054

hsa-miR-4317

0.717938392

hsa-miR-664

0.720975375

hsa-miR-20a

0.721833308

hsa-miR-30d*

0.724890946

hsa-miR-320b

0.724892723

hsa-miR-1305

0.725969281

hsa-miR-520f

0.751645689

hsa-miR-502-5p

0.761205449

hsa-miR-200a*

0.762508125

hsa-miR-320d

0.764923707

hsa-miR-15a*

0.767469235

hsa-miR-128

0.773586089

hsa-miR-125a-5p

0.779412752

hsa-miR-585

0.783715059

hsa-miR-365

0.785314741

hsa-miR-340

0.786279007

hsa-miR-4254

0.7894768

hsa-miR-484

0.790082967

hsa-miR-107

0.79995333

hsa-miR-425

0.805278875

hsa-miR-193b*

0.812867892

hsa-miR-2355-5p

0.815894668

hsa-miR-4323

0.818790298

hsa-miR-23b*

0.822237865

hsa-let-7d

0.82349526

hsa-miR-200a

0.827964787

hsa-miR-767-3p

0.834400902

hsa-miR-26a

0.84110696

hsa-miR-224*

0.84799807

hsa-miR-320c

0.850768882

hsa-miR-4284

0.852246151

hsa-miR-1208

0.853217304

hsa-miR-33a

0.853301223

hsa-miR-188-3p

0.860914223

hsa-miR-3189

0.861749658

hsa-miR-4310

0.861898597

hsa-miR-196a

0.864638403

hsa-miR-1323

0.87317539

hsa-miR-2116*

0.874922103

hsa-miR-885-5p

0.878463395

hsa-miR-873

0.878606377

hsa-miR-491-5p

0.879527605

hsa-miR-326

0.883840076

hsa-miR-182

0.891110575

hsa-miR-449c

0.893487619

hsa-miR-34b

0.89713762

hsa-miR-4254

0.907978848

hsa-miR-532-5p

0.917550103

hsa-let-7a*

0.923232902

hsa-miR-874

0.926337354

hsa-miR-1270

0.931102697

hsa-miR-548c-5p

0.932602947

hsa-miR-342-3p

0.936021819

hsa-miR-4259

0.938634329

hsa-miR-638

0.942559409

hsa-miR-125a-5p

0.944556769

hsa-miR-548n

0.944814533

hsa-miR-1260

0.949226183

hsa-miR-519b-3p

0.951436484

hsa-miR-4313

0.952366052

hsa-miR-155

0.958206322

hsa-miR-500a*

0.962998166

hsa-miR-142-5p

0.964266785

hsa-miR-103

0.982138257

hsa-miR-887

0.989109801

hsa-miR-423-3p

0.99407937

hsa-miR-521

0.995465469

hsa-miR-518a-3p

0.997420727

**Supplementary Table 2: miRNA sequencing and expression in MSCs.** miRNA-sequencing of human BM-derived MSCs. MSCs were grown to 90% confluency and harvested at time points 0h and 24h, each in duplicates. Reads per million (RPM) of miRNAs were calculated and the data is presented as median of n=4. Reads were mapped to miRBase v.21 (mirbase.org).

**miRBase annotation**

**RPM (median)**

hsa-let-7a-2-3p

26.3

hsa-let-7a-3p

51.1

hsa-let-7a-5p

34301.3

hsa-let-7b-3p

67.8

hsa-let-7b-5p

10090.0

hsa-let-7c-3p

8.1

hsa-let-7c-5p

1727.6

hsa-let-7d-3p

148.1

hsa-let-7d-5p

794.7

hsa-let-7e-3p

37.5

hsa-let-7e-5p

2869.5

hsa-let-7f-1-3p

30.4

hsa-let-7f-2-3p

3.2

hsa-let-7f-5p

22418.0

hsa-let-7g-3p

0.0

hsa-let-7g-5p

14719.9

hsa-let-7i-3p

130.7

hsa-let-7i-5p

73083.3

hsa-miR-100-3p

4.9

hsa-miR-100-5p

256658.3

hsa-miR-101-3p

456.5

hsa-miR-101-5p

0.0

hsa-miR-103a-2-5p

0.7

hsa-miR-103a-3p

5446.5

hsa-miR-105-5p

0.0

hsa-miR-106a-5p

3.0

hsa-miR-106b-3p

192.4

hsa-miR-106b-5p

50.9

hsa-miR-107

292.8

hsa-miR-10a-3p

100.1

hsa-miR-10a-5p

10949.6

hsa-miR-10b-3p

55.8

hsa-miR-10b-5p

8943.4

hsa-miR-1179

0.0

hsa-miR-1180-3p

109.6

hsa-miR-1180-5p

0.0

hsa-miR-1185-1-3p

221.8

hsa-miR-1185-2-3p

38.2

hsa-miR-1185-5p

2.1

hsa-miR-1193

0.6

hsa-miR-1197

16.5

hsa-miR-122-5p

0.0

hsa-miR-1226-3p

1.4

hsa-miR-1226-5p

0.0

hsa-miR-1227-3p

0.0

hsa-miR-1228-3p

1.0

hsa-miR-1228-5p

1.9

hsa-miR-1229-3p

0.0

hsa-miR-1231

0.0

hsa-miR-1234-3p

0.0

hsa-miR-1236-3p

0.0

hsa-miR-1237-3p

0.0

hsa-miR-1243

0.5

hsa-miR-124-3p

0.0

hsa-miR-1244

0.6

hsa-miR-1245a

0.0

hsa-miR-1245b-3p

0.0

hsa-miR-1245b-5p

0.0

hsa-miR-1246

10.4

hsa-miR-1247-3p

0.0

hsa-miR-1247-5p

0.0

hsa-miR-1248

28.6

hsa-miR-1249-3p

9.5

hsa-miR-1249-5p

0.0

hsa-miR-1250-5p

0.0

hsa-miR-1252-3p

0.0

hsa-miR-1252-5p

0.0

hsa-miR-1254

0.7

hsa-miR-1255a

3.5

hsa-miR-1255b-2-3p

0.0

hsa-miR-1255b-5p

0.5

hsa-miR-1256

0.0

hsa-miR-1257

0.0

hsa-miR-125a-3p

34.1

hsa-miR-125a-5p

8804.2

hsa-miR-125b-1-3p

1245.7

hsa-miR-125b-2-3p

6.6

hsa-miR-125b-5p

24447.1

hsa-miR-1260a

1.4

hsa-miR-1260b

141.8

hsa-miR-1262

1.2

hsa-miR-1263

0.0

hsa-miR-126-3p

148.0

hsa-miR-1264

0.0

hsa-miR-1265

0.0

hsa-miR-126-5p

4.6

hsa-miR-1266-5p

0.0

hsa-miR-1267

0.2

hsa-miR-1268a

22.5

hsa-miR-1268b

24.4

hsa-miR-1269a

0.0

hsa-miR-1269b

0.0

hsa-miR-1270

1.3

hsa-miR-1271-3p

1.7

hsa-miR-1271-5p

66.3

hsa-miR-1273a

0.0

hsa-miR-1273c

1.1

hsa-miR-1273d

0.0

hsa-miR-1273e

0.0

hsa-miR-1273f

0.0

hsa-miR-1273g-3p

0.6

hsa-miR-1273g-5p

0.0

hsa-miR-1273h-5p

0.0

hsa-miR-127-3p

7700.1

hsa-miR-1275

5.9

hsa-miR-127-5p

46.5

hsa-miR-1276

0.0

hsa-miR-1277-3p

0.5

hsa-miR-1277-5p

4.6

hsa-miR-1278

12.2

hsa-miR-128-1-5p

4.0

hsa-miR-128-3p

531.7

hsa-miR-1284

1.0

hsa-miR-1285-3p

10.1

hsa-miR-1285-5p

0.0

hsa-miR-1287-3p

0.0

hsa-miR-1287-5p

36.9

hsa-miR-1288-3p

0.0

hsa-miR-1289

0.0

hsa-miR-1291

3.2

hsa-miR-129-1-3p

0.0

hsa-miR-129-2-3p

3.1

hsa-miR-1292-5p

2.8

hsa-miR-1293

0.3

hsa-miR-1294

7.4

hsa-miR-129-5p

8.2

hsa-miR-1296-3p

0.0

hsa-miR-1296-5p

63.7

hsa-miR-1297

0.2

hsa-miR-1299

0.7

hsa-miR-1301-3p

13.8

hsa-miR-1302

0.0

hsa-miR-1303

3.0

hsa-miR-1304-3p

11.5

hsa-miR-1304-5p

3.4

hsa-miR-1305

0.4

hsa-miR-1306-5p

3.5

hsa-miR-1307-3p

110.1

hsa-miR-1307-5p

22.5

hsa-miR-130a-3p

26.7

hsa-miR-130b-3p

13.5

hsa-miR-130b-5p

30.9

hsa-miR-1322

0.0

hsa-miR-1323

0.0

hsa-miR-132-3p

78.5

hsa-miR-132-5p

56.0

hsa-miR-133a-3p

0.0

hsa-miR-133a-5p

0.0

hsa-miR-133b

0.0

hsa-miR-1343-3p

2.0

hsa-miR-1343-5p

0.4

hsa-miR-134-3p

2.8

hsa-miR-134-5p

705.9

hsa-miR-135a-5p

0.0

hsa-miR-135b-3p

0.0

hsa-miR-135b-5p

3.1

hsa-miR-136-3p

469.6

hsa-miR-136-5p

284.4

hsa-miR-137

191.6

hsa-miR-138-1-3p

11.5

hsa-miR-138-5p

214.6

hsa-miR-139-3p

0.0

hsa-miR-139-5p

2.7

hsa-miR-1-3p

60.1

hsa-miR-140-3p

450.7

hsa-miR-140-5p

1444.8

hsa-miR-141-3p

0.5

hsa-miR-142-3p

0.6

hsa-miR-142-5p

0.0

hsa-miR-143-3p

105213.3

hsa-miR-143-5p

1393.0

hsa-miR-144-3p

0.0

hsa-miR-144-5p

0.0

hsa-miR-145-3p

787.7

hsa-miR-145-5p

4871.8

hsa-miR-1468-5p

1.0

hsa-miR-146a-5p

9.3

hsa-miR-146b-3p

0.5

hsa-miR-146b-5p

26.3

hsa-miR-147b

0.0

hsa-miR-148a-3p

12669.4

hsa-miR-148a-5p

44.3

hsa-miR-148b-3p

1030.8

hsa-miR-148b-5p

0.3

hsa-miR-149-3p

0.0

hsa-miR-149-5p

61.5

hsa-miR-150-5p

0.0

hsa-miR-151a-3p

1840.9

hsa-miR-151a-5p

89.0

hsa-miR-151b

3.0

hsa-miR-152-3p

2892.9

hsa-miR-152-5p

30.2

hsa-miR-153-3p

0.9

hsa-miR-1538

0.0

hsa-miR-154-3p

27.6

hsa-miR-154-5p

34.7

hsa-miR-155-3p

0.0

hsa-miR-155-5p

237.9

hsa-miR-15a-3p

0.2

hsa-miR-15a-5p

26.6

hsa-miR-15b-3p

25.8

hsa-miR-15b-5p

112.9

hsa-miR-16-1-3p

1.6

hsa-miR-16-2-3p

27.7

hsa-miR-16-5p

680.2

hsa-miR-17-3p

2.7

hsa-miR-17-5p

389.1

hsa-miR-181a-2-3p

384.7

hsa-miR-181a-3p

278.3

hsa-miR-181a-5p

3051.4

hsa-miR-181b-2-3p

1.0

hsa-miR-181b-3p

9.5

hsa-miR-181b-5p

342.3

hsa-miR-181c-3p

15.5

hsa-miR-181c-5p

6.3

hsa-miR-181d-3p

0.0

hsa-miR-181d-5p

39.5

hsa-miR-182-3p

0.0

hsa-miR-182-5p

3.8

hsa-miR-1827

1.2

hsa-miR-183-5p

1.3

hsa-miR-184

0.3

hsa-miR-185-3p

15.6

hsa-miR-185-5p

714.6

hsa-miR-186-3p

0.0

hsa-miR-186-5p

433.5

hsa-miR-187-3p

0.0

hsa-miR-188-3p

0.0

hsa-miR-188-5p

6.6

hsa-miR-18a-3p

1.9

hsa-miR-18a-5p

12.9

hsa-miR-18b-5p

0.5

hsa-miR-1908-3p

0.0

hsa-miR-1908-5p

0.9

hsa-miR-1909-3p

0.3

hsa-miR-190a-3p

0.0

hsa-miR-190a-5p

1.4

hsa-miR-190b

0.0

hsa-miR-1910-3p

0.0

hsa-miR-1910-5p

0.0

hsa-miR-1913

0.2

hsa-miR-191-3p

3.0

hsa-miR-1914-5p

0.0

hsa-miR-191-5p

3482.1

hsa-miR-192-3p

0.0

hsa-miR-192-5p

63.0

hsa-miR-193a-3p

17.5

hsa-miR-193a-5p

551.1

hsa-miR-193b-3p

217.8

hsa-miR-193b-5p

25.6

hsa-miR-194-3p

0.6

hsa-miR-194-5p

28.6

hsa-miR-195-3p

7.8

hsa-miR-195-5p

30.6

hsa-miR-196a-3p

1.0

hsa-miR-196a-5p

577.5

hsa-miR-196b-3p

0.3

hsa-miR-196b-5p

376.7

hsa-miR-1972

0.0

hsa-miR-1973

2.6

hsa-miR-197-3p

200.8

hsa-miR-197-5p

0.3

hsa-miR-198

0.0

hsa-miR-199a-3p|hsa-miR-199b-3p

27710.8

hsa-miR-199a-5p

13945.3

hsa-miR-199b-5p

681.3

hsa-miR-19a-3p

32.5

hsa-miR-19b-1-5p

0.4

hsa-miR-19b-3p

158.9

hsa-miR-200a-3p

1.1

hsa-miR-200a-5p

0.0

hsa-miR-200b-3p

3.1

hsa-miR-200b-5p

0.0

hsa-miR-200c-3p

5.6

hsa-miR-200c-5p

0.0

hsa-miR-203a-3p

12.5

hsa-miR-203a-5p

0.0

hsa-miR-203b-3p

0.0

hsa-miR-204-3p

0.0

hsa-miR-204-5p

1.7

hsa-miR-205-3p

0.0

hsa-miR-205-5p

0.0

hsa-miR-206

0.6

hsa-miR-20a-3p

0.3

hsa-miR-20a-5p

1035.7

hsa-miR-20b-5p

1.0

hsa-miR-210-3p

137.6

hsa-miR-210-5p

6.9

hsa-miR-2110

2.9

hsa-miR-2114-3p

0.0

hsa-miR-2114-5p

0.2

hsa-miR-2116-3p

0.0

hsa-miR-2116-5p

0.0

hsa-miR-212-3p

1.9

hsa-miR-212-5p

7.6

hsa-miR-21-3p

327.7

hsa-miR-214-3p

607.4

hsa-miR-214-5p

313.8

hsa-miR-215-3p

0.3

hsa-miR-215-5p

0.0

hsa-miR-21-5p

113410.0

hsa-miR-216a-3p

0.0

hsa-miR-216a-5p

3.4

hsa-miR-216b-5p

0.3

hsa-miR-217

11.9

hsa-miR-218-2-3p

0.0

hsa-miR-218-5p

150.9

hsa-miR-219a-1-3p

1.4

hsa-miR-219a-2-3p

0.0

hsa-miR-219a-5p

0.0

hsa-miR-219b-3p

0.0

hsa-miR-219b-5p

0.3

hsa-miR-221-3p

22665.0

hsa-miR-221-5p

164.9

hsa-miR-222-3p

842.2

hsa-miR-222-5p

16.7

hsa-miR-22-3p

20874.5

hsa-miR-224-3p

12.0

hsa-miR-224-5p

38.0

hsa-miR-22-5p

248.5

hsa-miR-2276-3p

0.3

hsa-miR-2277-3p

0.0

hsa-miR-2277-5p

10.4

hsa-miR-2278

0.0

hsa-miR-2355-3p

6.4

hsa-miR-2355-5p

0.2

hsa-miR-23a-3p

2652.6

hsa-miR-23a-5p

4.8

hsa-miR-23b-3p

743.3

hsa-miR-23b-5p

31.2

hsa-miR-23c

1.7

hsa-miR-24-1-5p

8.9

hsa-miR-24-2-5p

6.9

hsa-miR-24-3p

16608.4

hsa-miR-2467-3p

0.0

hsa-miR-2467-5p

0.0

hsa-miR-25-3p

1318.0

hsa-miR-25-5p

1.4

hsa-miR-2682-3p

0.7

hsa-miR-2682-5p

106.1

hsa-miR-26a-1-3p

1.2

hsa-miR-26a-2-3p

12.8

hsa-miR-26a-5p

25368.8

hsa-miR-26b-3p

7.4

hsa-miR-26b-5p

526.2

hsa-miR-27a-3p

8390.5

hsa-miR-27a-5p

297.6

hsa-miR-27b-3p

39644.4

hsa-miR-27b-5p

90.7

hsa-miR-28-3p

442.3

hsa-miR-28-5p

141.4

hsa-miR-296-3p

22.0

hsa-miR-296-5p

11.2

hsa-miR-299-3p

41.9

hsa-miR-299-5p

45.4

hsa-miR-29a-3p

5440.9

hsa-miR-29a-5p

2.5

hsa-miR-29b-1-5p

10.7

hsa-miR-29b-2-5p

0.0

hsa-miR-29b-3p

79.9

hsa-miR-29c-3p

13.0

hsa-miR-29c-5p

2.0

hsa-miR-300

0.0

hsa-miR-301a-3p

2.4

hsa-miR-301a-5p

7.2

hsa-miR-301b-3p

2.7

hsa-miR-301b-5p

0.0

hsa-miR-302a-3p

2.1

hsa-miR-302a-5p

1.0

hsa-miR-302b-3p

8.4

hsa-miR-302c-3p

0.0

hsa-miR-302c-5p

0.2

hsa-miR-302d-3p

2.4

hsa-miR-3064-3p

0.0

hsa-miR-3064-5p

0.2

hsa-miR-3065-3p

0.7

hsa-miR-3065-5p

2.4

hsa-miR-3074-5p

0.0

hsa-miR-30a-3p

667.2

hsa-miR-30a-5p

1950.5

hsa-miR-30b-3p

5.9

hsa-miR-30b-5p

183.0

hsa-miR-30c-1-3p

9.6

hsa-miR-30c-2-3p

89.6

hsa-miR-30c-5p

1061.3

hsa-miR-30d-3p

8.7

hsa-miR-30d-5p

674.5

hsa-miR-30e-3p

325.6

hsa-miR-30e-5p

162.7

hsa-miR-3115

0.3

hsa-miR-3116

0.2

hsa-miR-3117-3p

0.4

hsa-miR-3118

0.0

hsa-miR-3120-3p

0.0

hsa-miR-3120-5p

0.0

hsa-miR-3121-3p

0.0

hsa-miR-3124-5p

0.0

hsa-miR-3125

0.0

hsa-miR-3126-3p

0.0

hsa-miR-3126-5p

0.3

hsa-miR-3127-3p

0.0

hsa-miR-3127-5p

0.0

hsa-miR-3128

0.0

hsa-miR-3129-3p

7.5

hsa-miR-3129-5p

1.0

hsa-miR-3130-3p

0.0

hsa-miR-3130-5p

0.0

hsa-miR-3131

0.0

hsa-miR-3133

1.5

hsa-miR-3134

0.0

hsa-miR-3135a

0.0

hsa-miR-3135b

0.3

hsa-miR-3136-3p

0.0

hsa-miR-3136-5p

1.0

hsa-miR-3137

0.3

hsa-miR-3138

0.5

hsa-miR-3139

0.0

hsa-miR-31-3p

11.2

hsa-miR-3140-3p

0.3

hsa-miR-3140-5p

0.0

hsa-miR-3141

0.0

hsa-miR-3143

0.3

hsa-miR-3144-3p

0.0

hsa-miR-3145-3p

0.0

hsa-miR-3145-5p

0.0

hsa-miR-3149

0.0

hsa-miR-3150a-5p

0.0

hsa-miR-3151-5p

0.0

hsa-miR-3152-3p

0.0

hsa-miR-3152-5p

4.1

hsa-miR-3154

0.0

hsa-miR-3155a

0.0

hsa-miR-3156-5p

0.0

hsa-miR-3157-3p

0.0

hsa-miR-3157-5p

0.3

hsa-miR-3158-3p

17.6

hsa-miR-3159

0.0

hsa-miR-31-5p

337.7

hsa-miR-3160-5p

0.0

hsa-miR-3162-5p

0.0

hsa-miR-3163

0.0

hsa-miR-3164

0.0

hsa-miR-3165

0.2

hsa-miR-3166

0.0

hsa-miR-3167

2.7

hsa-miR-3168

0.0

hsa-miR-3169

0.0

hsa-miR-3170

1.2

hsa-miR-3173-3p

0.0

hsa-miR-3173-5p

0.9

hsa-miR-3174

0.0

hsa-miR-3175

0.0

hsa-miR-3176

0.7

hsa-miR-3177-3p

1.2

hsa-miR-3177-5p

0.0

hsa-miR-3179

0.4

hsa-miR-3180

0.5

hsa-miR-3180-3p

2.2

hsa-miR-3180-5p

1.0

hsa-miR-3182

0.0

hsa-miR-3183

0.0

hsa-miR-3184-3p

0.0

hsa-miR-3184-5p

0.0

hsa-miR-3187-3p

0.7

hsa-miR-3187-5p

0.5

hsa-miR-3188

0.0

hsa-miR-3189-3p

0.0

hsa-miR-3190-3p

0.0

hsa-miR-3191-3p

0.3

hsa-miR-3192-5p

0.0

hsa-miR-3194-3p

0.0

hsa-miR-3195

28.7

hsa-miR-3196

19.7

hsa-miR-3197

0.0

hsa-miR-3198

0.0

hsa-miR-3199

0.0

hsa-miR-3200-3p

0.4

hsa-miR-320a

933.7

hsa-miR-320b

95.7

hsa-miR-320c

22.9

hsa-miR-320d

13.4

hsa-miR-323a-3p

163.4

hsa-miR-323a-5p

1.3

hsa-miR-323b-3p

86.8

hsa-miR-323b-5p

0.0

hsa-miR-32-3p

6.3

hsa-miR-324-3p

1.7

hsa-miR-324-5p

11.3

hsa-miR-32-5p

150.0

hsa-miR-326

2.0

hsa-miR-328-3p

153.3

hsa-miR-328-5p

0.2

hsa-miR-329-3p

138.6

hsa-miR-329-5p

0.7

hsa-miR-330-3p

27.5

hsa-miR-330-5p

6.8

hsa-miR-331-3p

23.5

hsa-miR-331-5p

6.3

hsa-miR-335-3p

1817.7

hsa-miR-335-5p

452.5

hsa-miR-337-3p

136.5

hsa-miR-337-5p

27.9

hsa-miR-338-3p

0.0

hsa-miR-338-5p

0.4

hsa-miR-339-3p

107.2

hsa-miR-339-5p

264.9

hsa-miR-33a-3p

2.0

hsa-miR-33a-5p

4.8

hsa-miR-33b-3p

1.9

hsa-miR-33b-5p

0.0

hsa-miR-340-3p

14.1

hsa-miR-340-5p

532.0

hsa-miR-342-3p

27.0

hsa-miR-342-5p

0.4

hsa-miR-345-5p

28.2

hsa-miR-346

0.0

hsa-miR-34a-3p

0.0

hsa-miR-34a-5p

147.1

hsa-miR-34b-3p

0.0

hsa-miR-34b-5p

0.0

hsa-miR-34c-3p

6.7

hsa-miR-34c-5p

234.7

hsa-miR-3529-5p

0.0

hsa-miR-3591-3p

0.0

hsa-miR-3591-5p

6.1

hsa-miR-3605-3p

1.2

hsa-miR-3605-5p

1.0

hsa-miR-3607-3p

20.3

hsa-miR-3607-5p

0.0

hsa-miR-3609

1.0

hsa-miR-3611

0.2

hsa-miR-3613-3p

0.0

hsa-miR-3613-5p

181.2

hsa-miR-361-3p

175.7

hsa-miR-3614-5p

0.0

hsa-miR-3615

5.5

hsa-miR-361-5p

242.8

hsa-miR-3616-3p

0.0

hsa-miR-3616-5p

0.2

hsa-miR-3617-3p

0.0

hsa-miR-3617-5p

0.9

hsa-miR-3618

0.0

hsa-miR-3619-3p

0.0

hsa-miR-3619-5p

0.4

hsa-miR-3620-3p

0.0

hsa-miR-3620-5p

0.0

hsa-miR-3622a-3p

0.0

hsa-miR-3622a-5p

0.6

hsa-miR-362-3p

1.6

hsa-miR-362-5p

32.2

hsa-miR-363-3p

0.0

hsa-miR-3648

0.0

hsa-miR-3650

0.0

hsa-miR-3651

6.5

hsa-miR-3653-3p

4.8

hsa-miR-3653-5p

0.5

hsa-miR-3656

0.2

hsa-miR-3657

0.0

hsa-miR-3659

0.0

hsa-miR-365a-3p|hsa-miR-365b-3p

578.8

hsa-miR-365a-5p

38.6

hsa-miR-365b-5p

6.9

hsa-miR-3661

0.2

hsa-miR-3662

0.0

hsa-miR-3663-5p

0.0

hsa-miR-3664-3p

0.0

hsa-miR-3664-5p

0.2

hsa-miR-3667-3p

0.0

hsa-miR-367-3p

0.0

hsa-miR-3675-3p

0.0

hsa-miR-3675-5p

0.0

hsa-miR-3677-3p

0.3

hsa-miR-3677-5p

0.7

hsa-miR-3679-3p

0.0

hsa-miR-3679-5p

0.2

hsa-miR-3680-3p

0.0

hsa-miR-3680-5p

0.3

hsa-miR-3681-5p

0.7

hsa-miR-3682-3p

0.0

hsa-miR-3682-5p

0.0

hsa-miR-3684

0.0

hsa-miR-3685

0.0

hsa-miR-3687

0.4

hsa-miR-3688-3p

0.5

hsa-miR-3691-3p

0.0

hsa-miR-3692-3p

0.0

hsa-miR-369-3p

337.3

hsa-miR-369-5p

251.9

hsa-miR-370-3p

2723.5

hsa-miR-370-5p

6.9

hsa-miR-372-3p

0.0

hsa-miR-373-3p

0.0

hsa-miR-374a-3p

133.0

hsa-miR-374a-5p

286.8

hsa-miR-374b-3p

16.0

hsa-miR-374b-5p

874.0

hsa-miR-374c-3p

0.0

hsa-miR-374c-5p

1.1

hsa-miR-375

0.3

hsa-miR-376a-2-5p

0.0

hsa-miR-376a-3p

43.6

hsa-miR-376a-5p

4.7

hsa-miR-376b-3p

4.1

hsa-miR-376b-5p

6.0

hsa-miR-376c-3p

149.4

hsa-miR-376c-5p

0.6

hsa-miR-377-3p

95.5

hsa-miR-377-5p

45.2

hsa-miR-378a-3p

40.6

hsa-miR-378a-5p

0.6

hsa-miR-378b

0.0

hsa-miR-378c

4.3

hsa-miR-378d

2.0

hsa-miR-378e

0.0

hsa-miR-378f

0.0

hsa-miR-378g

0.0

hsa-miR-378i

0.0

hsa-miR-379-3p

128.1

hsa-miR-379-5p

3223.4

hsa-miR-380-3p

8.6

hsa-miR-380-5p

0.0

hsa-miR-381-3p

3721.4

hsa-miR-381-5p

0.3

hsa-miR-382-3p

169.2

hsa-miR-382-5p

354.8

hsa-miR-383-5p

0.9

hsa-miR-3909

25.6

hsa-miR-3910

0.2

hsa-miR-3911

0.0

hsa-miR-3912-3p

3.1

hsa-miR-3913-5p

1.1

hsa-miR-3914

0.0

hsa-miR-3916

0.6

hsa-miR-3917

0.0

hsa-miR-3918

0.0

hsa-miR-3919

0.0

hsa-miR-3920

0.0

hsa-miR-3922-5p

0.0

hsa-miR-3925-3p

0.0

hsa-miR-3928-3p

4.1

hsa-miR-3929

0.0

hsa-miR-3934-3p

0.0

hsa-miR-3934-5p

3.5

hsa-miR-3935

0.0

hsa-miR-3936

0.0

hsa-miR-3939

0.0

hsa-miR-3940-3p

0.9

hsa-miR-3940-5p

0.0

hsa-miR-3941

0.0

hsa-miR-3942-3p

0.0

hsa-miR-3942-5p

0.0

hsa-miR-3944-3p

0.5

hsa-miR-3944-5p

0.0

hsa-miR-409-3p

1025.0

hsa-miR-409-5p

217.1

hsa-miR-410-3p

141.0

hsa-miR-410-5p

2.4

hsa-miR-411-3p

313.1

hsa-miR-411-5p

1979.3

hsa-miR-412-5p

8.4

hsa-miR-421

42.2

hsa-miR-422a

0.0

hsa-miR-423-3p

3035.2

hsa-miR-423-5p

698.7

hsa-miR-424-3p

141.7

hsa-miR-424-5p

116.7

hsa-miR-425-3p

14.7

hsa-miR-4254

0.0

hsa-miR-425-5p

37.3

hsa-miR-4284

0.0

hsa-miR-4286

9.5

hsa-miR-4288

0.0

hsa-miR-429

1.4

hsa-miR-431-3p

2.7

hsa-miR-431-5p

47.4

hsa-miR-432-3p

1.0

hsa-miR-432-5p

177.8

hsa-miR-4326

0.3

hsa-miR-433-3p

101.5

hsa-miR-433-5p

0.6

hsa-miR-4420

0.0

hsa-miR-4421

1.3

hsa-miR-4423-3p

0.0

hsa-miR-4423-5p

0.9

hsa-miR-4424

0.0

hsa-miR-4425

0.0

hsa-miR-4426

0.0

hsa-miR-4429

0.0

hsa-miR-4431

0.0

hsa-miR-4433a-3p

0.0

hsa-miR-4433b-5p

0.0

hsa-miR-4435

0.2

hsa-miR-4436b-3p

0.0

hsa-miR-4440

0.0

hsa-miR-4442

0.0

hsa-miR-4443

1.3

hsa-miR-4452

0.0

hsa-miR-4454

11.5

hsa-miR-4455

23.5

hsa-miR-4456

0.0

hsa-miR-4458

0.0

hsa-miR-4461

0.2

hsa-miR-4463

0.0

hsa-miR-4466

0.0

hsa-miR-4467

0.0

hsa-miR-4469

0.0

hsa-miR-4470

0.3

hsa-miR-4473

2.3

hsa-miR-4474-3p

0.0

hsa-miR-4477a

0.0

hsa-miR-4479

0.2

hsa-miR-4483

0.0

hsa-miR-4484

0.0

hsa-miR-4485-3p

40.4

hsa-miR-4488

11.2

hsa-miR-4491

0.0

hsa-miR-4492

0.7

hsa-miR-4494

0.0

hsa-miR-4497

257.6

hsa-miR-4498

0.0

hsa-miR-449a

0.6

hsa-miR-449b-3p

0.0

hsa-miR-449b-5p

0.0

hsa-miR-449c-5p

0.7

hsa-miR-4500

0.0

hsa-miR-4502

0.0

hsa-miR-4503

0.0

hsa-miR-4504

0.0

hsa-miR-4508

0.0

hsa-miR-450a-1-3p

4.4

hsa-miR-450a-2-3p

4.4

hsa-miR-450a-5p

270.5

hsa-miR-450b-5p

250.9

hsa-miR-4510

4.9

hsa-miR-4511

0.0

hsa-miR-4516

0.0

hsa-miR-4517

0.0

hsa-miR-451a

0.0

hsa-miR-4521

7.0

hsa-miR-4522

0.0

hsa-miR-4523

0.2

hsa-miR-4524a-3p

0.5

hsa-miR-452-5p

53.4

hsa-miR-4527

0.0

hsa-miR-4531

3.7

hsa-miR-4532

0.5

hsa-miR-4536-5p

0.6

hsa-miR-454-3p

17.9

hsa-miR-454-5p

3.9

hsa-miR-455-3p

27.3

hsa-miR-455-5p

110.3

hsa-miR-4634

0.0

hsa-miR-4636

1.1

hsa-miR-4637

0.0

hsa-miR-4638-3p

0.0

hsa-miR-4638-5p

0.0

hsa-miR-4640-3p

0.0

hsa-miR-4640-5p

0.0

hsa-miR-4645-3p

0.0

hsa-miR-4646-3p

0.2

hsa-miR-4647

0.0

hsa-miR-4649-3p

0.0

hsa-miR-4649-5p

0.0

hsa-miR-4650-3p

0.0

hsa-miR-4651

0.0

hsa-miR-4657

0.0

hsa-miR-4659a-3p

0.0

hsa-miR-4659b-3p

0.0

hsa-miR-466

0.0

hsa-miR-4660

0.0

hsa-miR-4661-3p

0.0

hsa-miR-4661-5p

0.3

hsa-miR-4662a-5p

0.2

hsa-miR-4662b

0.0

hsa-miR-4664-3p

0.0

hsa-miR-4664-5p

0.0

hsa-miR-4665-5p

0.2

hsa-miR-4667-3p

0.0

hsa-miR-4667-5p

0.0

hsa-miR-4669

0.0

hsa-miR-4670-3p

0.0

hsa-miR-4670-5p

0.0

hsa-miR-4672

0.0

hsa-miR-4676-3p

0.2

hsa-miR-4676-5p

0.0

hsa-miR-4677-3p

7.5

hsa-miR-4677-5p

0.0

hsa-miR-4680-5p

0.0

hsa-miR-4682

0.0

hsa-miR-4683

0.0

hsa-miR-4684-3p

0.6

hsa-miR-4684-5p

0.0

hsa-miR-4685-3p

0.5

hsa-miR-4685-5p

0.0

hsa-miR-4686

0.0

hsa-miR-4687-3p

0.0

hsa-miR-4687-5p

0.0

hsa-miR-4688

0.0

hsa-miR-4689

0.0

hsa-miR-4690-3p

0.0

hsa-miR-4690-5p

0.0

hsa-miR-4691-3p

0.0

hsa-miR-4695-3p

0.0

hsa-miR-4695-5p

0.0

hsa-miR-4698

0.0

hsa-miR-4699-3p

0.0

hsa-miR-4699-5p

0.0

hsa-miR-4700-3p

0.3

hsa-miR-4700-5p

0.0

hsa-miR-4704-3p

0.0

hsa-miR-4705

0.0

hsa-miR-4706

0.0

hsa-miR-4707-3p

0.0

hsa-miR-4707-5p

0.0

hsa-miR-4709-3p

0.3

hsa-miR-4709-5p

0.0

hsa-miR-4710

0.0

hsa-miR-4714-3p

0.0

hsa-miR-4714-5p

0.0

hsa-miR-4715-3p

0.0

hsa-miR-4715-5p

0.3

hsa-miR-4716-3p

0.0

hsa-miR-4716-5p

0.0

hsa-miR-4717-3p

0.0

hsa-miR-4719

0.0

hsa-miR-4720-5p

0.0

hsa-miR-4723-3p

0.0

hsa-miR-4723-5p

0.0

hsa-miR-4725-3p

0.9

hsa-miR-4725-5p

0.0

hsa-miR-4726-3p

0.0

hsa-miR-4726-5p

0.7

hsa-miR-4727-3p

0.0

hsa-miR-4728-3p

0.0

hsa-miR-4728-5p

0.0

hsa-miR-4729

0.0

hsa-miR-4731-3p

0.0

hsa-miR-4731-5p

0.0

hsa-miR-4734

0.0

hsa-miR-4735-5p

0.0

hsa-miR-4738-3p

0.2

hsa-miR-4739

0.0

hsa-miR-4741

0.0

hsa-miR-4742-3p

0.7

hsa-miR-4742-5p

0.2

hsa-miR-4743-5p

0.2

hsa-miR-4745-5p

0.0

hsa-miR-4746-5p

1.4

hsa-miR-4747-5p

0.0

hsa-miR-4748

0.0

hsa-miR-4749-3p

0.0

hsa-miR-4749-5p

0.0

hsa-miR-4750-5p

0.0

hsa-miR-4753-3p

0.0

hsa-miR-4754

0.0

hsa-miR-4755-3p

0.0

hsa-miR-4755-5p

0.0

hsa-miR-4757-3p

0.0

hsa-miR-4757-5p

0.0

hsa-miR-4761-3p

0.0

hsa-miR-4761-5p

0.0

hsa-miR-4762-3p

0.0

hsa-miR-4762-5p

0.0

hsa-miR-4764-5p

0.0

hsa-miR-4765

0.0

hsa-miR-4766-3p

0.0

hsa-miR-4767

0.0

hsa-miR-4768-5p

0.3

hsa-miR-4769-3p

0.0

hsa-miR-4773

0.0

hsa-miR-4774-5p

0.0

hsa-miR-4775

20.7

hsa-miR-4776-5p

0.0

hsa-miR-4779

0.0

hsa-miR-4781-3p

0.0

hsa-miR-4782-5p

0.0

hsa-miR-4783-3p

0.0

hsa-miR-4785

0.3

hsa-miR-4786-3p

0.0

hsa-miR-4786-5p

0.0

hsa-miR-4787-3p

1.8

hsa-miR-4788

0.0

hsa-miR-4791

0.5

hsa-miR-4792

38.8

hsa-miR-4794

0.0

hsa-miR-4795-3p

0.0

hsa-miR-4796-3p

0.0

hsa-miR-4796-5p

0.0

hsa-miR-4797-3p

0.0

hsa-miR-4797-5p

0.0

hsa-miR-4798-5p

0.5

hsa-miR-4799-5p

0.0

hsa-miR-4802-3p

0.0

hsa-miR-4803

0.7

hsa-miR-483-3p

0.9

hsa-miR-483-5p

2.2

hsa-miR-484

289.8

hsa-miR-485-3p

143.5

hsa-miR-485-5p

33.0

hsa-miR-486-3p

1.5

hsa-miR-486-5p

21.0

hsa-miR-487a-3p

24.6

hsa-miR-487a-5p

14.5

hsa-miR-487b-3p

74.2

hsa-miR-487b-5p

3.9

hsa-miR-490-3p

5.3

hsa-miR-490-5p

0.4

hsa-miR-491-3p

0.0

hsa-miR-491-5p

1.7

hsa-miR-493-3p

1021.1

hsa-miR-493-5p

1601.6

hsa-miR-494-3p

27.9

hsa-miR-494-5p

2.8

hsa-miR-495-3p

217.4

hsa-miR-495-5p

0.7

hsa-miR-496

1.5

hsa-miR-497-3p

0.0

hsa-miR-497-5p

7.7

hsa-miR-4999-5p

0.0

hsa-miR-499a-5p

3.9

hsa-miR-499b-3p

0.0

hsa-miR-499b-5p

0.0

hsa-miR-5000-3p

0.6

hsa-miR-5000-5p

0.0

hsa-miR-5001-3p

0.7

hsa-miR-5001-5p

0.0

hsa-miR-5002-5p

0.3

hsa-miR-5003-3p

0.0

hsa-miR-5003-5p

0.0

hsa-miR-5004-5p

0.0

hsa-miR-5006-3p

0.0

hsa-miR-5007-3p

0.0

hsa-miR-5008-5p

0.0

hsa-miR-5009-5p

0.0

hsa-miR-500a-3p

60.1

hsa-miR-500a-5p

5.0

hsa-miR-5010-3p

1.0

hsa-miR-5010-5p

0.0

hsa-miR-501-3p

30.0

hsa-miR-501-5p

0.0

hsa-miR-502-3p

69.1

hsa-miR-503-3p

0.6

hsa-miR-503-5p

126.2

hsa-miR-504-5p

0.0

hsa-miR-505-3p

11.0

hsa-miR-505-5p

1.4

hsa-miR-508-3p

0.0

hsa-miR-5088-3p

0.0

hsa-miR-5090

0.0

hsa-miR-5091

0.0

hsa-miR-5092

0.0

hsa-miR-509-3-5p

0.0

hsa-miR-5094

0.0

hsa-miR-5095

0.2

hsa-miR-5100

0.3

hsa-miR-510-5p

0.0

hsa-miR-511-5p

0.0

hsa-miR-512-3p

0.0

hsa-miR-514a-3p

0.3

hsa-miR-514a-5p

0.0

hsa-miR-5187-5p

0.0

hsa-miR-5188

0.2

hsa-miR-5189-5p

0.0

hsa-miR-5193

0.0

hsa-miR-5195-5p

0.0

hsa-miR-519a-3p

0.0

hsa-miR-520a-3p

0.0

hsa-miR-532-3p

26.7

hsa-miR-532-5p

795.5

hsa-miR-539-3p

77.8

hsa-miR-539-5p

6.5

hsa-miR-541-3p

3.4

hsa-miR-541-5p

1.3

hsa-miR-542-3p

171.6

hsa-miR-542-5p

2.1

hsa-miR-543

183.9

hsa-miR-544a

0.0

hsa-miR-544b

0.5

hsa-miR-545-3p

0.3

hsa-miR-545-5p

0.0

hsa-miR-548a-3p

1.4

hsa-miR-548a-5p

0.0

hsa-miR-548ab

0.0

hsa-miR-548ac

0.0

hsa-miR-548ad-5p|hsa-miR-548ae-5p

0.0

hsa-miR-548ag

0.0

hsa-miR-548ah-3p

1.4

hsa-miR-548aj-3p

0.0

hsa-miR-548ak

0.0

hsa-miR-548al

0.3

hsa-miR-548am-3p

0.0

hsa-miR-548ao-3p

0.0

hsa-miR-548aq-3p

1.7

hsa-miR-548aq-5p

0.0

hsa-miR-548ar-3p

2.2

hsa-miR-548as-5p

0.0

hsa-miR-548at-3p

0.0

hsa-miR-548at-5p

0.0

hsa-miR-548au-5p

0.0

hsa-miR-548av-3p

0.0

hsa-miR-548av-5p

0.0

hsa-miR-548aw

0.0

hsa-miR-548ay-3p

2.4

hsa-miR-548ay-5p

1.2

hsa-miR-548az-5p

0.0

hsa-miR-548b-5p

0.0

hsa-miR-548ba

0.0

hsa-miR-548bb-3p

0.0

hsa-miR-548c-3p

0.0

hsa-miR-548c-5p|hsa-miR-548o-5p|hsa-miR-548am-5p

0.0

hsa-miR-548d-3p

0.0

hsa-miR-548d-5p

1.7

hsa-miR-548e-3p

22.0

hsa-miR-548e-5p

0.0

hsa-miR-548h-3p|hsa-miR-548z

0.5

hsa-miR-548h-5p

0.8

hsa-miR-548i

0.3

hsa-miR-548j-3p

2.0

hsa-miR-548j-5p

2.8

hsa-miR-548k

14.5

hsa-miR-548l

0.5

hsa-miR-548n

0.6

hsa-miR-548o-3p

25.3

hsa-miR-548p

0.0

hsa-miR-548q

0.0

hsa-miR-548s

0.0

hsa-miR-548t-3p|hsa-miR-548aa

2.0

hsa-miR-548t-5p

0.0

hsa-miR-548u

5.1

hsa-miR-548w

2.7

hsa-miR-549a

18.6

hsa-miR-550a-3-5p

0.2

hsa-miR-550a-3p

2.1

hsa-miR-550a-5p

6.3

hsa-miR-550b-2-5p

0.0

hsa-miR-551a

0.0

hsa-miR-551b-3p

0.0

hsa-miR-551b-5p

0.0

hsa-miR-552-3p

0.0

hsa-miR-556-3p

0.0

hsa-miR-556-5p

0.0

hsa-miR-5571-3p

0.0

hsa-miR-5579-3p

0.2

hsa-miR-5579-5p

0.9

hsa-miR-5581-3p

0.9

hsa-miR-5582-3p

0.3

hsa-miR-5583-3p

0.0

hsa-miR-5585-3p

5.4

hsa-miR-5585-5p

0.0

hsa-miR-5588-5p

0.0

hsa-miR-5591-3p

0.0

hsa-miR-561-3p

0.0

hsa-miR-561-5p

0.0

hsa-miR-566

0.0

hsa-miR-567

0.0

hsa-miR-5683

0.0

hsa-miR-5684

0.0

hsa-miR-5690

0.0

hsa-miR-5692c

0.0

hsa-miR-5695

0.0

hsa-miR-5696

0.0

hsa-miR-5697

0.0

hsa-miR-5698

0.3

hsa-miR-5699-3p

2.7

hsa-miR-5699-5p

2.6

hsa-miR-5701

25.3

hsa-miR-570-3p

0.5

hsa-miR-570-5p|hsa-miR-548ai

0.0

hsa-miR-5706

0.0

hsa-miR-5708

0.0

hsa-miR-573

0.0

hsa-miR-574-3p

451.0

hsa-miR-574-5p

373.1

hsa-miR-576-3p

15.2

hsa-miR-576-5p

10.9

hsa-miR-5787

0.0

hsa-miR-579-3p

0.4

hsa-miR-579-5p

1.7

hsa-miR-580-3p

0.5

hsa-miR-580-5p

0.0

hsa-miR-581

0.0

hsa-miR-582-3p

5.4

hsa-miR-582-5p

1.4

hsa-miR-584-3p

0.0

hsa-miR-584-5p

33.3

hsa-miR-585-3p

0.5

hsa-miR-585-5p

0.0

hsa-miR-586

0.0

hsa-miR-588

0.0

hsa-miR-589-3p

0.0

hsa-miR-589-5p

42.9

hsa-miR-590-3p

20.3

hsa-miR-590-5p

0.7

hsa-miR-592

0.0

hsa-miR-597-3p

0.0

hsa-miR-597-5p

0.5

hsa-miR-598-3p

31.0

hsa-miR-598-5p

0.0

hsa-miR-605-3p

0.3

hsa-miR-605-5p

0.0

hsa-miR-6083

0.0

hsa-miR-6087

0.2

hsa-miR-6089

0.0

hsa-miR-610

0.0

hsa-miR-612

0.3

hsa-miR-6129

0.0

hsa-miR-6130

3.4

hsa-miR-6131

0.0

hsa-miR-6134

0.4

hsa-miR-615-3p

557.0

hsa-miR-615-5p

7.8

hsa-miR-616-3p

0.0

hsa-miR-616-5p

1.1

hsa-miR-618

47.4

hsa-miR-619-5p

8.5

hsa-miR-622

0.0

hsa-miR-624-3p

0.0

hsa-miR-624-5p

2.2

hsa-miR-625-3p

19.0

hsa-miR-625-5p

1.5

hsa-miR-627-3p

1.0

hsa-miR-627-5p

0.2

hsa-miR-628-3p

0.0

hsa-miR-628-5p

29.2

hsa-miR-629-3p

1.0

hsa-miR-629-5p

35.0

hsa-miR-630

0.0

hsa-miR-635

0.0

hsa-miR-636

0.0

hsa-miR-641

4.8

hsa-miR-642a-3p

1.1

hsa-miR-642a-5p

1.4

hsa-miR-642b-3p

0.0

hsa-miR-647

0.0

hsa-miR-649

0.0

hsa-miR-6500-3p

6.3

hsa-miR-6501-3p

0.0

hsa-miR-6501-5p

0.5

hsa-miR-6505-3p

0.0

hsa-miR-6505-5p

0.7

hsa-miR-6506-5p

0.0

hsa-miR-6508-3p

0.0

hsa-miR-6508-5p

0.0

hsa-miR-6509-3p

0.3

hsa-miR-6509-5p

0.0

hsa-miR-6510-3p

0.0

hsa-miR-6511a-3p

0.9

hsa-miR-6511a-5p

0.0

hsa-miR-6511b-3p

3.4

hsa-miR-6511b-5p

0.6

hsa-miR-6513-3p

0.0

hsa-miR-6513-5p

0.0

hsa-miR-651-3p

0.0

hsa-miR-6514-3p

0.0

hsa-miR-6514-5p

0.0

hsa-miR-6515-3p

0.0

hsa-miR-6515-5p

0.7

hsa-miR-651-5p

11.3

hsa-miR-6516-3p

0.7

hsa-miR-6516-5p

0.5

hsa-miR-652-3p

6.3

hsa-miR-652-5p

0.0

hsa-miR-654-3p

1349.6

hsa-miR-654-5p

161.1

hsa-miR-655-3p

66.9

hsa-miR-655-5p

0.9

hsa-miR-656-3p

158.9

hsa-miR-656-5p

0.6

hsa-miR-659-5p

3.4

hsa-miR-660-3p

0.6

hsa-miR-660-5p

26.9

hsa-miR-663a

0.0

hsa-miR-663b

0.0

hsa-miR-664a-3p

41.0

hsa-miR-664a-5p

10.4

hsa-miR-664b-3p

4.2

hsa-miR-664b-5p

0.3

hsa-miR-665

15.8

hsa-miR-668-3p

2.3

hsa-miR-671-3p

33.6

hsa-miR-6715a-3p

0.0

hsa-miR-6715b-3p

0.0

hsa-miR-671-5p

12.6

hsa-miR-6716-3p

0.5

hsa-miR-6720-3p

2.4

hsa-miR-6720-5p

0.2

hsa-miR-6721-5p

0.0

hsa-miR-6724-5p

0.2

hsa-miR-6726-3p

0.0

hsa-miR-6726-5p

0.0

hsa-miR-6727-3p

0.0

hsa-miR-6727-5p

0.0

hsa-miR-6728-5p

0.0

hsa-miR-6729-5p

0.0

hsa-miR-6731-5p

0.0

hsa-miR-6732-3p

0.3

hsa-miR-6733-3p

0.0

hsa-miR-6733-5p

0.0

hsa-miR-6734-3p

0.0

hsa-miR-6734-5p

0.3

hsa-miR-6735-3p

0.0

hsa-miR-6735-5p

0.6

hsa-miR-6736-3p

0.0

hsa-miR-6736-5p

0.0

hsa-miR-6737-3p

0.0

hsa-miR-6738-3p

0.0

hsa-miR-6739-5p

0.0

hsa-miR-6741-3p

0.0

hsa-miR-6741-5p

0.0

hsa-miR-6742-3p

0.0

hsa-miR-6743-3p

0.0

hsa-miR-6746-3p

0.2

hsa-miR-6747-3p

0.0

hsa-miR-6748-5p

0.0

hsa-miR-6749-3p

0.0

hsa-miR-6751-5p

0.0

hsa-miR-6753-3p

0.0

hsa-miR-6753-5p

0.0

hsa-miR-6754-3p

0.0

hsa-miR-6754-5p

0.0

hsa-miR-6755-3p

0.0

hsa-miR-6755-5p

0.0

hsa-miR-675-5p

0.0

hsa-miR-6756-3p

0.0

hsa-miR-6757-5p

0.0

hsa-miR-6758-5p

0.0

hsa-miR-6759-5p

0.0

hsa-miR-6761-3p

0.0

hsa-miR-6761-5p

0.0

hsa-miR-6763-3p

0.0

hsa-miR-6763-5p

0.0

hsa-miR-6764-5p

0.0

hsa-miR-6765-5p

0.0

hsa-miR-6766-3p

0.0

hsa-miR-6766-5p

0.0

hsa-miR-6767-5p

0.0

hsa-miR-6769b-3p

0.0

hsa-miR-6769b-5p

0.0

hsa-miR-6770-3p

0.0

hsa-miR-6770-5p

0.5

hsa-miR-6772-3p

0.0

hsa-miR-6775-3p

0.0

hsa-miR-6776-3p

0.2

hsa-miR-6779-5p

0.0

hsa-miR-6780a-5p

0.0

hsa-miR-6780b-3p

0.0

hsa-miR-6782-3p

0.0

hsa-miR-6783-3p

0.0

hsa-miR-6783-5p

0.0

hsa-miR-6785-5p

0.0

hsa-miR-6786-3p

0.0

hsa-miR-6787-3p

0.0

hsa-miR-6788-3p

0.0

hsa-miR-6789-3p

0.3

hsa-miR-6789-5p

0.0

hsa-miR-6793-3p

0.0

hsa-miR-6793-5p

0.0

hsa-miR-6796-5p

0.0

hsa-miR-6797-3p

0.0

hsa-miR-6797-5p

0.0

hsa-miR-6798-3p

0.0

hsa-miR-6799-3p

0.0

hsa-miR-6801-5p

0.0

hsa-miR-6802-3p

0.0

hsa-miR-6802-5p

0.0

hsa-miR-6803-3p

0.0

hsa-miR-6804-5p

0.0

hsa-miR-6805-5p

0.0

hsa-miR-6806-3p

0.0

hsa-miR-6808-3p

0.0

hsa-miR-6812-3p

0.0

hsa-miR-6812-5p

0.0

hsa-miR-6816-5p

0.0

hsa-miR-6818-5p

0.0

hsa-miR-6820-3p

0.2

hsa-miR-6820-5p

0.0

hsa-miR-6824-3p

0.0

hsa-miR-6826-3p

0.0

hsa-miR-6826-5p

0.0

hsa-miR-6827-3p

0.2

hsa-miR-6827-5p

1.8

hsa-miR-6830-3p

0.0

hsa-miR-6830-5p

0.0

hsa-miR-6833-3p

0.0

hsa-miR-6834-3p

0.0

hsa-miR-6836-3p

0.0

hsa-miR-6837-3p

0.3

hsa-miR-6838-5p

0.3

hsa-miR-6839-3p

0.0

hsa-miR-6839-5p

0.0

hsa-miR-6842-3p

1.4

hsa-miR-6843-3p

0.0

hsa-miR-6846-5p

0.0

hsa-miR-6851-5p

0.0

hsa-miR-6852-5p

0.0

hsa-miR-6853-3p

0.0

hsa-miR-6854-3p

0.4

hsa-miR-6854-5p

0.7

hsa-miR-6855-5p

0.0

hsa-miR-6857-3p

0.0

hsa-miR-6858-3p

0.0

hsa-miR-6859-3p

0.0

hsa-miR-6859-5p

0.0

hsa-miR-6860

0.0

hsa-miR-6861-5p

0.0

hsa-miR-6862-5p

0.0

hsa-miR-6864-5p

0.0

hsa-miR-6865-5p

0.0

hsa-miR-6866-3p

0.0

hsa-miR-6866-5p

1.1

hsa-miR-6868-3p

0.6

hsa-miR-6869-5p

0.0

hsa-miR-6870-3p

0.0

hsa-miR-6873-3p

0.5

hsa-miR-6874-3p

0.0

hsa-miR-6874-5p

0.0

hsa-miR-6875-5p

0.0

hsa-miR-6876-3p

0.0

hsa-miR-6876-5p

0.0

hsa-miR-6877-5p

0.0

hsa-miR-6878-3p

0.0

hsa-miR-6878-5p

0.0

hsa-miR-6881-3p

0.0

hsa-miR-6882-5p

0.0

hsa-miR-6883-3p

0.0

hsa-miR-6884-3p

0.0

hsa-miR-6885-3p

0.0

hsa-miR-6886-3p

0.0

hsa-miR-6886-5p

0.0

hsa-miR-6888-3p

0.0

hsa-miR-6889-3p

0.0

hsa-miR-6889-5p

0.0

hsa-miR-6891-3p

0.0

hsa-miR-6892-5p

0.2

hsa-miR-6894-3p

0.0

hsa-miR-708-3p

55.2

hsa-miR-708-5p

339.3

hsa-miR-7108-5p

0.0

hsa-miR-7109-5p

0.0

hsa-miR-711

0.0

hsa-miR-7110-3p

0.2

hsa-miR-7110-5p

0.0

hsa-miR-7111-3p

0.0

hsa-miR-7111-5p

0.0

hsa-miR-7113-3p

0.0

hsa-miR-7114-3p

0.0

hsa-miR-7-1-3p

11.3

hsa-miR-7156-5p

0.0

hsa-miR-7158-5p

0.0

hsa-miR-7159-3p

0.0

hsa-miR-7161-3p

0.0

hsa-miR-7161-5p

0.0

hsa-miR-7-2-3p

0.0

hsa-miR-744-3p

3.5

hsa-miR-744-5p

392.1

hsa-miR-758-3p

296.5

hsa-miR-758-5p

0.4

hsa-miR-7-5p

146.5

hsa-miR-760

0.3

hsa-miR-762

0.0

hsa-miR-7641

11.4

hsa-miR-766-3p

2.2

hsa-miR-766-5p

0.0

hsa-miR-767-5p

0.0

hsa-miR-769-3p

5.6

hsa-miR-769-5p

190.3

hsa-miR-7703

0.0

hsa-miR-7704

118.1

hsa-miR-7705

0.0

hsa-miR-770-5p

0.0

hsa-miR-7706

20.8

hsa-miR-7845-5p

0.0

hsa-miR-7851-3p

0.0

hsa-miR-7853-5p

0.0

hsa-miR-7854-3p

0.0

hsa-miR-7974

3.2

hsa-miR-7975

0.5

hsa-miR-7976

0.0

hsa-miR-7977

0.3

hsa-miR-8058

0.0

hsa-miR-8063

0.0

hsa-miR-8485

0.0

hsa-miR-873-3p

0.0

hsa-miR-874-3p

2.7

hsa-miR-874-5p

0.2

hsa-miR-877-3p

0.7

hsa-miR-877-5p

1.2

hsa-miR-885-3p

0.0

hsa-miR-885-5p

0.0

hsa-miR-887-3p

0.6

hsa-miR-887-5p

0.0

hsa-miR-889-3p

216.9

hsa-miR-889-5p

1.0

hsa-miR-92a-1-5p

2.7

hsa-miR-92a-3p

2125.7

hsa-miR-92b-3p

545.4

hsa-miR-92b-5p

1.7

hsa-miR-933

0.0

hsa-miR-93-3p

4.5

hsa-miR-935

0.0

hsa-miR-93-5p

618.0

hsa-miR-937-3p

0.6

hsa-miR-939-3p

0.0

hsa-miR-939-5p

0.0

hsa-miR-9-3p

0.6

hsa-miR-940

1.5

hsa-miR-941

91.0

hsa-miR-942-3p

0.0

hsa-miR-942-5p

1.1

hsa-miR-944

0.0

hsa-miR-95-3p

4.5

hsa-miR-9-5p

8.2

hsa-miR-96-5p

1.5

hsa-miR-98-3p

2.2

hsa-miR-98-5p

546.0

hsa-miR-99a-3p

1.1

hsa-miR-99a-5p

3912.5

hsa-miR-99b-3p

143.2

hsa-miR-99b-5p

7186.4

**Supplementary Table 3: Protein secretion after miRNA overexpression in MSCs.** Proteome Profiler cytokine array analysis of MSC CM after miR-Ctrl or miR-1246 overexpression. The data is presented in arbitrary units (a.u.) after background subtraction. A protein was determined to be expressed when expression levels were > 0.5 (a.u.).

**Secreted protein**

**miR-Ctrl #1**

**miR-Ctrl #2**

**miR-1246 #1**

**miR-1246 #2**

PAI-1

17.15

20.055

16.425

12.385

CCL2/MCP-1

0.265

0.18

8.38

9.065

MIF

5.005

5.285

6.26

6.465

CCL5/RANTES

0.075

-0.03

3.755

3.56

IL-6

0.27

0.25

1.82

1.605

IP-10

0.08

-0.02

0.225

0.185

C5/C5a

0.1

0.02

0.115

0.175

IL-13

0.22

0.105

0.13

0.16

CD40

0.245

0.155

0.07

0.215

INF-gamma

0.105

0.095

0.095

0.145

CXCL1

0.075

-0.075

0.085

0.105

IL-17E

0.095

-0.02

0.095

0.06

GM-CSF

0.14

-0.045

0.045

0.105

IL-4

0.065

-0.055

0.075

0.07

IL-17

0.07

-0.06

0.065

0.08

IL-23

0.14

0.085

0.105

0.03

IL-5

0.065

-0.055

0.095

0.035

CCL1

0.075

-0.065

0.07

0.05

CCL3

0.075

-0.025

0.085

0.02

G-CSF

0.11

-0.025

0

0.1

IL-1ra

0.095

-0.025

0.035

0.05

sTREM-1

0.05

-0.08

0.025

0.055

CD54

0.025

-0.02

0.025

0.04

IL-16

0.125

-0.03

-0.01

0.075

IL-32a

0.075

-0.08

0.06

-0.015

IL-2

0.065

-0.065

-0.005

0.04

IL-27

0.045

-0.035

0.02

0.01

IL1alfa

0.07

-0.06

0.04

-0.015

IL-12

0.06

-0.035

0.015

0.01

IL-8

0.02

-0.075

0.02

-0.01

I-TAC

0.07

-0.04

-0.01

0.02

IL1beta

0.03

-0.055

-0.015

0.02

IL-10

0.065

-0.06

0.03

-0.025

CXCL12

0.06

-0.025

0

0.005

CCL4

0.01

-0.045

0.005

-0.045

TNFalfa

0.06

-0.06

-0.05

0.005

**Supplementary Table 4: Predicted and downregulated target genes of miR-1246.** List of potential target genes of miR-1246 after gene array-based differential expression analysis. Listed genes were significantly downregulated (p<0.01 and reduction of >20%) after miR-1246 overexpression in MSCs of two different donors compared to miR-Ctrl. In addition, all genes were predicted targets of miR-1246 (TargetScan.org). Data is presented as mean with n=3 for each condition in each individual.

**MSC donor #1**

**MSC donor #2**

**Gene name:**

**Mean FC**

**P value**

**Mean FC**

**P value**

AASDHPPT

0.448528737

1.51867E-20

0.509687321

9.65495E-15

ABCC3

0.753348862

2.75114E-09

0.790355401

1.10105E-07

ACBD3

0.755386173

4.3683E-08

0.728434267

9.189E-08

ACSL4

0.705240784

8.2342E-12

0.751477206

4.52849E-08

ACVR1

0.549556426

3.83184E-30

0.637399886

3.71257E-16

ADCY9

0.743563424

1.48654E-07

0.784342657

1.6777E-07

ADIPOR2

0.75391201

1.05531E-08

0.758042424

1.14193E-07

ADSS

0.679415241

1.33189E-13

0.654053343

1.33195E-13

AFF4

0.753471389

3.61722E-06

0.772795167

3.6429E-06

AGL

0.788355348

8.0296E-05

0.659111429

8.0296E-05

AMMECR1

0.578520981

7.61732E-17

0.639263926

1.42498E-12

ANGEL2

0.574692418

5.61365E-23

0.667913664

1.58482E-07

ANKRD33

0.755805065

1.7699E-07

0.752924042

1.7699E-07

ANTXR1

0.758004925

3.85533E-05

0.712290653

3.85847E-05

AP3D1

0.645946609

1.07353E-09

0.716219488

2.46018E-09

ARF1

0.719535287

0.000153505

0.787322292

0.000153506

ARFIP1

0.569802059

4.97805E-15

0.650617249

2.82241E-11

ARL4C

0.536618354

8.77392E-14

0.720560703

1.10443E-07

B3GALNT1

0.753175847

1.64445E-05

0.795466711

0.000597803

BCL2L2

0.71971978

1.27505E-13

0.76387459

1.05491E-09

BMPR2

0.785402326

2.76668E-05

0.770653217

3.92571E-05

CAMK2G

0.712093314

1.1038E-10

0.79388269

1.27368E-06

CAND1

0.645995806

4.07125E-19

0.710776892

3.0856E-10

CBL

0.597568244

5.68234E-16

0.539778831

1.92284E-09

CBX3

0.781063435

1.52505E-05

0.759088341

1.52505E-05

CBX6

0.595626764

2.31984E-12

0.64379996

2.34782E-12

CCNG2

0.634439518

3.84137E-11

0.638168637

4.28095E-11

CCNY

0.291280531

3.6512E-34

0.426287383

3.6512E-34

CDO1

0.580118874

9.82983E-15

0.398389707

9.82983E-15

CHKA

0.623298203

1.17177E-15

0.592126001

1.17294E-15

CLINT1

0.790307357

7.63648E-06

0.793832837

4.05331E-05

COMMD9

0.674119287

1.04656E-08

0.672923663

1.09453E-08

CRADD

0.499026592

5.30084E-27

0.579646361

8.88623E-24

CXCL12

0.506696688

2.34855E-29

0.427725124

2.19334E-19

DENR

0.616407336

5.36915E-20

0.637283853

2.04606E-12

DGCR2

0.699812503

3.64092E-06

0.576812042

3.64092E-06

DKC1

0.604873901

4.51308E-23

0.629347873

1.24389E-18

DNAJC12

0.706017983

1.41994E-07

0.75789011

2.36824E-06

DUSP3

0.45393947

1.95767E-40

0.547840271

7.7441E-26

DYNC1I1

0.62771337

6.52529E-14

0.78691963

7.34935E-06

DYNLL1

0.764752009

5.30251E-05

0.739429341

5.30252E-05

DYRK1A

0.763743498

0.000616641

0.776864309

0.000617846

EBF1

0.731983965

1.06665E-10

0.748121569

7.78003E-08

ELN

0.343865124

5.09959E-26

0.40473162

5.12135E-26

ESM1

0.468963881

6.92011E-20

0.644150995

1.22109E-11

FAM103A1

0.76085678

5.19477E-08

0.72561009

1.96019E-07

FAM108C1

0.709431679

5.83968E-13

0.694336251

1.1825E-09

FAM122B

0.701343311

7.74577E-11

0.690538211

2.19799E-08

FAM127C

0.478302162

7.64394E-27

0.712201913

3.10809E-13

FAM13A

0.766082344

6.84094E-05

0.794801975

8.78293E-05

FAM13B

0.578227336

7.37305E-20

0.553535593

7.37318E-20

FAM168B

0.761909449

4.11484E-06

0.753920259

4.12671E-06

FAM45A

0.598888759

2.0237E-15

0.689968301

1.12237E-14

FAM53C

0.377831181

6.46702E-37

0.430361207

9.32952E-30

FERMT2

0.553180276

6.49736E-18

0.667914689

6.4974E-18

FGF7

0.776176838

2.04167E-05

0.745717657

0.000242881

FRMD6

0.645479886

9.66693E-19

0.638470552

1.34005E-18

GAS1

0.36544812

1.37055E-20

0.367257416

1.37055E-20

GCLM

0.539774332

1.72643E-17

0.486387122

1.72643E-17

GCSH

0.795121594

6.11241E-05

0.752394432

6.11251E-05

GLRB

0.697444719

1.55035E-15

0.737733251

3.28757E-07

GOLGA1

0.666800395

4.70541E-13

0.759446205

3.49778E-08

GOLT1B

0.331034346

1.18485E-37

0.42938738

2.78966E-21

GPD1L

0.578831471

3.56254E-25

0.646931428

4.21235E-15

GREM1

0.464087862

6.79436E-30

0.4886247

8.84933E-20

HCCS

0.439862886

2.13256E-16

0.555674897

2.13258E-16

HIPK2

0.543864553

1.81777E-12

0.467689673

1.81777E-12

HMG20A

0.689102252

3.44576E-09

0.676682382

3.52094E-09

INSIG1

0.799909834

9.85787E-05

0.680969754

9.85787E-05

IP6K1

0.79072884

0.001334795

0.767391802

0.001519068

ITGA5

0.73947011

2.89386E-08

0.793659897

4.46801E-08

ITGB3

0.585228865

2.90585E-18

0.726277081

4.95738E-09

JARID2

0.763279598

1.01748E-09

0.787014661

1.39017E-05

KIAA0355

0.484162771

2.09195E-35

0.587723243

7.40402E-17

KIAA0494

0.676550187

5.06485E-10

0.695670806

1.61513E-09

KIAA0513

0.743866116

1.51666E-06

0.66196855

1.51666E-06

KIAA1217

0.763906605

1.43887E-08

0.793394301

0.000164439

KIAA1370

0.742244807

8.22596E-06

0.757565672

8.44892E-06

KIAA1644

0.729855323

3.79684E-10

0.670987075

3.79684E-10

KIAA1715

0.621047925

4.98241E-15

0.635300269

7.02054E-09

LDB2

0.596847547

3.22067E-18

0.613684447

9.33313E-17

LMLN

0.798450895

9.34397E-10

0.727049151

9.36253E-10

LPXN

0.580847533

1.00498E-21

0.653015459

7.33461E-16

LUM

0.549463868

7.25228E-15

0.606216055

3.96693E-14

MALT1

0.616126833

1.52289E-18

0.658119414

1.85616E-15

MAPK1

0.605171754

3.55454E-21

0.709979578

1.18613E-09

MED22

0.604077722

9.60283E-14

0.707429177

9.63441E-13

MED4

0.667464311

1.91289E-13

0.755165476

2.22316E-08

METAP2

0.686686289

1.38303E-14

0.691310867

1.20049E-13

METTL7A

0.732644263

4.30254E-08

0.799858932

0.000148494

MEX3C

0.554993444

1.99757E-10

0.613922591

1.99757E-10

MPRIP

0.692147607

1.20572E-11

0.682584089

2.13001E-11

MSN

0.720134022

6.99377E-15

0.73663143

9.30957E-13

MTF1

0.719435384

1.82063E-12

0.772845352

3.46773E-05

MTMR9

0.524708148

3.15515E-24

0.663414962

1.1248E-10

MUM1

0.696716407

1.2655E-11

0.635597463

1.2655E-11

NDFIP1

0.74482556

1.01152E-07

0.718889174

1.39073E-07

NT5E

0.527995725

6.71476E-29

0.515193483

1.79064E-19

OCRL

0.698348753

1.48058E-15

0.678933778

4.35672E-13

OLFML2B

0.770255195

1.04798E-06

0.703964649

1.04798E-06

PAICS

0.536833826

1.97427E-22

0.584926275

8.70756E-13

PCBP1

0.5554641

4.13079E-26

0.593335087

3.25437E-22

PCBP2

0.405708186

2.40152E-42

0.449843263

2.54034E-31

PGM2L1

0.405262841

1.51133E-16

0.471515955

1.51133E-16

PHACTR2

0.791235368

1.87801E-05

0.77297414

2.43771E-05

PIK3C2A

0.56355199

3.05603E-20

0.645487334

2.24143E-11

PITPNM1

0.767212241

4.92005E-09

0.666524314

4.92005E-09

PLEKHO2

0.795860941

4.10975E-05

0.791985348

5.19891E-05

PPP2CB

0.645364426

5.05166E-14

0.659981488

7.87624E-12

PREP

0.557957969

7.69938E-17

0.721210931

5.74405E-07

PRKAR1A

0.620656057

2.99319E-11

0.739214191

3.10062E-11

PRPF18

0.782078268

3.75338E-06

0.775675704

4.32527E-06

PRRG1

0.697724009

8.29926E-10

0.721104279

1.08731E-09

PSMA5

0.48019979

3.31818E-41

0.557934233

2.32088E-20

PURB

0.660753285

1.13289E-20

0.753858269

1.83904E-06

RAB22A

0.373345509

3.6512E-34

0.460137306

1.31409E-18

RAB31

0.453122376

3.72023E-37

0.557265557

4.0039E-37

RAB5B

0.751308324

2.139E-15

0.777938365

9.99987E-10

RAP2C

0.556284633

7.16996E-11

0.574762124

8.29889E-11

RAVER2

0.699222076

1.12627E-06

0.76361622

1.24715E-06

RBBP9

0.588066839

2.11472E-20

0.702683091

4.36049E-18

RCN1

0.617657384

5.18619E-15

0.591184605

5.18619E-15

RNF4

0.725965535

1.44557E-06

0.746743804

1.44637E-06

RPS6KA2

0.569754313

2.18683E-30

0.651804215

2.34261E-18

RRAGD

0.751296156

7.71597E-08

0.762387719

9.44186E-08

RRM2B

0.540697692

2.4794E-20

0.587876342

3.40434E-14

RYBP

0.632126156

1.66277E-15

0.61162307

1.66284E-15

SDHC

0.690981905

1.9711E-12

0.706722043

5.77608E-12

SEL1L3

0.774670595

6.12937E-09

0.713384726

1.04141E-08

SEPX1

0.572634779

2.13766E-12

0.634482824

4.0252E-12

SH3PXD2B

0.670295118

2.38458E-12

0.64318389

2.38475E-12

SLC25A1

0.72483912

5.81427E-08

0.735486811

1.48101E-07

SLC31A1

0.597300642

1.32879E-08

0.659685381

2.88649E-07

SLC35C1

0.708235028

1.39008E-08

0.740345451

2.68131E-08

SLC48A1

0.708668352

5.30479E-08

0.735089188

3.70635E-07

SMG7

0.73179602

2.21771E-10

0.630347547

2.21771E-10

SNX27

0.763868683

8.62517E-05

0.745967598

8.6252E-05

SOCS2

0.710538358

3.58565E-11

0.753901518

1.51454E-07

SORBS2

0.504564418

4.46561E-23

0.652875378

9.394E-13

SPA17

0.629407197

1.45912E-17

0.72444485

1.75326E-08

SRPK1

0.753138994

4.88053E-07

0.794965398

5.562E-07

SRPK2

0.42453148

2.86206E-22

0.534848336

4.0956E-16

SSPN

0.7249099

1.05271E-08

0.780823197

2.01849E-06

STARD13

0.684773368

9.36824E-12

0.769395499

4.60764E-08

STRADA

0.616903723

1.83644E-14

0.731719717

3.10187E-11

TACC1

0.514985069

5.68916E-37

0.702752112

3.4635E-09

TBC1D4

0.52499837

4.53526E-14

0.574797917

4.53526E-14

TBCA

0.405321298

2.06453E-35

0.512635888

2.74869E-26

TCF4

0.722708202

1.627E-08

0.665073599

1.627E-08

TM2D3

0.680831577

7.01868E-12

0.669440896

7.08636E-12

TMEM194A

0.783226127

8.83331E-06

0.793563669

2.24975E-05

TMX1

0.798974851

1.65819E-07

0.779571594

3.24186E-07

TNFRSF10B

0.417453446

3.46623E-28

0.534576585

5.51795E-23

TSPAN31

0.5870376

1.05852E-18

0.712916249

4.82629E-07

TTC31

0.643294492

4.70541E-13

0.668004667

1.38421E-12

TXLNA

0.644584282

1.74181E-21

0.65405203

1.74217E-21

UBE2Z

0.781528492

0.000186775

0.781549479

0.0002183

UNC5B

0.729062534

3.30027E-07

0.732508758

1.13106E-06

USP46

0.726876744

5.20091E-09

0.73845624

1.27129E-07

UVRAG

0.625117954

6.42263E-15

0.745243944

1.28882E-07

VAMP1

0.722025542

5.81427E-08

0.7475754

5.84655E-08

VAMP4

0.761776037

0.000114964

0.749499905

0.000116234

VGLL3

0.565628452

9.87634E-17

0.70337775

6.98332E-13

VPS24

0.546998445

3.67487E-20

0.768309795

3.84681E-06

WASF3

0.708431462

7.96335E-11

0.778969692

3.26255E-07

XG

0.633765503

1.00172E-16

0.727256432

1.18116E-09

XPO5

0.747999664

6.79221E-11

0.777514959

1.27108E-06

ZC3H10

0.752940524

6.25913E-07

0.757721423

6.34555E-07

ZCCHC14

0.474609629

2.2897E-21

0.45393937

2.28971E-21

ZFHX3

0.764055689

9.4176E-10

0.773391137

2.77098E-09

ZMAT3

0.433250579

2.83802E-37

0.742644423

5.21428E-08

ZNF187

0.524653776

8.82714E-21

0.631674001

7.63219E-15

ZNF234

0.751144869

2.06595E-09

0.72395236

1.02689E-06

ZNF295

0.614205723

2.18735E-21

0.730669823

7.19119E-09

ZNF423

0.765431947

4.90223E-09

0.744906267

7.88031E-06

ZNF562

0.694527924

9.49745E-13

0.76280603

3.17379E-09

ZNF770

0.756564023

3.24344E-05

0.773718403

3.73013E-05

ZYG11B

0.52213463

5.9596E-18

0.581993403

8.49512E-14

ZZZ3

0.7941768

7.66843E-05

0.773707883

7.7341E-05

**Supplementary Table 5: miRNAs and siRNAs.**

**Product**

**Annotation within project**

**Pooled of**

**miRNA and siRNA sequences**

miRIDIAN microRNA Mimic Negative Control #2 (CN-002000-01) (GE Healthcare Dharmacon Inc.)

miR-Ctrl

-

UUGUACUACACAAAAGUACUG

MISSION® miRNA Negative Control 1 (HMC0002) (Sigma-Aldrich Chemie GmbH)

miR-Ctrl #2

-

GGUUCGUACGUACACUGUUCA

MISSION® miRNA Negative Control 2 (HCM0003) (Sigma-Aldrich Chemie GmbH)

miR-Ctrl #3

-

CGGUACGAUCGCGGCGGGAUAUC

hsa-miR-1246 mimic (C-301373-00) (GE Healthcare Dharmacon Inc.)

miR-1246

-

AAUGGAUUUUUGGAGCAGG

ON-TARGETplus non-targeting Pool (D-001810-10) (GE Healthcare Dharmacon Inc.)

siCtrl

#1

UGGUUUACAUGUCGACUAA

#2

UGGUUUACAUGUUGUGUGA

#3

UGGUUUACAUGUUUUCUGA

#4

UGGUUUACAUGUUUUCCUA

siGENOME human Set of 4 Upgrade (MU-007670-02) (GE Healthcare Dharmacon Inc.)

siPRKAR1A

D-007670-02

CGAGACAGCUAUAGAAGAA

D-007670-04

GAUAAUGAGAGAAGUGAUA

D-007670-05

UACGGUAGCUGAUGCAUUG

D-007670-18

GUGGGAACGUCUUACGGUA

siGENOME human Set of 4 Upgrade (MU-003599-03) (GE Healthcare Dharmacon Inc.)

siPPP2CB

D-003599-03

GUAAGCAGCUGAACGAGAA

D-003599-04

CACGAAAGCCGACAAAUUA

D-003599-06

AAAGGUGCGUUAUCCAGAA

D-003599-19

UAGCAUUAAAGGUGCGUUA

**Supplementary Table 8**: **Cloning and mutagenesis primer sequences. A.** List of primer sequences used for cloning of 3’UTRs into psiCHECK2 vector. Directions are given as forward (FW) and reverse (RV). **B.** List of primers used for mutagenesis of miR-1246 binding sites in *PRKAR1A* and *PPP2CB* 3’UTRs and *PRKAR1A* open reading frame (ORF). The cloned fragment of the *PRKAR1A* 3’UTR contained a small part of the *PRKAR1A* ORF due to close proximity of the predicted miR-1246 binding site in the *PRKAR1A* 3’UTR to the ORF. The cloned ORF fragment carried a potential binding site for miR-1246 without biological relevance and was mutated for control conditions (detailed information in supplemental materials).

**A) Primer sequences for 3'UTR cloning**

**Gene**

**Primer ID. direction and restriction site**

**Primer sequence (5' -> 3')**

PRKAR1A 3'UTR

PRKAR1A_FW_XhoI

TACTCGAGCATGCTCAGACATCCTCAAAC

PRKAR1A_RV_NotI

ATGCGGCCGCCTTGGCAAACAACAACTCTGG

PPP2CB 3'UTR

PPP2CB_FW_XhoI

TACTCGAGAACCTGCCTTTGTATGTGGA

PPP2CB_RV_NotI

ATGCGGCCGCTGCTGAGTACACCAAATAGGAT

**B) Primer sequences 3'UTR mutagenesis**

**Mutagenesis**

**Primer ID. direction**

**Primer Sequence (5' -> 3')**

PRKAR1A 3'UTR mutagenesis of potential miR-1246 binding site in ORF fragment

PRKAR1A_FW_mut_ORF

CCTCAAACGAAACATCCAGCTCAGCAACAGTTTTGTGTCACTG

PRKAR1A_RV_mut_ORF

CAGTGACACAAAACTGTTGCTGAGCTGGATGTTTCGTTTGAGG

PRKAR1A 3'UTR mutagenesis of miR-1246 binding site in 3'UTR

PRKAR1A_FW_mut3'UTR

CCTTTTCTCCTCTCCCCATAGGATGCTTCACTCATGCAAAC

PRKAR1A_RV_mut3'UTR

GTTTGCATGAGTGAAGCATCCTATGGGGAGAGGAGAAAAGG

PPP2CB 3'UTR mutagenesis of miR-1246 binding site in 3'UTR

PPP2CB_FW_mut3'UTR

CAAGCTAACTTCCACTAAAGGTTTATCCTTTATTTTATTG

PPP2CB_RV_mut3'UTR

CAATAAAATAAAGGATAAACCTTTAGTGGAAGTTAGCTTG
